# Bioactive metallic nanoparticles for synergistic cancer immunotherapy

**DOI:** 10.1016/j.apsb.2025.02.022

**Published:** 2025-02-21

**Authors:** Lulu Wang, Demin Lin, Muqing Li, Yu Jiang, Yanfang Yang, Hongliang Wang, Hongqian Chu, Jun Ye, Yuling Liu

**Affiliations:** a State Key Laboratory of Bioactive Substance and Function of Natural Medicines, Institute of Materia Medica, Chinese Academy of Medical Sciences & Peking Union Medical College, Beijing 100050, China; b Beijing Key Laboratory of Drug Delivery Technology and Novel Formulation, Institute of Materia Medica, Chinese Academy of Medical Sciences & Peking Union Medical College, Beijing 100050, China; c Translational Medicine Center, Beijing Chest Hospital, Capital Medical University/Beijing Tuberculosis and Thoracic Tumor Research Institute, Beijing 101149, China

**Keywords:** Cancer immunotherapy, Metallic nanoparticles, Metal immunotherapy, Immunomodulation, Tumor microenvironment, Cold tumors, cGAS–STING, Immunogenic cell death

## Abstract

Cancer immunotherapy has emerged as a promising strategy. However, low response rates and immune-related side effects have plagued immunotherapy. Metallic nanoparticles, utilizing metals as their framework, are gaining prominence in cancer immunotherapy. Metal ions have shown the ability to modulate immune status by activating the cGAS–STING pathway and inducing immunogenic cell death (ICD), thereby enabling multidimensional activation of immunotherapy. Metallic nanoparticles offer significant advantages in cancer immunotherapy, leading to their increasing use in enhancing therapeutic outcomes. In view of the ever-increasing research on metallic nanoparticles, this review presents the construction, characterization, and enhanced cancer immunotherapeutic effects of different types of metal nanosystems from the perspective of the immunoregulatory mechanisms of metal ions. We delve into the current limitations and future directions of metallic nanoparticles in this rapidly evolving field. To the best of our knowledge, this review offers the most up-to-date and systematic analysis of metallic nanoparticles in immunotherapeutic applications. It is anticipated that this review of metallic nanoparticles will inspire a more refined and intelligent design of metallic nanoparticles for future research, paving the way for advancing their clinical applications.

## Graphical abstract

**Figure:**
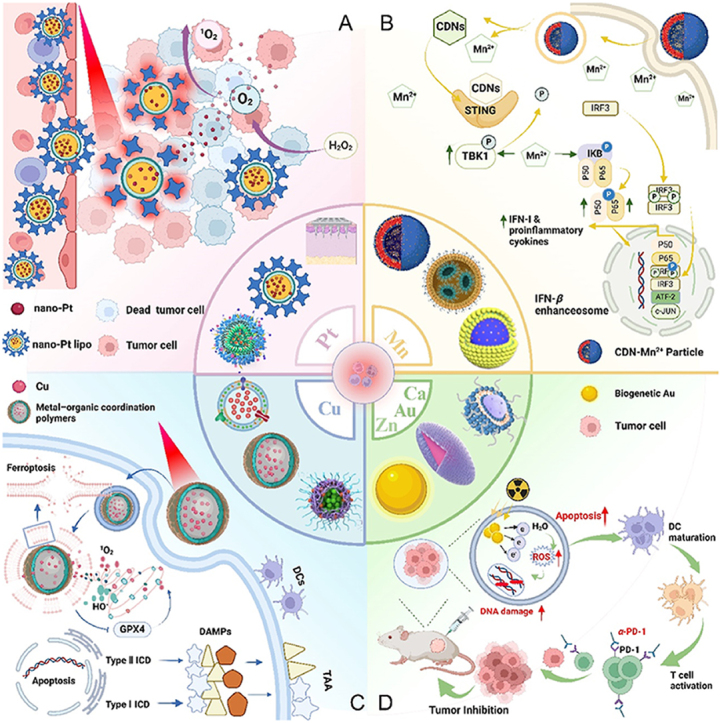

## 1 Introduction

Conventional tumor treatments, including surgical resection, chemotherapy, and radiotherapy, which in clinical practice can directly kill tumor cells and cause the primary tumor to subside^1^, ^2^, ^3^. However, the emergence of an immunosuppressive tumor microenvironment (iTME) and immune escape during tumor progression significantly hinder the efficacy of these traditional modalities^4^. iTME refers to the depletion or remodeling of the relevant effector cells in the microenvironment after prolonged tumor antigen stimulation and immune activation, which fails to perform its normal function and promotes the malignant character of the tumor^5^. With a deeper understanding of tumor biology and human immunity, immunotherapy has rapidly energized as a new generation of tumor treatment after conventional treatment, offering significant potential for clinical application. For example, tumor vaccines, cytokine therapies, and adoptive T-cell transfer that stimulate the immune system; immune checkpoint inhibitors (ICBs) that eliminate or inhibit immunosuppressive factors, such as cytotoxic T lymphocyte associate protein-4 (CTLA-4) inhibitors and programmed death receptor/ligand 1 (PD-(L)1) inhibitors. Despite exciting clinical results, immunotherapy still suffers from many shortcomings. Clinical data show that, depending on the immunogenicity of the tumor, only a minority of patients respond well to ICB^6^^,^^7^. Non-immunogenic tumors with low T-cell infiltration or PD-L1 expression are unlikely to respond to ICB therapy^8^^,^^9^. Therefore, converting non-immunogenic tumors into immunogenic tumors may be a solution to address low clinical response rates, where immunogenicity plays a key role.

Metal immunotherapy is expected to open a new path in the field of tumor therapy. Metal ions play a significant role in the immune process by interacting with immunosensors and ion transporters. They also influence enzymes and downstream effector proteins through catalytic or regulatory effects. Metals and immune regulation are naturally strongly linked. On the one hand, metal ions can enhance tumor immunogenicity through modulation of various aspects of immunotherapy. For example, Mn^2+^ modulates the structure and function of immune-associated proteins, enhancing the sensitivity of cyclic GMP–AMP synthase (cGAS) for DNA detection and promoting the synthesis of the cyclic GMP–AMPP (cGAMP) tens of thousands of folds. In contrast, cGAMP activates tumor immunogenicity by acting as a second messenger for the activation of STING and the induction of type I interferon (IFN)^10^. Following T cell receptor activation, Ca^2+^ further promotes T cell activation and enhances cellular immune response. In addition, platinum-based drugs can alleviate immune checkpoint-mediated immunosuppression^11^. Zn^2+^ increases tumor immunogenicity by triggering ICD and mitigating suppression within the tumor immune microenvironment^12^. In contrast, cGAMP activates tumor immunogenicity by acting as a second messenger for the activation of STING and the induction of IFN. Moreover, metal complexes can provide a large number of metal-drug combination solutions by modulating multiple variables (metal, ligand, and metal–ligand interactions), expanding possibilities for drug design^13^. On the other hand, tumor immunity also presents new opportunities and ideas for the development of metallotropic drugs. The need for deeper molecular mechanisms with more sophisticated designs has increased as tumor immunity continues to evolve. Based on the molecular space provided by coordination and synthetic chemistry, novel metallodrugs in combination with immunological tools such as ICB therapy and cytokine therapy are opening up new avenues for the clinical treatment of tumors.

Despite the aforementioned advantages of metal ions in immunomodulation, they often exhibit limitations such as poor pharmacokinetics, low levels of target accumulation, off-target reactions, and drug resistance, hindering their efficacy and clinical translation. Nanomedicine emerges as a powerful tool to address these shortcomings^14^. At the same time, if we want to achieve effective cancer immunotherapy only improving the tumor immunogenicity is not enough, but also need to further overcome the immunosuppressive TME, but improving the tumor immunogenicity is undoubtedly the key point to enhance the effect of immunotherapy. Nanocarriers optimize synergistic effects by modulating the pharmacokinetics, drug concentration, drug ratio, and release rate of bioactive payloads through various features. These include coordinating drug interactions within the core of the nanocarrier, controlling the release chemistry of the drug-polymer linker, and attaching drugs or antibodies to the nanocarrier surface. These properties enable the simultaneous delivery of multiple drugs, including nucleic acids, within a single nanopharmaceutical. Furthermore, inorganic nanomaterials, particularly metal–organic frameworks, show promising applications in immunotherapy. For example, manganese dioxide and zinc oxide can serve as nanocarriers for photosensitizers, inducing apoptosis, autophagy, and necrosis of tumor cells in phototherapy^15^^,^^16^. Unlike conventional nanomedicines that directly target tumor cells, nanoplatforms tailored for cancer immunotherapy have a wider choice of targets (*e.g.* T cells, macrophages, lymphoid tissues, etc.).

**Figure 1:**
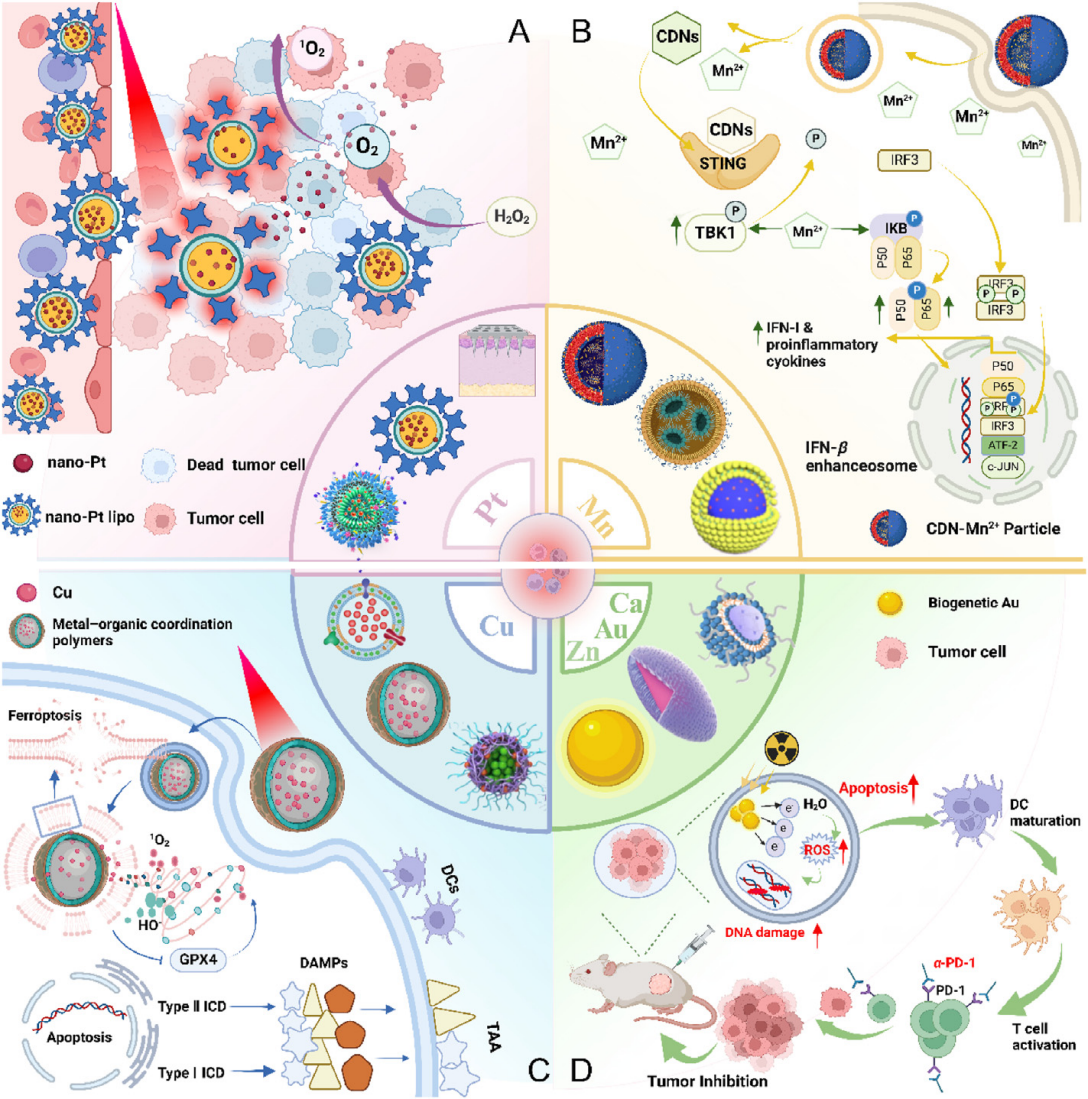
Schematic diagram of nanocarrier-assisted immunotherapy. (A) Nano-Pt lipo selectively accumulates at the tumor, breaking down H_2_O_2_, providing oxygen, and enhancing the effects of photoconductive radiotherapy. The ^1^O_2_ disrupts the liposome membrane and releases ultra-small nano-Pt, facilitating tumor penetration and chemotherapy. (B) CMP boosts STING activation: CMP promotes cellular uptake of CDNs and Mn^2+^; Mn^2+^ augments CDN-induced STING activation *via* STING-independent TBK1 and p65 phosphorylation, STING-dependent IRF3 phosphorylation, and assembly of the IFN-*β* transcriptional enhanceosome. (C) Upon irradiation, internalized MOCPs escape ROS-damaged endo/lysosomes and release cytotoxic substances. MOCPs initiate ferroptosis in response to uncontrolled lipid peroxidation and enhance typeⅠICD and oxidative storm typeⅡICD in response to prolonged exposure to DAMPs. (D) Biogenetic Au induces ROS generation and DNA damage upon radiation exposure, ultimately leading to apoptosis. Tumor antigens released were captured by DCs, activating a systemic immune response. In combination with immune checkpoint blockade, the depletion of T cells by PD-L1 was further mitigated. CMP: cyclic dinucleotides-Mn^2+^ Partial; DAMPs: damage associated molecular patterns; DC: dendritic cell; GPX4: glutathione peroxidase 4; IFN-*β*: interferon-*β*; IRF3: interferon regulatory factor 3; MOCPs: metal–organic coordination polymers; PD-1: programmed cell death protein 1; ROS: reactive oxygen species; STING: stimulator of interferon genes; TAA: tumor-associated antigen; TBK1: TANK binding kinase 1; This image is created with BioRender.

This review aims to synthesize current knowledge on the role of metallic nanoparticles in cancer immunotherapy, focusing on their construction, immunomodulatory mechanisms, and clinical potential (Fig. 1). In particular, we have elucidated the mechanisms by which metal ions regulate tumor immune statutes, including Mn^2+^ activation of the cGAS–STING pathway, K^+^ activation of the nucleotide-binding oligomerization domain-like receptor protein 3 (NLRP3) inflammasome, Pt-induced ICD and Ca^2+^-induced cellular pyroptosis. Furthermore, we highlight the multifunctional effects of metallic nanoparticles in immunotherapy, *e.g.*, Mn-based nanoparticles can serve as both nanocarriers for delivering drugs and immune activators, stimulating immune responses. Additionally, an increasing number of metallic nanoparticles are being developed to enhance cancer immunotherapeutic effects, either independently or in combination with other immunotherapies. For example, Pt-based nanoparticles can modulate the immune response by inducing ICD and synergistically enhancing immunotherapeutic effects when combined with photothermal therapy (PTT) and photodynamic therapy (PDT) therapy. We expect that this review will provide guidance and support for the development and application of metallic nanoparticles for immunotherapy, opening up new avenues for next-generation cancer immunotherapy and ultimately benefiting patients.

## 2 Cancer immunotherapy

Unlike other cancer therapies that directly kill tumor cells, immunotherapy works by harnessing and coordinating the patient's immune system^17^^,^^18^. This unique approach has garnered significant attention as a novel strategy for tumor treatment, fundamentally transforming the landscape of tumor therapy.

### 2.1 Definition of cold and hot tumors

Understanding the intricate interplay between tumors and the immune system has paved the way for a stepwise treatment strategy for patients. Based on the spatial distribution of cytotoxic immune cells within the TME, tumors can be classified into three basic immune phenotypes: immune-inflamed, immune-excluded, and immune-desert^19^^,^^20^. Among these, the immune-inflamed phenotype, also known as “hot tumors”, is characterized by high T-cell infiltration, enhanced interferon-gamma (IFN-*γ*) signaling, PD-L1 expression, and increased tumor mutational burden (TMB)^21^. Immune-excluded and immune-desert phenotypes are called “cold tumors”. Immune-excluded and immune-desert tumors have been termed “cold tumors” and are essentially characterized by poor T-cell infiltration^22^, reduced expression of MHC class I, PD-L1, and TMB^21^, and the presence of immune-suppressive cell populations (*e.g.* tumor-associated macrophages (TAMs) and T-regulatory cells (Tregs) and myeloid-derived suppressor cells (MDSCs))^23^. The immunosuppressive conditions that exist in these “cold tumors” create a favorable environment for tumor formation and development, known as the immunosuppressive microenvironment.

### 2.2 Means to transform hot and cold tumors

**Figure 2:**
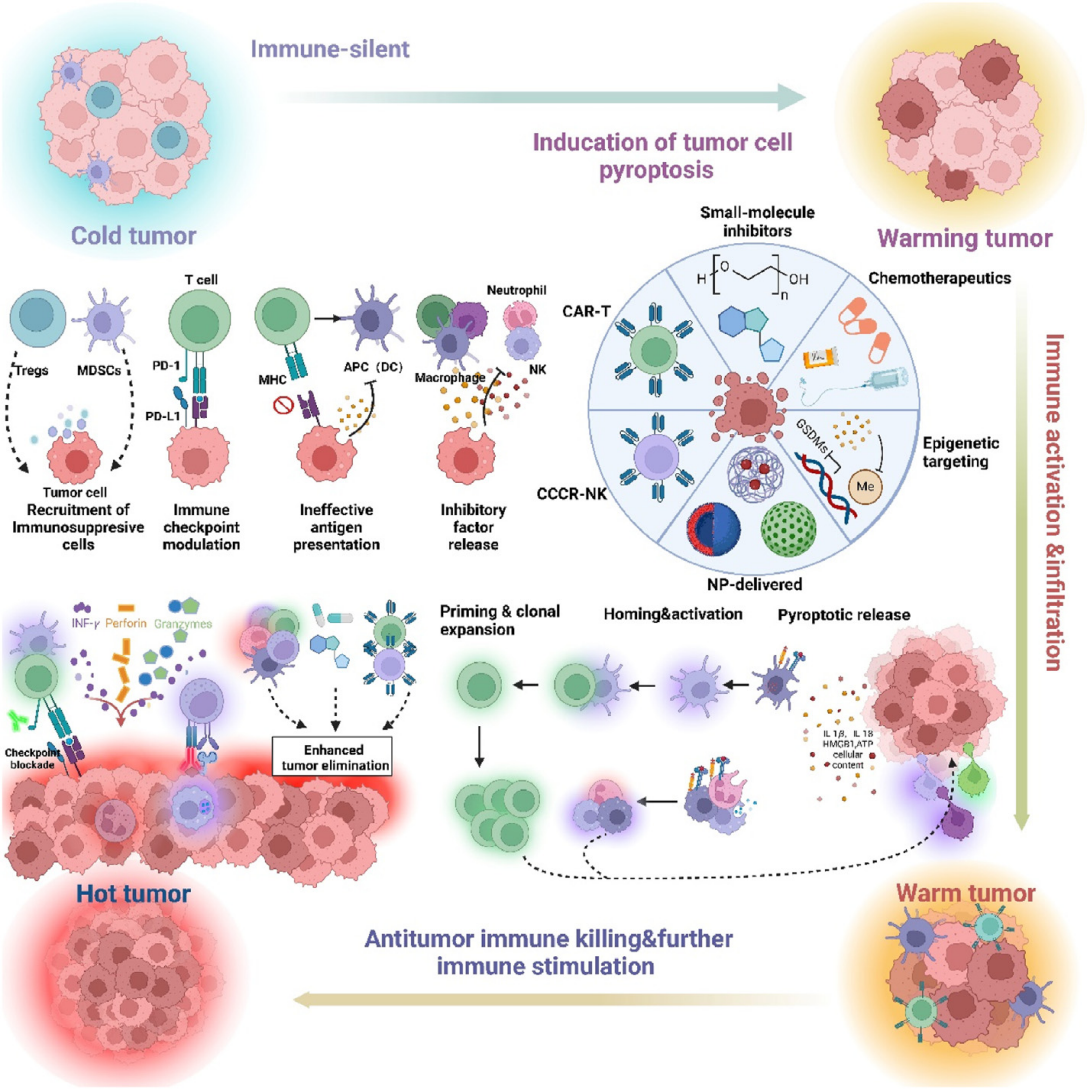
The change from a “cold” tumor to a “hot” tumor. “Cold tumor”: tumor cells establish an immunosuppressive microenvironment to evade immune surveillance and destruction by recruiting immunosuppressive cells, upregulating immune checkpoint proteins, impairing antigen presentation. “Warming tumor”: various strategies induce pyroptosis in tumor cells and “heat” tumors from an immune-evasive state. “Warm tumor”: pyroptotic tumor cells release pro-inflammatory cytokines and immunogenic material that stimulate activation and recruitment of immune cells. “Hot tumor”: infiltrating immune cells recognize and eliminate tumor cells, potentially leading to a positive feedback loop that enhances tumor-specific immunity. CAR-T: chimeric antigen receptor T cell; CCCR-NK: chimeric costimulatory converting receptor natural Killer cell; DC: dendritic cell; GSDMs: gasdermin proteins; HMGB1: high-mobility group box protein 1; IFN-*γ*: interferon-gamma; IL: interleukin; MDSCs: myeloid-derived suppressor cells; MHC: major histocompatibility complex; NK: natural killer cell; NP: nanoparticle; PD-L1: programmed death-ligand 1; PD-1: programmed cell death protein 1; TNF-*α*: tumor necrosis factor-alpha; Tregs: regulatory T cells. This image is created with BioRender.

In “hot tumors”, the internal environment is infested with large numbers of T-cells, and when ICBs lift the suppression of the immune checkpoints, the T-cell immune response against the tumor is reactivated, resulting in an immunotherapeutic effect. However, in “cold tumors”, immunotherapies such as ICBs are ineffective due to insufficient chemokine secretion in the TME and the low chemotactic capacity of circulating T cells. To transform “cold tumors” into “hot tumors”, various tumor immunotherapies have emerged, such as ICB therapy, chimeric antigen receptor cell (CAR)-T therapy, vaccine therapy, and cytokine therapy achieving impressive clinical results (Fig. 2). One of these therapies, ICB, stimulates the immune response by blocking inhibitory pathways and has proven to be one of the most effective strategies for the clinical treatment of several types of tumors^24^. CAR-T therapy, a genetically engineered strategy that fuses antigenic target regions with T-cell receptor signaling chains and co-stimulatory molecules, has made significant advancements in clinical practice, particularly in the treatment of hematological malignancies^25^^,^^26^. Tumor vaccines typically contain immune adjuvants and tumor-specific antigens that induce an antigen-specific immune response and even provide an immune memory effect to inhibit tumor recurrence^17^^,^^18^. Furthermore, several immunotherapies have been approved by the US Food and Drug Administration (FDA) for their clinical efficacy. For example, nivolumab, a therapeutic PD-1 antibody for the treatment of a variety of cancers, including melanoma and non-small cell lung cancer, has been approved by the FDA^27^, ^28^, ^29^, ^30^. The FDA has also approved ipilimumab, a therapeutic antibody for the treatment of CTLA-4 in melanoma^22^.

Despite the tremendous progress made in immunotherapy, there are still a number of challenges to its clinical translation, including but not limited to efficacy and safety concerns^22^. Although ICB therapies have achieved encouraging clinical results, their low clinical response rates and the risk of immune-related adverse events continue to limit their further development^31^. CAR-T therapy, while demonstrating great potential in treating lymphomas and leukemias, faces significant limitations in treating solid malignancies^32^. In addition, cytokine release syndrome, off-target toxicity and extremely high treatment costs pose major obstacles to the widespread use of CAR-T therapies^33^, ^34^, ^35^. In conclusion, the development of safe and effective adaptive immunotherapeutic strategies is crucial to overcome these challenges and realize the full potential of immunotherapy in cancer treatment.

## 3 Metal ion reactions and immunomodulation

Growing evidence highlights the critical roles of metallic elements in the human immune response. Immune signaling is a central process in the immune response. In recent years, various metal ions, such as Ca^2+^, Zn^2+^, Mn^2+^, and Fe^2+^/Fe^3+^, have been proved to directly activate immune cells. How do metal ions support immune-mediated cancer therapy? Metal ions are classified according to their association with antitumor immune mechanisms and present five aspects of metal immunotherapy in tumors: metal ions activating the cGAS–STING pathway; metal ions activating NLRP3 inflammatory vesicles; metal ions inducing ICD; metal ions polarizing M1 macrophages; metal ions inducing cellular pyroptosis. The mechanisms involved in activating the immune response by metallodrugs are summarized in Table 1^10^^,^^36^, ^37^, ^38^, ^39^, ^40^, ^41^, ^42^, ^43^, ^44^, ^45^, ^46^, ^47^, ^48^.

**Table 1:** Effects and applications of representative metal ions^36^.

Metal type	Mechanism	Appliance	Ref.
Mn	Sensitization of cGAS and its aptamer STING; increasing STING–cGAMP binding affinity; releasing I IFN	Innating immune activator	10
Pt	Enhancing antigen presentation and T-cell killing; increasing expression of PD-L1 by tumor cells	Cisplatin; used in combination with immune checkpoint antibodies; ICD inducer	39
Fe	Promoting IFN-*γ* secretion by CD8^+^ T cells; down-regulating the expression of SLC3A2 and SLC7A11	Promoting ferroptosis in tumor cells, combined with ICB therapy	40
Zn	Induction of pyroptosis *via* caspase-1/GSDMD and caspase-3/GSDME pathways	Promoting pyroptosis in tumor cells	41
Ca	Promoting CD3 phosphorylation, TCR signaling; and increasing T-cell sensitivity.	Store-operated Ca^2+^ entry (SOCE) block	42
Au	Activating TLR3 signaling; stimulating immune cells to secrete key inflammatory cytokines; inhibiting of DNA-binding activity of NF-*κ*B	High-efficiency mediated photothermal conversion; coupled with immune checkpoints; sensitizers for radiotherapy	44
Ag	Inhibition of ATP production and DNA replication; downregulation of TNF-*α* and IL-6 expression	Antibacterial agent	46
K	Functional calorie restriction; inhibition of autophagy phage	Adoptive cell transfer therapy	47
Mg	Increasing LFA-1 signal; interacting directly with IL-2-induced T cell kinase (ITK), and promoting its activation	Combined with PD-1 blockade; target Mg^2+^ transporters	48

cGAS, cyclic GMP-AMP synthase; STING, stimulator of interferon genes; STING–cGAMP, cyclic GMP-AMPP; I IFN, iInterferon; PD-L1, programmed cell death 1 ligand 1; ICD, immunogenic cell death; IFN-*γ*, interferon-*γ*; ICB, immune checkpoint blockade; SLC3A2, solute carrier family 3 member 2 Gene; SLC7A11, solute carrier family 7 (anionic amino acid transporter light Chain, Xc- System), member 11; GSDMD, gasdermin D Gene; GSDME, gasdermin E; TCR, T cell receptor; TLR3, Toll-like receptor 3; NF-*κ*B, nuclear factor kappa-light-chain-enhancer of activated B cells; ATP, adenosine triphosphate; DNA, deoxyribonucleic acid; TNF-*α*, tumor necrosis factor-*α*; IL-6, interleukin- 6; IL-2, interleukin-2.

### 3.1 Metal ions activating the cGAS–STING pathway

**Figure 3:**
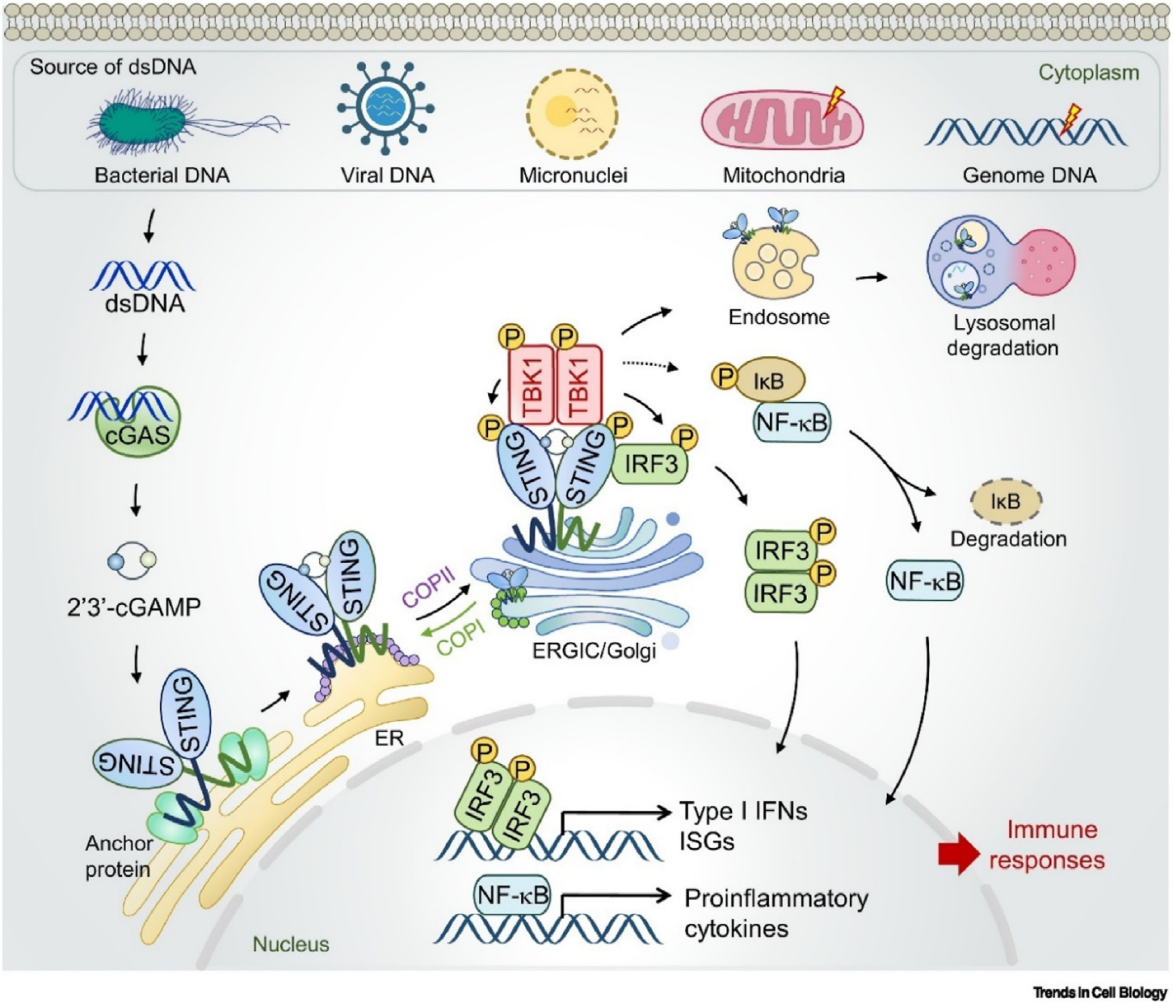
Signaling mechanism of the canonical cGAS–STING pathway^49^. Reprinted with the permission from Ref. 49. Copyright 2022, Elsevier.

The innate immune system, acting as the body's first line of defense, plays a crucial role in initiating and maintaining T-cell responses. The cGAS–STING pathway, a key component of innate immunity, holds significant potential to enhance cancer immunotherapy A series of recent studies have found that the cGAS–STING pathway, which senses cytoplasmic DNA within (paracrine) macrophages and dendritic cells (DC) in tumor tissues, can be activated by tumor cell-derived DNA to promote the maturation of antigen-presenting cells, which in turn deliver tumor-specific antigens to T cells and play a crucial role in resurrecting the body's anti-tumor immune response^49^. The cGAS–STING pathway is composed of cGAS, STING, and a series of downstream signal transduction junctions and effector molecules (Fig. 3). When immune cells ingest pathogens or cellular debris, cytoplasmic cGAS recognizes the DNA present^50^, ^51^, ^52^. Upon binding to DNA, cGAS undergoes a conformation change, acquiring catalytic activity. Activated cGAS catalyzes the conversion of guanosine triphosphate (GTP) and adenosine triphosphate (ATP) into cyclic GMP–AMP (cGAMP), a secondary messenger crucial for STING activation. CDN can bind and activate the STING protein in the endoplasmic reticulum. Activated STING proteins recruit and activate Tank-Binding Kinase 1 (TBK1) and Interferon Regulatory Factor 3 (IRF3)^50^^,^^53^, promoting the expression of downstream IFN genes, leading to further immune activation. Mg^2+^ can act as a catalytic cofactor for cGAS, catalyzing cGAMP to form^54^. In contrast to cytoplasm Mg^2+^, Mn^2+^ is released from the mitochondria and the Golgi during infection with DNA viruses. Notably, Mn^2+^ directly activates cGAS, generating the secondary messenger cGAMP at low concentrations of double-stranded DNA^55^, or independently of double-stranded DNA^56^. CGAMP activates the immune response by inducing the expression of IFN and other cytokines. Furthermore, Mn^2+^ has been shown to enhance the sensitivity to DNA and the enzymatic activity of cGAS and promote the binding of cGAMP to STING, thereby enhancing the effects of several components of the cGAS–STING pathway. The release of Mn^2+^ into the cytoplasm hyperactivates the cGAS–STING pathway, greatly enhancing its ability to respond to cytoplasmic DNA and activate it even at levels of DNA that were originally inactive^57^. Zn^2+^ also enhances cGAS activity by promoting the phase transition of cGAS and stabilizing the cGAS-dsDNA complex^58^. By promoting the phase transition of cGAS and stabilizing the cGAS-dsDNA complex, Co^2+^ and Zn^2+^ were also shown to increase cGAS activity^58^. Co^2+^ also promotes cGAS binding to STING on the ER, which recruits interferon regulatory factor 3 (IRF3) and stimulates transcriptional expression of IFN and other immunostimulatory genes. In addition, Pt may enhance the immunotherapeutic effect by damaging the DNA in tumor cells and releasing it into the cytoplasm, where it binds to cGAS and activates the cGAS–STING pathway^59^^,^^60^. Table 2 summarizes the role of metal ions in the cGAS–STING pathway^54^^,^^55^^,^^57^, ^58^, ^59^, ^60^. In addition to directly mediating the activation of the STING pathway, some metal ions are indirectly involved in STING activation. For example, intracellular endoplasmic reticulum stress leads to Ca^2+^ translocation, which supports STING activation^61^.

**Table 2:** Role of metal ions in the cGAS–STING pathway.

Metal ion	Role in the cGAS–STING pathway	Ref.
Mn^2+^	Enhancing DNA sensitivity, enzymatic activity of cGAS, and promoting binding of cGAMP to STING.	57
Mg^2+^	Acting as a catalytic cofactor for cGAS and catalyzing the formation of cGAMP.	54
5Zn^2+^	Promoting phase transition stabilization of cGAS.	58
Co^2+^	Promoting cGAS binding to STING on the ER.	55
Pt	Promoting the release of DNA from tumor cells, which binds to cGAS.	59,60

STING, stimulator of interferon gene; DNA, deoxyribonucleic acid; cGAS, cyclic guanosine monophosphate-adenosine monophosphate synthase; cGAMP, cyclic guanosine monophosphate-adenosine monophosphate; ER, endoplasmic reticulum.

### 3.2 Metal ions that activate NLRP3 inflammatory vesicles

Metal ions have been shown to mediate the activation of innate immune responses by inflammatory vesicles. Inflammasome is an oligomeric protein complex that matures IL-1*β* and IL-18*β* by activating caspase-1, which in turn generates a pro-inflammatory response^62^. Ca^2+^ has been shown to promote the assembly of NLPR3 inflammatory vesicle components *via* the G protein-coupled receptor calcineurin. When stimulated, calcium receptors can cause an increase in cytoplasmic Ca^2+^ signaling by activating phospholipase C and reducing cyclic AMP levels. Both signals contribute to the increased formation of the NLPR3 inflammasome. Thus, in cells at the site of infection and necrosis, Ca^2+^ can act as a damage-associated molecular patterns (DAMPs) signal released by damaged cells to activate NLRP3 inflammatory vesicles^63^. Also, sodium and potassium can induce the formation of NLRP3 inflammasomes by inducing intracellular ion imbalance. Due to the active efflux of potassium ions and the permeability of cell membranes to potassium ions, it has been demonstrated that the depletion of intracellular potassium ions is necessary and sufficient for activating the NLRP3 inflammasome. The influx of sodium ions leads to an imbalance in the sodium/potassium gradient and promotes potassium efflux, which activates the NLRP3 inflammatory vesicles^64^. Similarly, persistent Zn^2+^ deficiency can lead to lysosomal instability, stimulation of NLRP3 inflammasomes, and secretion of IL-1*β*. Mitochondrial damage is also associated with inflammasome activation and a variety of metal ions may be mediators of this process. Mn^2+^ can act as an amplifier of NLRP3 inflammatory vesicle signaling, leading to mitochondrial defects in microglial cells, further increasing the release of apoptosis-associated speck-like protein-containing exosomes and exosome-mediated inflammatory vesicle activation^65^.

### 3.3 Induction of cell death by metallic ions

ICD is the process by which tumor cells undergo apoptosis in response to an external stimulus, transforming from non-immunogenic to Immunogenic, mediating the body's anti-tumor immune response. The specific mechanism is the release of DAMPs from tumor cells after adequate stress pressure drives them. DAMPs mainly contain calreticulin (CRT), adenosine triphosphate (ATP), high mobility group protein B1 (HMGB1), and heat shock protein (HSP)^66^. DAMPs molecules send out “eat me” and “find me” signals. First, there is the “eat me” signal, in which CRTs in dying cells are exposed to tumor-specific or tumor-associated antigens (TAAs), which trigger phagocytosis by antigen-presenting cells (APCs)^67^. ATP and HMGB1 are then released as “find me” signals, which promote DC maturation and tumor antigen presentation. Activated DCs migrate into the draining lymph nodes and activate the initial T cells to cross-present tumor antigens from MHC-I and II molecules to CD8^+^ and CD4^+^ T cells^68^. The activated cytotoxic T lymphocyte (CTL) infiltrates the tumor tissue and kills cancer cells, usually creating an immune memory^69^. ICD also promotes the release of IFN and chemokines, thereby facilitating the recruitment of APCs and T cells and further activating immunity. Immunotherapy has been limited by inadequate response rates, and there is an urgent need to develop effective ICD-inducing agents that convert “cold” tumors into “hot” tumors and increase tumor immunogenicity.

ICD can be induced by a variety of stressors, including conventional pharmaceutical chemotherapeutic agents (*e.g.*, anthracyclines, DNA-damaging agents), intracellular pathogens, targeted anticancer agents (*e.g.*, the tyrosine kinase inhibitor crizotinib), and physiotherapy (*e.g.*, radiotherapy radiation and extracorporeal photochemotherapy)^70^^,^^71^. Among Pt drugs, oxaliplatin directly induces ICD in tumor cells, whereas cisplatin requires an additional inducer to trigger its immunogenicity^72^. Both Pt drugs induce CRT translocation and release ATP, HSP70, and HMGB1. Oxaliplatin targets the nuclear DNA to inhibit DNA synthesis, prevent transcription, and inhibit mismatch repair^73^. In addition, other types of Pt-based drugs have been found to have ICD-inducing characteristics. For example, Pt–NHC is a unique cyclic metal complex that targets the endoplasmic reticulum and induces endoplasmic reticulum stress (ERS) *via* protein kinase R-like endoplasmic reticulum kinase (PERK)^74^. PtII N-heterocyclic carbene complexes, as the first small-molecule chemotherapeutic agents, also show the characteristics of type II ICD inducers, inducing oxidative stress, CRT exposure, and HMGB1 and ATP release^75^^,^^76^. The homeostasis of Ca^2+^ and Cl^–^ in the human body is tightly regulated, and several harmful factors can lead to an imbalance in Ca^2+^ homeostasis and disruption of its distribution, resulting in abnormally high intracellular Ca^2+^ concentrations, that is, Ca^2+^ overload. Ca^2+^ overload causes irreversible cellular damage by inducing impaired mitochondrial oxidative phosphorylation, which decreases mitochondrial membrane potential and ATP content in tissues and activates phospholipases and proteases in the cytoplasm^77^. At the same time, calpain activation leads to the inward flow of extracellular Ca^2+^ through Ca channels, which in turn causes Cl^–^ inward flow and, ultimately, cell death^78^. Therefore, disruption of the intracellular Ca^2+^ concentration balance in tumor cells leads to an abnormally high intracellular Ca^2+^ concentration, which in turn induces ICD in tumor cells.

In addition to the metal ions mentioned above that can induce ICDs, there are also physical methods to induce ICDs. Rapid changes in the tumor temperature mediated by external stimuli (*e.g.*, lasers and magnetic fields) can also induce effective ICDs. As a result, many studies have used PDT and PTT synergistically with metallodrugs to induce ICD and enhance anticancer immunotherapy.

Metallic nanoparticles induce cellular focal death in addition to ICD. Cellular pyroptosis, or cellular inflammatory necrosis, is programmed cell death triggered by inflammatory vesicles^79^. Cellular pyroptosis is characterized by cellular expansion until the cell membrane ruptures, leading to the release of intracellular contents, which triggers an intense inflammatory response. The onset of cellular pyroptosis depends on inflammatory caspase enzymes, mainly caspase-1, 4, 5, and 11, and the protein gasdermin (GSDMs) family^80^. Briefly, activated caspase enzymes cleave GSDM, releasing its N-terminal structural domain. This structural domain binds to membrane lipids and punches the holes in the cell membrane. Cellular osmotic pressure changes and swells until the cell membrane ruptures^81^^,^^82^. There are two main mechanisms of cell lysis: the classical caspase-1-dependent pathway and the non-caspase-1-dependent pathway. The cleavage of both mechanisms leads to the release of pro-inflammatory cytokines such as IL-1*β* and IL-18, which can exacerbate local or systemic inflammation^83^. Metal ions, such as Ca^2+^ and Zn^2+^, can induce pyroptosis in tumor cells by disrupting their balance of metal ions. Ca^2+^ induces tumor cell pyroptosis primarily through the GSDME pathway, whereas Zn^2+^ induces tumor cell pyroptosis primarily through the GSDMD pathway.

### 3.4 Reprogramming of immune cells by metallic ions

TAMs are an important component of the TME and contain two mutually polarizable subtypes: M1 and M2^84^. The two main features of M1-type TAMs are the secretion of multiple pro-inflammatory cytokines and the efficient presentation of antigens. M1-type TAMs can be induced by cytokines such as IFN, colony-stimulating factor (CSF), and tumor necrosis factor (TNF), resulting in the release of IFN-*γ*, inducible nitric oxide synthase (iNOS), and reactive oxygen species (ROS), and a high level of expression of MHC II molecules^85^. M1-type TAMs exert antitumor effects by releasing these inflammatory mediators to activate a strong T-helper cell 1 (Th1)-type immune response, which promotes an inflammatory response that inhibits cell proliferation and kills pathogens and tumor cells. Unlike M1-type TAMs, M2-type TAMs are less capable of presenting antigens but have anti-inflammatory effects. M2-like macrophages highly express CD163, CD206, CD200R, CD209, CD301 and chemokines such as CCL1, CCL17 and CCL18. They release large amounts of anti-inflammatory factors and express low levels of inflammatory cytokines. M2-type TAMs can function by releasing immunosuppressive chemicals to block Th1-type immunoreactivity and enhance Th2-type immunoreactivity, respectively^86^. This action attenuates the modulation of the inflammatory response while promoting tumor cell proliferation, angiogenesis, and tissue healing, as well as the emergence of drug resistance. Consequently, many studies have been devoted to developing new measures for converting M2 TAMs to M1 TAMs. The characteristics of M1-type polarization include an increase in pro-M1 genes (TNF-*α* and CD86) *in vitro*, a decrease in pro-M2 genes (IL10 and CD206), and upregulation of macrophage CD80 expression *in vivo*. It has been demonstrated that iron oxide nanoparticles can be used for drug delivery and cancer therapy. Ferumoxytol, an iron oxide nanoparticle approved by the FDA for the treatment of anemia, has also been shown to promote macrophage polarization toward the M1 phenotype^87^.

Recently, it has been shown that Cu ions can induce polarization of M1-type TAMs^88^^,^^89^. Studies have shown that the expression of M1-related markers is increased at relatively high concentrations of Cu^2+^ ions (over 100 μmol/L)^90^. Mn-based nanoparticles also promote M1 macrophage polarisation, and some reports suggest that this may be due to the fact that Mn-based nanoparticles enter cells and react with intracellular glutathione to release Mn ions, which generate large amounts of reactive oxygen species through Fenton-like reactions and activate the NF-*κ*B pathway to promote the inflammatory response in macrophages^91^. Iron ions can promote M1-type macrophage polarization^92^^,^^93^, and the advantage of iron metal over other metal ions (*e.g.* Mn ions) that promote TAM reprogramming is that M1-type macrophages are more resistant to iron death. In addition, ferumoxytol, an FDA-approved iron oxide nanoparticle for the treatment of anaemia, has also been shown to promote macrophage polarisation to the M1 phenotype^87^.

### 3.5 Regulation of immune cells by metallic ions

CD8^+^ T cells, as key effector cells of the adaptive immune response, can directly recognize and eliminate tumor cells in an antigen-specific manner^31^^,^^94^. The multistep immune cell-cancer cell and immune cell-immune cell interactions involved in the elimination of tumor cells by CD8^+^ T cells are known as the “cancer–immunity cycle”. In the first step of the cycle, antigens released by cell death are captured by APCs (possibly accompanied by the release of DAMP-based maturation signals). The antigenic epitopes are then further processed by APCs. And present them to the CD8^+^ T cells in the presence of MHC I molecules^95^. The initial T cells receive antigenic signals as well as co-stimulatory signals *via* the T cell receptor (TCR) and the APC and are activated^96^. Activated T cells then migrate and infiltrate the tumor site, recognizing tumor cells by homologous antigens bound to MHC I molecules and exerting cytotoxic activity to induce apoptosis^96^^,^^97^. As a result, tumor cells undergo ICD and dead tumor cells release more tumor cell antigens and DAMP-based maturation signals, further activating the immune cycle^97^. The balance of metal ion metabolism is critical for the differentiation and function of immune cells, especially adaptive immune cells CD8^+^ T cells^98^. Considering that depletion of CD8^+^ T cells within the TME usually leads to low response rates to cancer immunotherapy^90^, modulation of metal ions contributes to CD8^+^ T cell infiltration, thereby reversing local immunosuppression^99^.

It has been shown that elevated extracellular K^+^ impairs TCR-driven Akt-mTOR phosphorylation and promotes the activation of downstream effector signals^99^^,^^100^. Overexpression of the K channel Kv1.3 in a mouse model of melanoma increases K^+^ efflux from specific T cells and enhances the immune response, thereby promoting tumor clearance. The energy to kill tumor cells is restored by removing K^+^ from the T-cells^101^. To summaries, K^+^ can be used as an ionic checkpoint to block T-cell function in cancer immunotherapy Recently, it has been shown that increasing the concentration of K in the TME promotes the persistence and stemness of tumor-infiltrating lymphocytes (TILs) through functional caloric restriction, which promotes autophagy and metabolic reprogramming^47^. In addition, Mg^2+^ can affect T cell function by modulating proximal and distal signaling activities such as focal adhesion kinase (FAK) and extracellular signal-regulated protein kinase 1/2 (ERK1/2) phosphorylation^48^. Furthermore, low levels of Mg^2+^ are associated with inhibiting TCR signaling, which suppresses T-cell proliferation and leads to T-cell failure^99^. It has been shown that an increase in the concentration of Ca^2+^ induces the dissociation of CD3 from the membrane and the exposure of the tyrosine residues to the solvent. This means that Ca^2+^ significantly increases the tyrosine phosphorylation of CD3^102^. Mechanistically, Ca^2+^ influx increases the accessibility of the immunoreceptor tyrosine-based activation motif (ITAM) in the structural domain of CD3 cytoplasm for phosphorylation of the lymphocyte-specific protein tyrosine kinase (Lck), which triggers a signaling cascade to activate T cells^103^.

## 4 Nanocarriers for use in immunotherapy

This is despite the fact that there is a naturally strong association between metal ions and immunity, which can enhance host immunogenicity, increase the rate of immune response, and reduce the incidence of adverse immune events. However, they typically have the disadvantages of poor pharmacokinetics, low target accumulation, off-target reactions, and drug resistance, which limit their efficacy and clinical translation. Because nanoparticles (NPs) can be recognized and internalized by the mononuclear phagocyte system (MPS)^104^, some NPs can take advantage of immune cell uptake to reach and accumulate at the tumor site, thereby overcoming some of the limitations of metalloimmunotherapy. NPs use four main approaches to improve tumor status, including remodeling of physical barriers, co-inhibition and co-stimulation of ligand/receptor targeting, DNA and RNA targeting, and delivery of small molecules that induce cellular stress, immune cell death, and epigenetic modifications^105^^,^^106^. The remodeling of the physical barrier mediated by NPs reduces the deposition of ECM and the occurrence of hypoxic conditions and promotes vascular flow^107^. The co-inhibition and co-stimulation of ligand/receptor targeting mediated by NPs can promote the secretion of cytokines and the reprogramming of TAMs/cancer-associated fibroblasts (CAFs)^108^^,^^109^. Cytokine and co-stimulatory factor signaling can be influenced by NP-mediated DNA and RNA targeting^110^. In addition, small molecules delivered by NPs that induce cellular stress, immune cell death, and epigenetic modifications can promote the release of DAMPs, thereby enhancing organismal immunogenicity. In spite of these multiple functions of NPs, there are a number of limitations to their use in clinical practice. Lipid nanoparticles (LNPs) offer biocompatibility and ease of production but suffer from low drug loading and rapid clearance by the liver^111^. In contrast, polymeric nanoparticles provide controlled release but face aggregation and potential toxicity challenges^112^^,^^113^. Despite their potential, only a few nanocarrier systems have advanced to clinical trials, mainly due to challenges in scaling up production and ensuring consistent patient biodistribution.

Compared to LNPs and polymeric NPs, inorganic NPs can be used to construct nano-delivery systems with high drug loading capacity and stable biodistribution due to their optical properties, tunability, and easy surface modification. In particular, metal–organic frameworks (MOFs), a class of highly ordered porous coordination polymers with high specific surface area, well-defined structure, tunable pore size, and easy chemical modification^114^, ^115^, ^116^, are promising nanodrug carriers^117^, ^118^, ^119^. MOFs are formed with inorganic metal ions/clusters (*e.g.* transition and lanthanide metals) as nodes supported by organic ligands (*e.g.* carboxylates, phosphonates, imidazolates, and phenolates) to form extended and infinite one-/two-/three-dimensional MOF network. In recent years, MOFs carriers have been extensively studied. They can be divided into the following main categories: isoreticular metal–organic frameworks (IRMOFs), zeolite-imidazolate frameworks (ZIFs), materials of institute lavoisier (MILs), pocket-channel frameworks (PCNs)^120^. Metallic nanoparticles used in cancer immunotherapy are summarised in Table 3 121, 122, 123, 124, 125, 126, 127, 128, 129, 130, 131, 132.

**Table 3:** Metallic nanoparticles that have been used in cancer immunotherapy^36^.

Metal	Nanoparticle	Metal material	Delivery technology	Type of disease	Mechanism of immunity	Ref.
Mn	PL/APMP-DOX	Mn_3_ (PO_4_)_2_	Lipid bilayer coating	Breast cancer (4T1 cells)	Augmenting cGAS–STING activity	121
	H–MnO_2_-PEG/C&D	Hollow MnO_2_	Polyethylene-glycol (PEG)	Breast cancer (4T1 cells)	Enhancing macrophages infiltration; facilitating M2/M1 polarization	122
	CpG@PLGA-PLLmPEG/SPIO	Superparamagnetic iron oxide (SPIO)	mPEG-PLGA-PLL triblock copolymer	Breast cancer (4T1 cells)	Activating DCs; inducing TNF-*α*, IL-6 and IFN-*γ* secretion	123
Fe	PEG-coated ferrihydrite nanoparticles (PEG-Fns)	Fe(NO_3_)_3_·H_2_O	Biocompatible PEG-coating	Lung cancer (SCC-7 cells)	Inducing M1 macrophage polarization	124
	Fe^2+^/siRNA/PDA nanoparticles (FesiRNAPNPs)	FeCl_2_	Polydopamine (PDA)	Colon cancer (CT26 cells)	ROS generation & GSH consumption in the TME leads to tumor-killing	125
Au	PolyA-CpG-AuNPs	Nano-gold (AuNPs)	Poly-adenine (polyA) tail	Leukemia (RAW264.7)	Immune-stimulatory activity *via* TLR9	126
	Mangiferin functionalized (MGF)-AuNPs	NaAuCl_4_	Mangiferin (polyphenol: Dglucoside)	Prostate cancer	Antitumor cytokines (IL-12 and TNF-*α*) ↑;	127
Zn	Zn^2+^@ SA/PCT	ZnCl_2_	Hesperidin	Colon cancer (HCT116)	Inducing apoptosis *via* excessive generation of ROS	128
	ZnS@BSA	ZnS	BSA (bovine serum albumin)	Hepatoma	Activating the cGAS–STING pathway	129
Cu	CuS-sorafenib-anti-VEGFR antibodies (CuS-SF@CMV)	Hollow-CuS	Cancer cell-macrophage hybrid membrane-coating	Hepatocellular carcinoma (HepG2 cells)	Localizing to the homotypic cells and escapes immune cell-mediated elimination	130
	AM@DLMSN@ CuS/R848	Cu sulfide (CuS)	Dendritic large-pore mesoporous silica nanoparticles (DLMSNs)	Breast cancer (4T1 cells)	Photothermal ablation-induced ICD	131
Ag	Silver/silver sulfide janus nanoparticle (Ag/Ag_2_S JNP)	AgNO_3_	PEGylated	Breast cancer (MCF7)	Photothermal therapy (PTT); noninvasive location and diagnosis *in vivo*	132

DC, dendritic cell; TNF-*α*, tumor necrosis factor-*α*; IL-6, interleukin-6; IFN-*γ*, interferon gamma; ROS, reactive oxygen species; GSH, glutathione, R-glutamyl cysteingl + glycine; TME, tumor micro-environment; NPs, nanoparticles; TLR9, Toll-like receptor 9; IL-12, interleukin-12; ICD, immunogenic cell death.

IRMOFs, as a common MOF material, are mainly formed by [Zn_4_O_6_]^+^ metal clusters bonded with carboxylic acid-based organic ligands to form a repetitive network topology with high pore volume and pore cavities, and their specific surface area can be as high as ∼2900 m^2^/g^133^. ZIF materials are self-assembled from Zn or Co with N above the imidazole (or imidazole derivative) ring in a tetra-coordinated manner^134^. Compared to other MOFs, ZIF materials have better thermal and chemical stability. They are also easy to functionalize. ZIF-90 in ZIF can deliver the genome editing protein Cas9, and the genome editing protein Cas9 can effectively inhibit the expression of green fluorescent protein (GFP) in HeLa cells with an efficiency of up to 35%^135^. Another more specific type of MOFs material, MILs, is made by using trivalent transition metal ions (*e.g.*, Fe, Al, and Cr) coordinated with carboxylic acid-based ligands (terephthalic acid, homobenzoic acid). MIL-100 can be used for loading ICG^136^, topotecan^137^, brimonidine tartrate^138^, and DOX^139^. In addition, there is a class of MOF materials known as PCNs. The classic structure among the PCNs is HKUST-1 (CuBTC), which uses Cu_2_(COO)_4_ clusters that are coordinated with homobenzoic acid and have a two-pore structure^140^.

## 5 Metallic nanoparticles for cancer immunotherapy

### 5.1 Mn-based nanoparticles for immunotherapeutic applications

Mn is an essential trace element, second only to iron, in the human body and has a variety of physiological functions, such as maintaining energy metabolism, regulating the immune system, and improving hematopoiesis^141^^,^^142^. Mn also has many applications in nanomedicine based on its multiple physiological functions, including as a delivery vehicle for cancer immunotherapy, an immunomodulator to regulate the TME, an immune activator to activate the immune response, and magnetic resonance imaging (MRI) imaging capabilities. Precisely because of the above applications of elemental Mn in nanomedicine, Mn holds great promise for constituting a multifunctional nanomedicine platform. The following section briefly discusses the basic properties and functions of Mn. Based on this, the discussion continues with the design of Mn-based nanoplatforms.

Mn is important in glucose, lipid, and amino acid metabolism, as well as cellular energy regulation. Moreover, Mn is an essential component of arginine kinase and pyruvate carboxylase and an activator of kinases, transferases, and hydrolases^143^^,^^144^. There is also a Mn-related enzyme known as Mn superoxide dismutase (MnSOD). MnSOD, an antioxidant metalloenzyme, catalyzes the disproportionation of superoxide anion radicals to generate oxygen and hydrogen peroxide, which plays a crucial role in the oxidative and antioxidant balance of the organism^145^. MnSOD is also one of the most important antioxidant enzymes in mitochondria, playing an important role in controlling T-cell activation and inducing oxidative signaling^146^. Additionally, Mn activates the immune response. One of the pathways that activates the immune response is the cGAS–STING pathway^37^^,^^55^. It has been shown that Mn^2+^ can increase the sensitivity of cGAS for DNA detection, promote the synthesis of the second messenger cGAMP by a factor of 10,000, and enhance the binding ability of STING to cGAMP by a factor of 100^56^. In 2020, Qi et al.^10^ further elucidated the molecular mechanism of cGAS activation by Mn^2+^ and found that Mn^2+^ can also directly activate cGAS (utterly independent of dsDNA) and that Mn^2+^ catalyzes the synthesis of cGAMP by cGAS catalytically different from that of dsDNA-bound cGAS catalytically by Mg^2+^. In this way, Mn^2+^ released into the cytoplasm places the cGAS–STING pathway in a state of hyperactivation, greatly enhancing its ability to respond to cytoplasmic DNA, even at levels of DNA that would otherwise be inactive. In addition, Mn-based nanomaterials can be used to modulate the TME by alleviating TME hypoxia, promoting tumor cell reprogramming, and combining with immune checkpoints based on the Fenton-like response of Mn ions, pro-M1 macrophage polarisation and “Mn immunotherapy”^10^. Chemodynamic therapy (CDT) is an essential strategy for overcoming TME immunosuppression by promoting the Fenton response. For example, in an acidic TME, MnO_2_ nanoparticles can be reduced to Mn^2+^ by endogenous overexpression of H_2_O_2_ in solid tumors and generate large amounts of oxygen to overcome tumor hypoxia^147^. Several studies have also shown that Mn-based nanoparticles can create a cascade of adaptive immune responses to alleviate the immunosuppressive nature of the TME by reducing Tregs and polarizing M2-type macrophages to the M1-type^148^^,^^149^. Mn^2+^, a paramagnetic ion, is characterized by a high spin number, a long electronic relaxation time, and labile water exchange. Based on these properties, Mn^2+^ increases the signal intensity of T1-weighted MRI, resulting in brighter images with significant positive contrast enhancement^150^, ^151^, ^152^. Notably, Mn^2+^ can significantly increase relaxivity and enhance MRI contrast signals after binding to proteins^153^^,^^154^.

In summary, due to the immunomodulatory function of Mn, Mn-based nanoparticles can directly activate the immune response and further enhance the immunotherapeutic effect by modulating the TME. Moreover, Mn-based nanoparticles also have an MRI function due to the high spin number and labile water exchange of Mn, which can be used to monitor the process of tumor treatment and evaluate the effect of tumor treatment. Therefore, Mn-based nanomaterials can be used to construct multifunctional nanoplatforms for cancer immunotherapy Compared to other metals used in immunotherapy, such as Cu, Fe, Zn, and Au, Mn is unique in that it has multiple functions. Notably, many metal ions generally have unsatisfactory antitumor effects *in vivo* due to their low safe concentrations in the human body. However, Mn ions can act as immunotherapeutic agents through a variety of pathways, including activation of the immune response through the cGAS–STING pathway, initiation of the Toll-like receptor-4 (TLR4)-associated signaling pathway to reverse the immunosuppressive microenvironment of the tumor, and induction of ICD through CDT.

#### 5.1.1 Mn-based nanoparticles as efficient nanocarriers for targeted immunotherapy

As immunotherapeutic agents, specific antibodies, genes, and cytokines are susceptible to enzymatic degradation and loss of activity in the human body. Therefore, delivery vehicles must efficiently deliver the active substances to the target site. Nanomaterials are often used as drug delivery carriers owing to their small particle size, biocompatibility, and targeted modification^155^. When the size of MOF particles is reduced to the nanoscale, these nano-MOFs (NMOFs) can be used as drug delivery carriers for imaging, chemotherapy, PTT, or PDT. Drugs, such as nucleic acids, proteins, and other active substances, can be loaded into MOF by *in situ* encapsulation or synthesis followed by modification. Compared to other metallic nanoparticles, Mn-based nanoparticles as nanocarriers not only have better biocompatibility and stability but also interact with the tumor microenvironment^156^.

**Figure 4:**
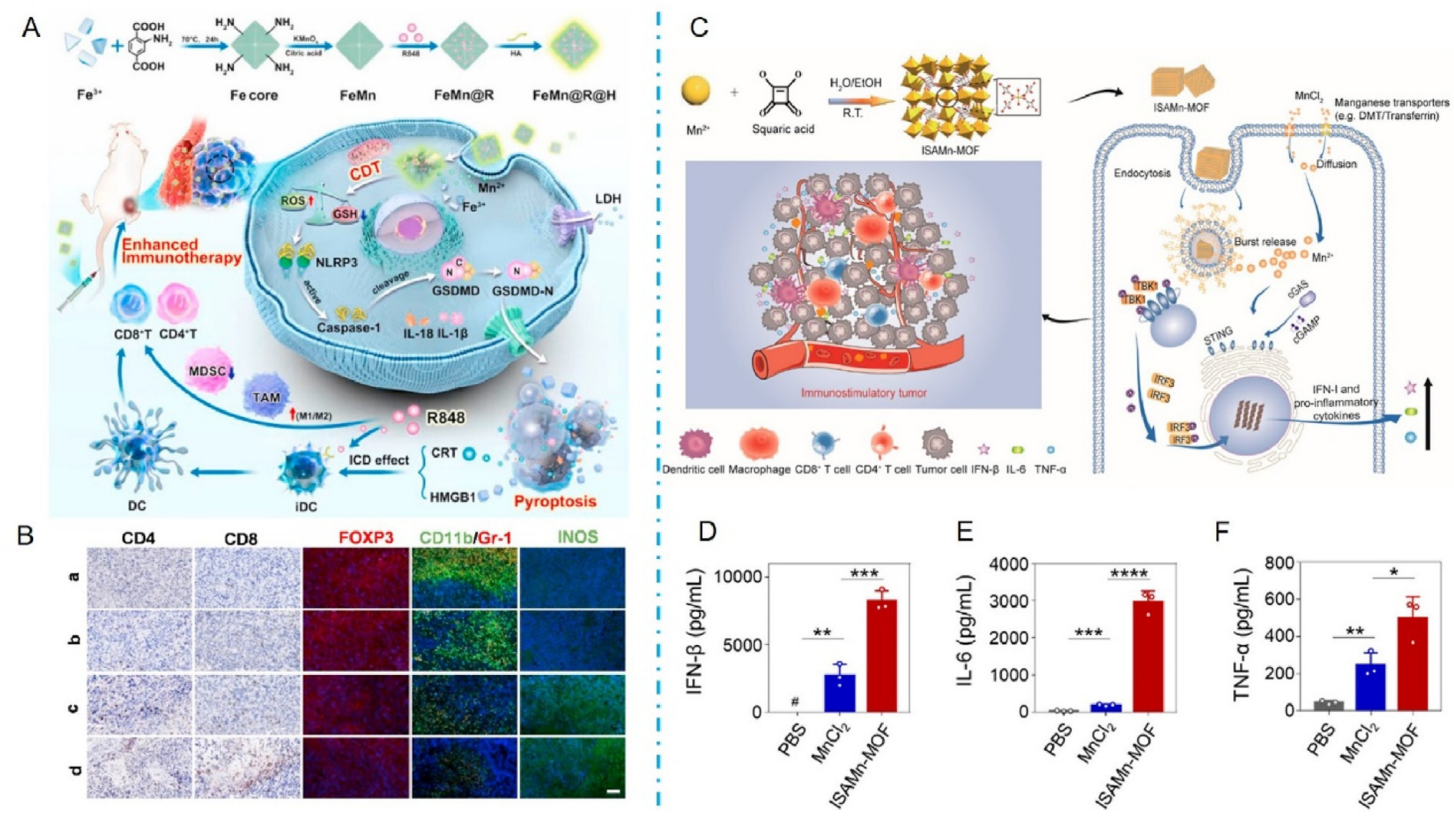
Mn-based nanoparticles as nano delivery carriers for immunotherapy. (A) Schematic illustration of synthesis tactics and molecular mechanism of FeMn@R@H against tumors through pyroptosis and immune regulation. (B) Representative immunohistochemical staining images of CD4 T cells, CD8 T cells, and immunofluorescence images of Treg (FOXP3, red), MDSCs (Gr-1/CD11b, green/red), and M1 (INOS, green) in tumors collected from different groups after treatment. Scale bar:150 μm^157^. Copyright 2023, Elsevier. (C) Schematic diagram of the synthesis and working mechanism of Mn-based immunostimulation of MOFs. (D) Detection of IFN-*β* in bone marrow-derived dendritic cell (BMDC) supernatants by ELISA. (E) IL-6 was detected in BMDC supernatants by ELISA. (F) Detection of TNF-*α* in BMDC supernatants by ELISA^158^. Reprinted with the permission from Ref. 158. Copyright 2023, American Chemical Society.

For example, Feng et al.^157^ designed an acid-responsive iron/Mn bimetallic organ framework nanosystem (FeMn@R@H) loaded with metal ions and the immunoadjuvant R848 and used it for cellular targeting and immunotherapy (Fig. 4A). This study focused on the loading of active compounds within the hollow structure of a bimetallic organoskeleton and encapsulation with hyaluronic acid. The FeMn@R@H system is noteworthy due to its dual functionality in delivering both metal ions and immunoadjuvants, offering a more comprehensive approach to modulating the immune response. MOFs serve as delivery vehicles and can be rapidly degraded to Fe^3+^ and Mn^2+^ upon accumulation at the tumor site, initiating a Fenton-like reaction for ROS-mediated pyroptosis. The results showed increased infiltration of tumor-suppressing cytotoxic T cells (CD4 and CD8 T cells) in the FeMn@R@H group (Fig. 4B). In addition, most MOFs are biologically inert carriers, and creating MOFs with immunostimulatory properties remains challenging. Zheng et al.^158^ used a simple and environmentally friendly method to prepare Mn^2+^-based immunostimulatory MOF (ISAMn-MOF) (Fig. 4C). They did not rely on similar synthesis conditions, such as high temperatures and organic solvents. Still, they used MnCl_2_ and the sodium salt of squaric acid (SA) as raw materials, with simple stirring at room temperature for 10 min. Remarkably, in this study, ISAMn-MOF acted as a delivery vehicle and achieved immune activation. ISAMn-MOF significantly promotes the activation of cyclic GMP–AMP synthase-stimulating factor-related genes and interferon signaling pathways in bone marrow-derived dendritic cells (BMDCs). BMDCs treated with ISAMn-MOF secreted 4-fold more IFN and 2- to 16-fold more pro-inflammatory cytokines than those treated with equivalent amounts of MnCl_2_ (Fig. 4D–F2). In addition, there is a fascinating study. In this study, an Mn^3+^-containing precursor (Mn (III) acetate dihydrate) was utilized for the first time to replace traditional Mn^2+^ species and introduce additional MnIV–O bonds in MOF. This is due to the partial and rapid disproportionation of Mn^2+^ and Mn^4+^^159^. The Mn–O bond, which has both mixed high-valence and coordination-unsaturated properties, serves as the catalytic activity center of this nano-enzyme with high activity, and its excellent substrate affinity and flexible molecular conformation are the key structural factors determining the aptameric nano-enzymes it serves as, which provides an essential theoretical model for the construction of artificial aptameric enzymes. In addition, the ultrasmall Mn-based MOF (nMnBTC) cold-adapted nano-enzymes creatively constructed in this study using MOFs as a platform not only have excellent enzyme catalytic activity in the low-temperature region of 0 °C but also the catalytic activity of the enzyme is almost unchanged when the temperature increases to the medium-temperature region (45 °C). This phenomenon breaks the conventional knowledge that natural and traditional artificial enzymes exhibit optimal activity only at specific temperature zones.

In summary, Mn-based nanomaterials are used as nanocarriers in immunotherapy, and researchers often use MOFs as a platform to design nano-delivery systems, which can not only be used as carriers to deliver drugs but also have the potential to activate immune responses. Researchers have also exploited Mn metal elements' and MOF carriers’ properties to create novel structures to break traditional perceptions.

#### 5.1.2 Modulation of the tumor immune microenvironment as an adjuvant to enhance immunotherapy

Although immunotherapy has achieved excellent clinical success^160^^,^^161^, its therapeutic efficacy is primarily limited by insufficient immune activation and an immunosuppressive TME^162^, ^163^, ^164^. TME refers to the internal environment in which the tumor resides, which is mainly composed of components such as tumor cells, immune cells and their secreted factors, stromal cells, and vascular endothelial cells, and is the soil on which the tumor feeds^165^. The exuberant metabolic demands of tumor cells lead to hypoxia, microacidity, and high ROS levels within the tumor. These properties affect the activity of pro-inflammatory cells, which in turn leads to immunosuppression^166^.

Hypoxia is a widespread trait in 90% of solid tumors and plays an essential role in the immunosuppressive TME by promoting accelerated tumor cell multiplication, proliferation, metastasis, and recurrence^167^^,^^168^. Tumor cells tend to use the glycolytic pathway for energy provision as they develop rapidly, and the complexity of the intermediates can meet the needs of the rapid proliferation of tumor cells. However, glycolysis can also result in the accumulation of lactic acid, which provides the conditions for an acidic and hypoxic microenvironment in tumor cells. This acidic hypoxic microenvironment can activate the upregulation of hypoxia-inducible factors (HIFs) and reduce or even shut down oxygen-consuming processes in the mitochondria, primarily oxidative phosphorylation, by acting as a transcriptional regulator, thus making glycolysis the primary mode of energy production in cancer cells^169^. This further exacerbates hypoxia, creating a vicious cycle that promotes tumor progression. Hence, hypoxia plays an essential role in the immunosuppression of the TME. As Mn has multiple valence states, Mn-based nanomaterials are expected to modulate TME through redox reactions, in particular, MnOx-containing nanoparticles are expected to ameliorate immunosuppressive TME by generating oxygen through overexpression of H_2_O_2_ within the tumor to alleviate tumor hypoxia. The Fenton-like reaction catalyzed by MnO_2_ generates reactive oxygen species (ROS), which decompose excess H_2_O_2_ in the TME, thereby alleviating hypoxia and enhancing the efficacy of oxygen-dependent therapies. In addition, MnO_2_ can undergo a redox reaction *via* intracellular overexpression of GSH to generate Mn^2+^ with glutathione disulphide (GSSG), which significantly reduces the level of antioxidant GSH in tumor cells and consequently increases the sensitivity of tumor cells to ROS. Thus, Mn-based nanomaterials can modulate TME through redox reactions and are particularly suitable for the treatment of ROS/oxygen-dependent tumors.

**Figure 5:**
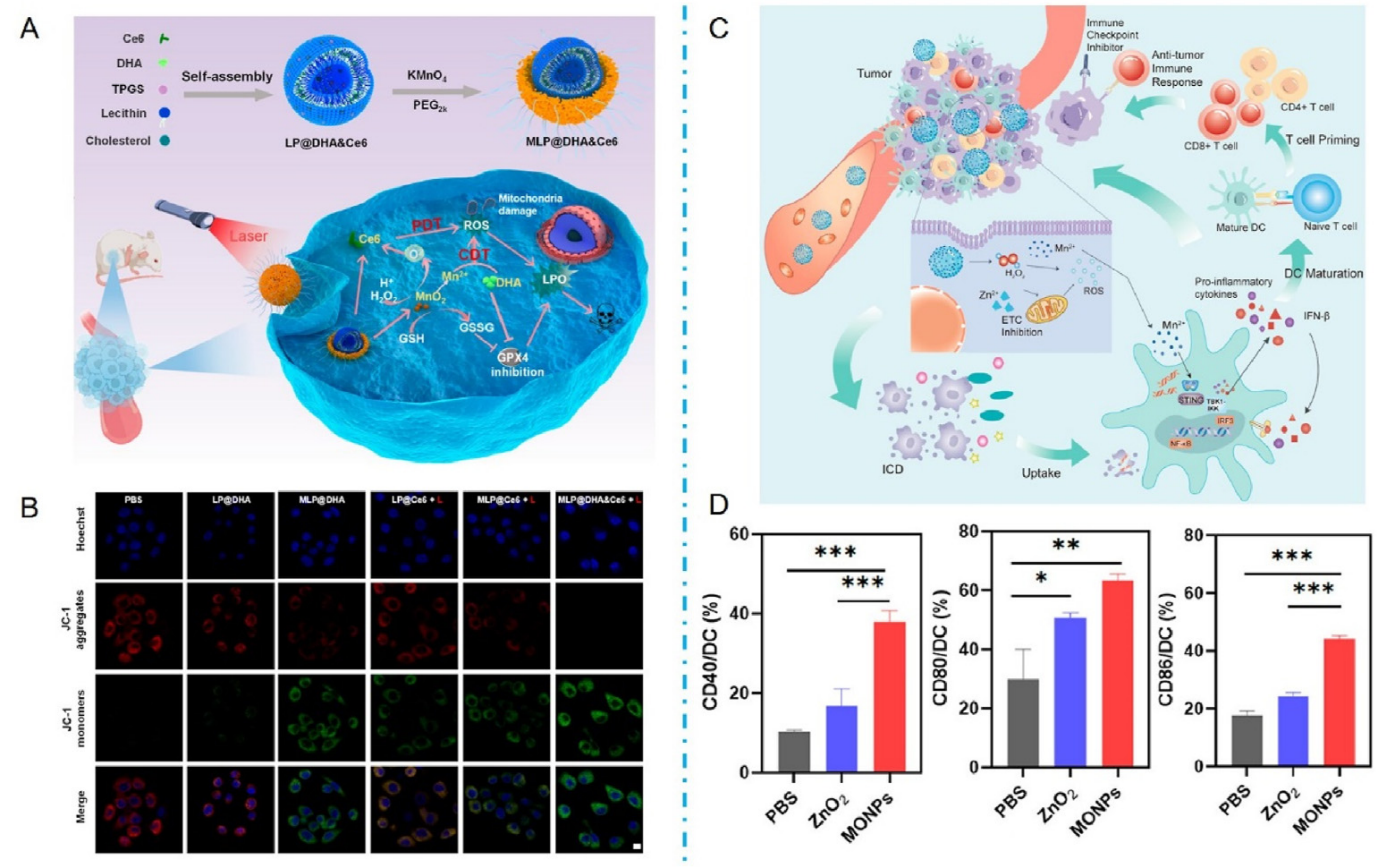
Mn for immunotherapy by modulating the tumor microenvironment. (A) Multiple strategies of nano enzymes to induce ROS generation to alleviate tumor hypoxia. (B) MMP of BxPC-3 cells was measured by JC-1 (LP@Ce6 40 μg/mL, LP@DHA 40 μg/mL, MLP@Ce6 200 μg/mL, MLP@DHA 200 μg/mL, and MLP@DHA&Ce6 200 μg/mL). Scale bar: 10 μm^172^. Reprinted with the permission from Ref. 172. Copyright 2023, American Chemical Society. (C) Mn perovskite functional nanoparticles for enhancing cancer immunotherapy. (D) The expression of costimulatory molecules CD40, CD80, and CD86 on BMDCs^148^. Reprinted with the permission from Ref. 148. Copyright 2023, American Chemical Society.

For example, the Jun Gui and Yu Yang team from Shanghai Jiaotong University jointly constructed a Mn-based single-atom nano-enzyme Mn–N/C with ZIF-8 as the backbone^170^. The nano-enzymes converted intracellular H_2_O_2_ to ·OH *via* the Fenton reaction. They complemented the generation of ROS, which alleviated the hypoxic state of the TME while inducing ICD and promoting the anti-tumor immunity of CD8^+^ T cells. ZIF-8 is also a type of MOF structure with adjustable channels and a large specific surface area, so it has a tremendous advantage in the adsorption and loading of drugs. Prof. Yangzhong Liu and Prof. Yuen Wu at CUHK constructed a PEGylated Mn-based single-atom nano-enzyme (Mn/PSAE) based on the coordination of single-atom Mn with nitrogen atoms based on a hollow ZIF^171^. Nano-enzymes can also catalyze the conversion of intracellular H_2_O_2_ to ·OH *via* the Fenton reaction and promote the decomposition of H_2_O_2_ to produce O_2_, thereby alleviating the hypoxic state of tumors. In addition, the enzyme induces apoptosis in tumor cells by transferring electrons to O_2_ through an oxidase-like activity, generating large amounts of cytotoxic superoxide radicals·O^2−^. In addition to the Fenton reaction to replenish ROS, TME anoxia can be alleviated by a Mn redox reaction. For example, Liu et al.^172^ constructed a TME-responsive nano-enzyme by co-encapsulating dihydroartemisinin (DHA) and the photosensitizer Ce6 in lipid-supported Mn oxide nanoparticles (Fig. 5A). Nano-enzymes can generate ROS from multiple sources and significantly improve tumor hypoxia while overcoming the limitations of CDT and PDT in the TME. The nano-enzymes accumulate at the tumor site, and their loaded MnO_2_ reacts with *in situ* H_2_O_2_ in the tumor tissue to release Mn^2+^ and O_2_. The released O_2_ can increase the PDT efficiency, and the released Mn^2+^ can further catalyze the Fenton reaction, resulting in more ROS (Fig. 5B). In addition, the use of MnOx nanoparticles in combination with photothermal therapy resulted in an 80% reduction in tumor size compared to controls, demonstrating the potential of these nanoparticles to modulate the TME effectively. Therefore, Mn-based nanomaterials have great potential for alleviating tumor hypoxia.

**Figure 6:**
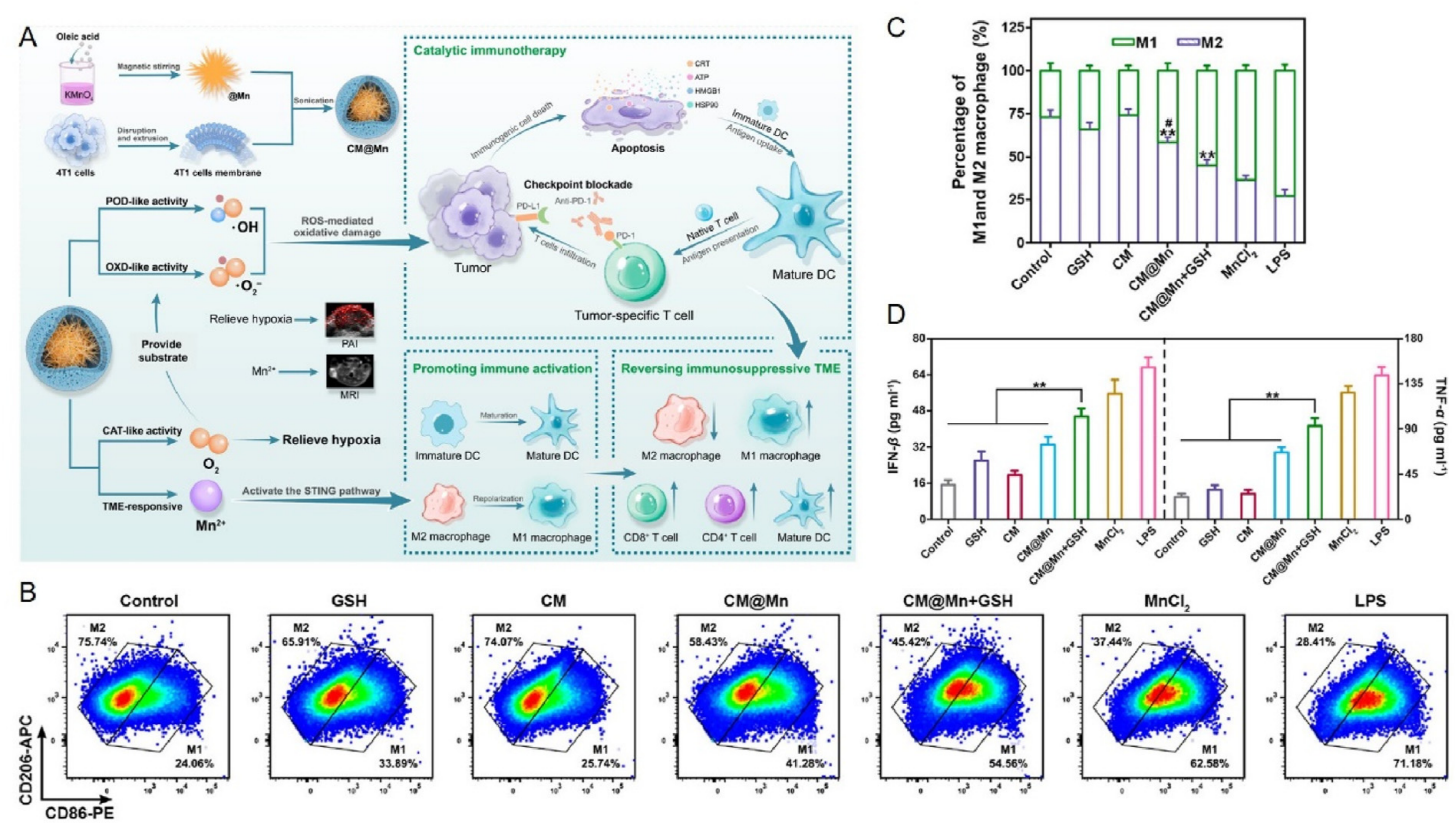
Mn modulates immunotherapy by activating the tumor microenvironment. (A) Mn nanoenzymes therapeutic strategy and schematic diagram. (B, C) Representative images of BMDMs (M1 macrophage: F4/80 CD11c^###+–^ CD86^high^ CD206^low^, M2 macrophage: F4/80 CD11c^+ –^ CD86^low^ CD206^high^, gated on F4/80 CD11c cell) analyzed by flow cytometry and corresponding quantitative analysis (C) after treatment with different conditions for 24 h. (D) Quantification of the cytokine IFN-*β* ecreted by BMDC^173^. Reprinted with the permission from Ref. 173. Copyright 2022, American Chemical Society.

Mn-based nanoparticles can also polarize M1-type macrophages. It has been suggested that the mechanism may be that Mn-based nanoparticles enter the cell and react with intracellular glutathione to release Mn ions, which generate large amounts of reactive oxygen species through the Fenton-like reaction and activate the NF-*κ*B pathway to promote inflammatory responses in macrophages^91^. Some studies have constructed Mn-rich Zn peroxide nanoparticles to generate a cascading adaptive immune response and alleviate immunosuppression by decreasing Tregs and polarizing M2-type macrophages to M1-type macrophages (Fig. 5C)^148^. The study results showed a 3.03-fold reduction in Tregs in the nanoparticle group compared with the other groups. In addition, this nanoparticle group's M1/M2 ratio increased by 3.82–2.78 times compared to the other groups. Furthermore, the nanoparticles segregated upon exposure to acidic tumor tissue, resulting in the *in situ* production of ·OH to induce the ICD effect (Fig. 5D). There have also been studies that combined PTT with Mn-based nanomaterials. For example, Li et al.^149^ constructed thermoresponsive micelles by co-encapsulating IR780 dye and Mn–Zn sulfide nanoparticles (ZMS) in amphiphilic poly (ethylene glycol)-poly (2-hexyloxy-2-oxo-1,3,2-dioxaphosphorane) copolymers (mPEG-b-PHEP) with a thermosensitive flowable core. These micelles can precisely control the release of ZMS under near-infrared light (NIR) triggering cancer metal immunotherapy. Photothermally triggered Mn^2+^-mediated CDT promoted ROS production, and the results showed that the micelles maximized ICD efficiency in cancer cells, allowing exposure to many damage-associated molecular patterns (DAMP). In addition, nano micelles further reprogrammed the immunosuppressive TME by promoting the infiltration of CD8^+^ and CD4^+^ into tumor lesions. In addition to the above strategies to alleviate the immunosuppressive TME, Zhao et al.^173^ combined a multi-enzyme mimetic Mn oxide nano-enzyme encapsulating the tumor cell membrane (CM) with a PD-1 monoclonal antibody (*α*PD-1) to construct a TME-activated, Mn-enhanced catalytic immunotherapy and synergized it with ICB therapy to kill tumors effectively (Fig. 6A). The nanoenzymes have multiple mimetic enzyme activities. They can exhibit peroxidase-like and oxidase-like activities in an acidic TME, which in turn can ·OH and O^2−^ to kill tumor cells and trigger ICD. Remarkably, it was shown that Mn^2+^ released by the nano-enzyme in response to TME was able to directly promote DC and macrophage M1 polarization, leading to the reversal of an immunosuppressive TME into an immunologically activated environment (Fig. 6B and C). Thus, the nano-enzymes could further reverse the TME in alleviating tumor hypoxia and pro-tumor cell reprogramming. In addition, experimental results showed that the combination with PD-1 checkpoint blockade to induce tumor-specific T-cell-mediated anti-tumor responses effectively inhibited primary and metastatic tumor growth and increased the release of both IFN-*β* and TNF-*α* (an important indicator of anti-tumor cellular immunity and direct tumor cell killing), suggesting a long-term immune memory effect (Fig. 6D). In summary, this study provides a new and effective strategy for combined tumor therapy. Therefore, Mn-based nanomaterials have great potential for application in pro-tumor cell reprogramming.

There is also a class of inhibitory signaling pathways in the TME known as immune checkpoints. Immune checkpoints are small molecules expressed on immune cells that regulate the degree of immune activation and prevent autoimmune effects^174^. In Layman's terms, these are small molecules of proteins produced by immune cells that regulate autoimmune functions. PD-1 has been an essential harmful co-stimulatory molecule since the discovery of CTLA4. PD-1 binds to its ligand (PD-L1) and transmits negative co-stimulatory signals that inhibit the proliferation of T lymphocytes, which play a crucial role in regulating T-cell activation and immune tolerance and are essential in preventing the onset and progression of autoimmune diseases^175^. However, PD-1 plays a negative role in anti-viral and anti-tumor activities. PD-L1 is also expressed on the surface of many tumor cells and binds to PD-1 on the surface of the corresponding lymphocytes, inhibiting lymphocyte function and cytokine release, and inducing lymphocyte apoptosis, leading to immune escape of tumor cells. Thus, blocking the PD-1/PD-L1 pathway can enhance lymphocyte activity in cancer immunotherapy The combination of Mn^2+^ and PD-1 antibodies (“Mn immunotherapy”) has been used and it has been found that Mn immunotherapy significantly enhances the efficacy of PD-1 antibodies and reduces their dosage in a variety of tumor models^10^. In addition, phase I clinical results showed impressive and significant efficacy of Mn immunotherapy against a wide range of relapsed refractory or progressive tumors of epithelial origin (objective remission and disease control rates of 45.5% and 90.9%, respectively). Surprisingly, five PD-1 inhibitor-resistant patients achieved better disease control after treatment with Mn immunotherapy, and three achieved partial remission. This suggests a potential regulatory role for Mn^2+^ in immune checkpoints. Therefore, researchers have devised strategies for using Mn^2+^ with immune checkpoints. For example, the Mn-rich zinc peroxide nanoparticles exemplified above can not only promote tumor cell reprogramming but also inhibit tumor growth and prevent lung metastasis in combination with anti-PD-1 antibodies^148^. In addition, the tumor cell membrane-encapsulated Mn oxide (MnOx) nano-enzymes with multiple mimetic enzyme activities reviewed above effectively inhibited primary and metastatic tumor growth in combination with the PD-1 checkpoint blockade^173^. In addition, Liu et al.^176^ from Shanghai Jiaotong University combined chemotherapy and PDT based on metal ion-drug core–shell nanoparticles to achieve enhanced antitumor effects of *α*-PD1. The core and shell of this nanoparticle were formed by self-assembly of Mn^2+^ and the chemotherapeutic drug adriamycin and photosensitizer Ce6, respectively. As the drug is assembled as a bridging ligand, the overall drug load can be as high as 90%. To reduce the removal of particles by the lymphatic system, nanoparticles can also encapsulate the erythrocyte membrane to achieve prolonged *in vivo* circulation and increase *in vivo* biocompatibility. Therefore, after systemic administration of highly drug-loaded and biocompatible nanoparticles, they can both inhibit the proliferation of tumor cells and promote the infiltration of T-lymphocytes, which significantly enhances the anti-tumor effect of anti-*α*-PD1.

In summary, Mn-based nanomedicines can modulate TME in various ways. First, oxygen can be supplemented by the Fenton and Mn redox reactions to relieve tumor hypoxia. Second, it promotes antigen presentation by facilitating the conversion of M2-type TAMs to M1-type TAMs, thereby inducing an anti-tumor immune response. Finally, it can be combined with immune checkpoints to inhibit primary tumor growth and trigger long-term anti-tumor immune memory. While Mn-based nanoparticles show promise in reprogramming the TME, there is a risk of off-target effects or unintended activation of immune cells, which could lead to adverse inflammatory responses.

#### 5.1.3 Mn-based nanoparticles as immune activators for the direct or synergistic activation of the immune response

Mn^2+^ increases sensitivity to DNA and the enzymatic activity of cGAS and promotes the binding of cGAMP to STING, thereby enhancing the action of multiple factors in the cGAS–STING pathway. In addition, Mn^2+^ promotes the presentation of tumor-specific antigens by facilitating the maturation of DCs with macrophages, thereby enhancing the differentiation and activation of CD8^+^ T cells and NK cells^10^^,^^177^. For example, PEGylated Mn-based metal–organic frameworks can activate innate immune therapeutic tumors by increasing the number of DCs in the TME, thus demonstrating efficient therapeutic effects in pancreatic tumors^178^. James J. Moon et al.^37^ at the University of Michigan designed metal immunotherapeutic nanoparticles based on STING agonist CDN stimulators and Mn^2+^. It was demonstrated that Mn^2+^ promotes self-assembly with STING agonists to form nanoparticles (CDN-Mn^2+^ particles, CMPs) and that CMPs significantly increase cellular uptake, STING activation, and *in vitro* IFN-*β* response to CDN. Notably, other STING agonists often use intratumoral (i.t.) administration as the route of administration; however, they are not indicated for the treatment of metastases. In addition, CDN-based STING agonists for i.t. injections have produced disappointing results in clinical trials. However, CMP in this study can exert significant antitumor efficacy by significantly increasing STING activation and reversing immunosuppressive TME *via* the i.t. or intravenous (i.v.) pathways. The results showed that, in head and neck cancer tumors, intravenous injection of CMP eliminated 75% of the tumors in mice. This work demonstrates for the first time the strong potential of nanomedicine-based metal immunotherapy for cancer and proposes the concept of “metal immunotherapy”.

**Figure 7:**
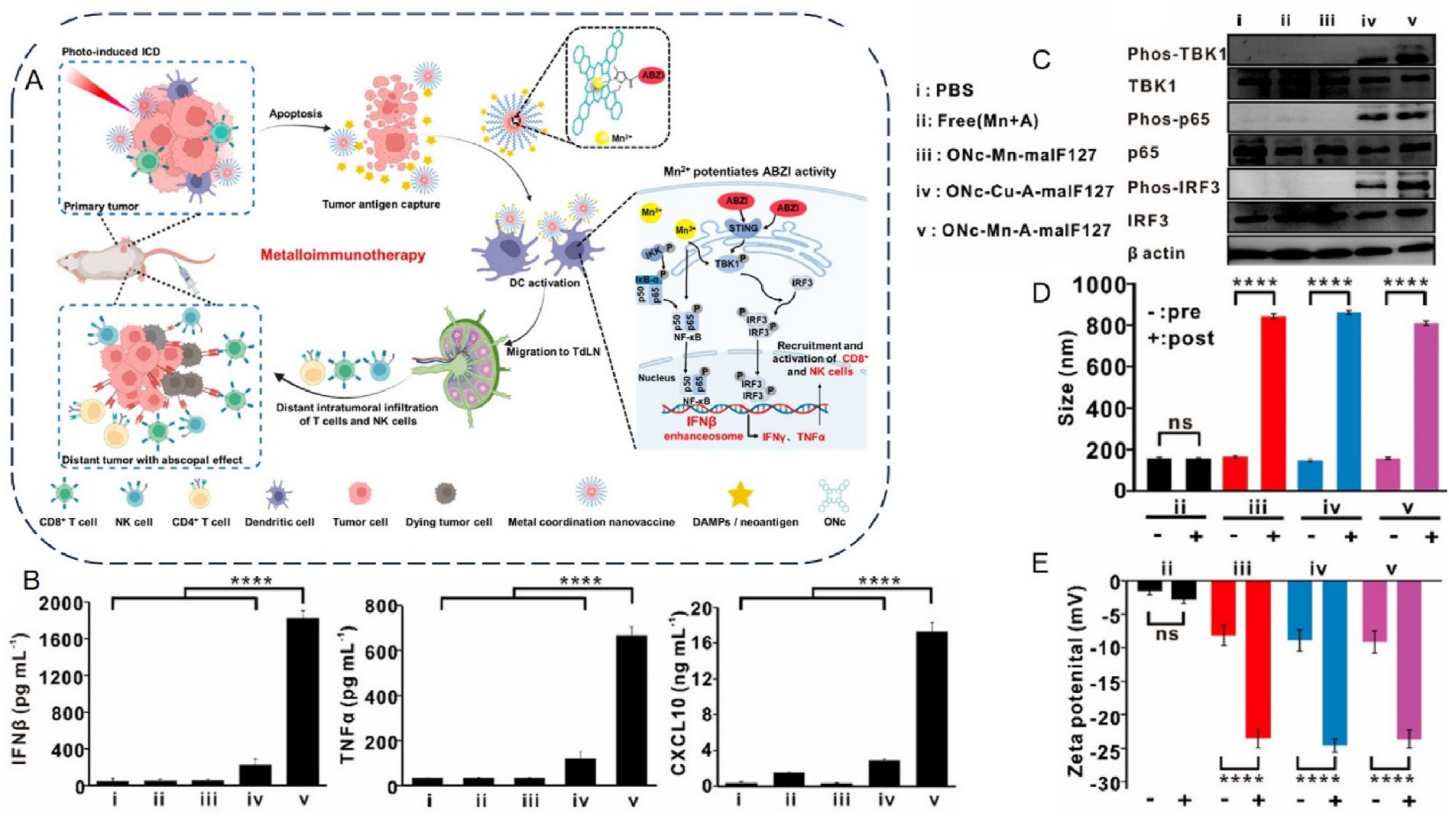
ONc-Mn-A-malF127 is used for metal immunotherapy by activating the cGAS–STING pathway. (A) Schematic representation of ONc-Mn-A-malF127 for use in immunotherapy. (B) Amount of change in serum IFN-*β*, TNF-*α*, and CXCL10 after intravenous injection of ONc-Mn-A-malF127. (C) Western blot analysis of the activation of the cGAS–STING–IFN-I pathway in BMDCs after treatment with the different samples containing 2 μmol/L ABZI and 15 μmol/L Mn^2+^ for 6 h. (D) Size of ONc-Mn-A-F127, ONc-Mn-malF127, ONc-Cu-A-malF127, or ONc-Mn-A-malF127 before and after antigen capture. (E) Zeta potential of ONc-Mn-A-F127, ONc-Mn-malF127, ONc-Cu-A-malF127, or ONc-Mn-A-malF127 before and after antigen capture^181^. Reprinted with the permission from Ref. 181. Copyright 2022, American Chemical Society.

Mn also acts as a potent adjuvant, increasing the affinity of cGAS for double-stranded DNA, promoting the activation of the STING cascade, and activating the cGAS–STING pathway in APCs, which in turn enhances tumor-specific T-cell responses and increases the production of pro-inflammatory cytokines and chemokines^37^^,^^55^^,^^179^. Jiang's group^179^ at Peking University has designed a Mn nano-adjuvant (MnJ) that acts as both a delivery system and an immune activator. This innovative adjuvant is highly effective in activating cellular immunity and promoting humoral immunity. It can also be used as a mucosal immune adjuvant to activate mucosal immune responses and induce secretory IgA production when administered intranasally. The study results showed that the Mn nano-adjuvant was effective against almost all types of antigens tested, significantly improving the effectiveness of the inactivated vaccine, even reducing the concentration of inactivated viruses to 1/100th of their concentration and still achieving full protection. Moreover, the tumor vaccine prepared with Mn adjuvant could significantly inhibit tumor growth and metastasis by promoting the activation of NK cells and infiltration of CD8^+^ T cells. In addition, Mn adjuvants showed a more significant adjuvant effect than other adjuvants, with the possible molecular basis being that Mn^2+^ not only induces the production of *β* interferons (*e.g.*, LPS induces only *β* interferons) but also a large number of various *α* interferons. The large amount of interferon produced by induction significantly promotes the maturation, differentiation, and delivery of antigens to APCs. Mn can also co-activate the cGAS–STING pathway along with other immune activators. A study constructed an Mn-based metal–organic framework loaded with heavy building saponin I (PPI) and encapsulated with erythrocyte membranes to enhance cGAS–STING mediated anti-tumor immunity^180^. It has been shown that PPIs can, on the one hand, differentiate macrophage phenotypes *via* STING to attenuate tumor-induced immunosuppression in lung cancer. In contrast, PPIs can inhibit cell proliferation and induce apoptosis by generating ROS. The results showed that the nanoformulation was 66.2% more effective than either drug alone in reducing tumor growth, suggesting that the synergistic effect of PPI and Mn^2+^ enhances the cGAS–STING-mediated immune response. Moreover, this novel strategy could enhance anti-tumor immune responses and reprogram inhibitory TME by promoting DC maturation, CD8^+^ infiltration, recruitment, and type I IFN production. Taken together, this dual activation strategy can effectively enhance the cGAS–STING pathway and convert “cold” tumors into “hot” tumors. In addition to the use of immune activators for co-stimulation, there have been studies of the synergistic use of PTT methods. For example, scholars have proposed a strategy based on PTT-mediated ICD effects and dual adjuvant synergistic enhancement of cGAS–STING pathway activation^181^. A metallo-micellar nano-vaccine was developed by self-assembly of a STING agonist (ABZI), Mn, and naphthocyanine (ONC)-coordinated nanoparticles (ONC–Mn-A) in maleimide-modified Pluronic F127 (MalF127) micelles (Fig. 7A). This study showed that ONc-Mn-A-malF127 increased IFN-*β* levels *in vivo* by 324-fold and 8-fold, respectively, compared to Mn or ABZI alone, owing to the synergistic effect between Mn and ABZI (Fig. 7B). At the same time, activation of the cGAS–STING pathway induces full maturation of DCs and induces death of CD8^+^ T cell-sensitive and CD8^+^ T cell-resistant tumor cells by simultaneously increasing CD8^+^ T cell and NK cell activity (Fig. 7C). In addition, ONc, as a Mn chelator and an efficient photosensitizer, was able to induce photo-induced ICD in tumor cells under laser irradiation, which led to the release of DAMPs and neoantigens, which were also captured *in situ* by malF127 in the tumor cells and then transported to the DC. The antigen capture ability of ONc-Mn-A-malF127 (pristine F127 nanoparticles) was investigated with significantly reduced hydrodynamic particle size and zeta potential. In contrast, the hydrodynamic particle size and zeta potential of ONc-Mn-A-F127 were significantly reduced without significant changes (Fig. 7D and E). Thus, ONC-Mn-A-MalF127 provides a nano platform that combines metal immunotherapy with light-induced immunotherapy to enhance the antitumor effects synergistically.

**Figure 8:**
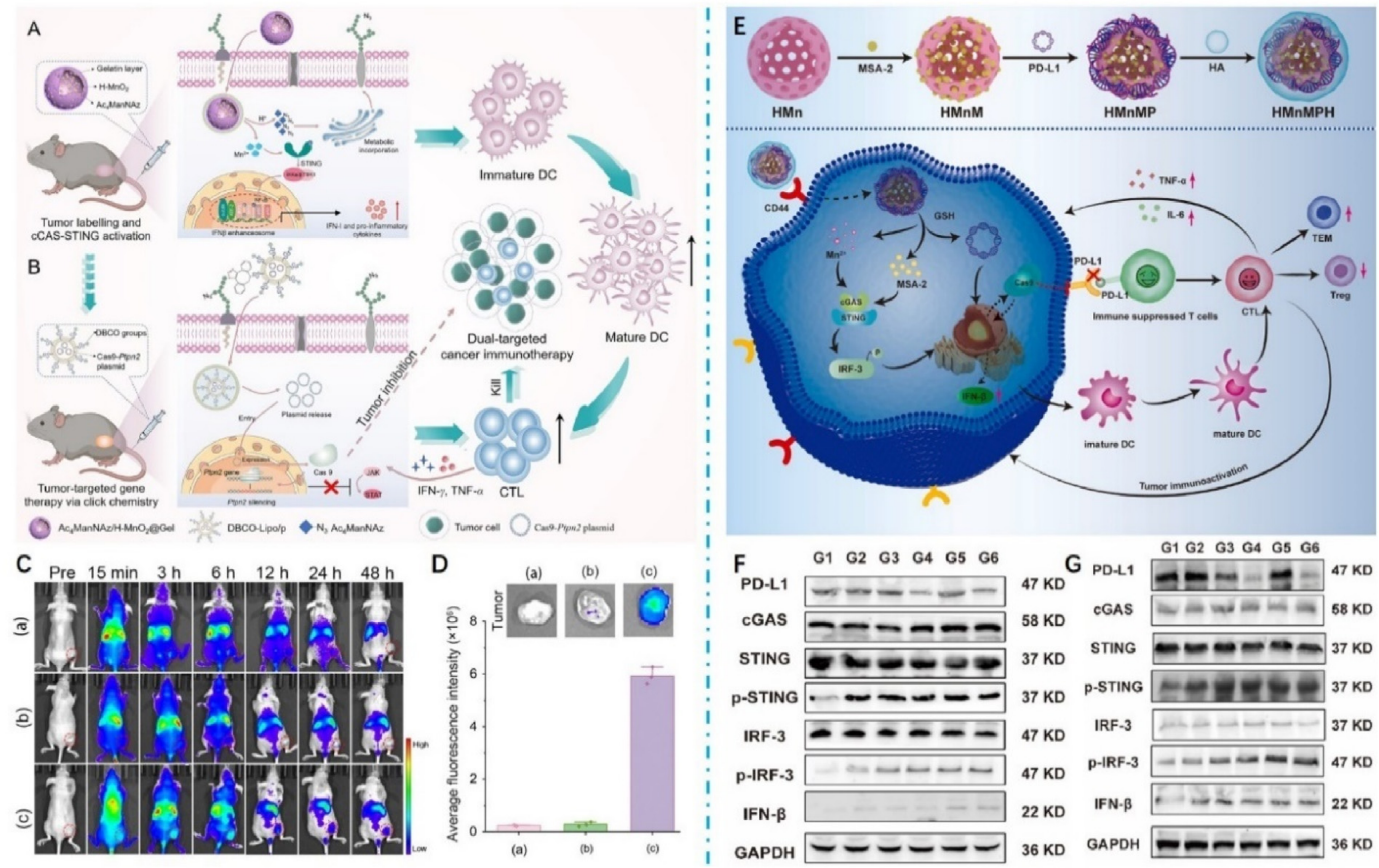
Mn-based nanoparticles and CRISPR/Cas9 synergize to regulate the immune status of the body. (A) Process of the selective labeling of tumor tissues *via* metabolic engineering and the activation of the cGAS–STING pathway by Mn^2+^ ions simultaneously by Ac_4_ManNAz/H–MnO_2_@Gel. (B) Illustration of targeted delivery of CRISPR/Cas9 system and tumor-specific gene editing of *Ptpn2* by DBCO-Lipo/p *via* bioorthogonal click chemistry. (C) *In vivo* fluorescence images of A375 tumor-bearing mice after intravenous injection of DBCO-Lipo/p at different time points, pretreated daily with (a) PBS, (b) H–MnO_2_@Gel, and (c) Ac_4_ManNAz/H–MnO_2_@Gel (5 mg Ac_4_ManNAz per kg) for two days. (D) *Ex vivo* fluorescence images of tumors harvested from mice at 48 h from (B) and tumors' corresponding average DiR fluorescence signal^184^. Reprinted with the permission from Ref. 184. Copyright 2023 Wiley-VCH GmbH. (E) Design and immunotherapeutic functions of HMnMPH. (F) Western blot analysis of PD-L1, cGAS, STING, p-STING, IRF-3, *p*-IRF3, and IFN-*β* expression levels in 4T1 cells pretreated with PBS (G1), HMnH (G2), MSA-2 (G3), HMnPH (G4), HMnMH (G5) and HMnMPH (G6). (G) Western blot analysis of PD-L1, cGAS, STING, p-STING, IRF-3, pIRF-3, and GAPDH expression of the sacrificed tumor on the 15th day. (h) PD-L1, TUNEL, and Ki67 staining of tumor sections after different treatments. Scale bar: 100 μm. Mean ± SD, *n* = 5. Student's *t*-test, ∗*P* < 0.05, ∗∗*P* < 0.01 *versus* control^190^. Reprinted with the permission from Ref. 190. Copyright, 2022 Elsevier.

In addition to these synergistic approaches, Mn-based nanomaterials can be combined with CRISPR/Cas9 technology to enhance anti-tumor immunity. It has been shown that CRISPR and CRISPR/Cas9 technology could significantly increase the number of immunotherapeutic approaches to cancer^182^^,^^183^. Yang et al.^184^ 2023 first proposed a bioorthogonal response-mediated CRISPR/Cas tumor-targeted delivery system that combines the STING activation pathway and the protein tyrosine phosphatase non-receptor type 2 Gene deletion mechanism, resulting in a significant enhancement of anti-tumor immunity. In this study, scholars constructed an innovative biodegradable hollow MnO_2_ nano platform to activate tumors’ cGAS–STING pathway and *in vivo* metabolic labeling (Fig. 8A and B). Among other things, CRISPR/Cas9 system-loaded liposomes can be modified to accumulate in tumor tissues using click chemistry, thereby increasing the sensitivity of tumors to immunotherapy. This bioorthogonal reaction-mediated delivery system enhances the tumor-targeting efficiency of the gene editing system and improves the effective editing efficiency *in vivo* (Fig. 8D). The study results showed that the fluorescence intensity of the isolated tumor tissue in this nano platform was much higher in the treated group than in the non-targeted control group, indicating enhanced accumulation of the nano platform in tumor tissue (Fig. 8C). In summary, this platform can enhance anti-tumor immune responses by stimulating innate and adaptive immunity. It has also been shown that CRISPR/Cas9 gene editing technology can overcome some of the drawbacks of ICB, but it is highly selective and irreversible^185^, ^186^, ^187^, ^188^. Moreover, ICB therapy works with Mn-based nanomaterials to co-stimulate the production of IFN, thereby improving immune function^189^. Lu et al.^190^ created a hollow Mn dioxide (HMn)-based nano platform loaded with a CRISPR/Cas9 plasmid to silence PD-L1 and a novel STING agonist, MSA-2, and coated it with hyaluronic acid (HA) (Fig. 8E). Compared to other Mn formulations, the nano platform not only activated the cGAS–STING pathway. It can also be combined with CRISPR/Cas9 gene editing for cancer immunotherapy. One of them, HMn, synergizes with MSA-2 to recruit and stimulate TBK1 to trigger downstream signaling events TBK1 and IRF3 (Fig. 8F). CRISPR/Cas9 knockout technology can irreversibly inhibit the PD-L1 immune checkpoint, awakening immunosuppressed T cells from differentiating into cytotoxic T lymphocytes, thus triggering a series of robust immune responses to suppress cancer cells (Fig. 8G). In summary, the nano platform achieved dual-channel activation of the cGAS–STING pathway with irreversible inhibition of PD-L1 immune checkpoint, thereby enhancing cancer immunotherapy.

Furthermore, Mn ions can trigger the NLRP3 inflammasome and induce pyroptosis *via* the caspase-1 (Casp1) pathway. Thus, Mn^2+^ is considered a DAMP that triggers innate immune cells to produce type I IFN, activate inflammasomes, or directly induce the prophylactic death of infected cells against pathogenic infections or cancer. In addition to activating the immune response *via* the traditional cGAS–STING pathway, Mn^2+^ can activate downstream signaling factors by initiating the TLR4-related signaling pathway. It has been shown that TLR4-induced activation of NF-*κ*B enhances stimulatory immune responses^191^^,^^192^. Professor Xia Bing's team at Nanjing Forestry University published a study that provided a new perspective for a deeper understanding of Mn ion immunoregulatory mechanisms^193^. This study found that trace amounts of Mn^2+^ can bind to bovine serum albumin (BSA) to form Mn@BSA and stimulate pro-inflammatory responses in human or murine macrophages *via* a TLR4-mediated signaling cascade. Mn@BSA relies primarily on the TLR4 signaling cascade to activate the immune response rather than the cytoplasmic Mn^2+^-activated cGAS–STING pathway or the NLRP3 inflammatory vesicle pathway. Mn@BSA can promote the secretion of cytokines such as IFN-*β*, IL-6, and TNF-*α* through the activation of downstream transcription factors such as NF-*κ*B and IRF7, thereby effectively reversing the immunosuppressive microenvironment of solid tumors. In addition, Mn@BSA can be assembled into nanowire structures based on GSH reduction and further amplify and prolong its immunostimulatory effect through macrophage phagocytosis pathway, secretion of cytokines, such as IFN-*β*, IL-6, and TNF-*α*, as well as promoting the expression of CD64, CD80, and CD86. These results highlight the importance of nanostructures of immune adjuvants in activating the body's intrinsic immunity and provide an entirely new perspective for understanding the mechanism of Mn ion immune activation. The TLR4-related signaling pathway, in concert with the cGAS–STING pathway, can co-activate immune responses. For example, Zhaohui Wang's team constructed a precise pH-responsive, TME-targeted nanoparticles (PMM NPs) encapsulating the TLR4 agonist Mpla and the STING agonist tetramanganese tetroxide NPs for the spatiotemporal orchestration of synergistic innate stimuli for anti-cancer immunotherapy^194^. Notably, this study used ultra-pH-sensitive (UPS) polymers with sharp pH responsiveness and precise TME targeting capabilities^195^^,^^196^. This property ensures that NPs are preserved intact in normal tissues but rapidly dissociate into monomers in acidic tumor tissues, rapidly releasing agonists to activate the TLR4 and STING pathways efficiently. TLR4-mediated NF-*κ*B enhances the extent of STING cascade activation and synergistically induces secretion of type I IFN and other inflammatory cytokines and activation of NK cells and specific T-cell immune responses, linking innate immunity to adaptive immune responses. Furthermore, PMM NPs could alleviate the immunosuppressive nature of the TME by significantly reducing the percentage of Tregs by polarizing M2 macrophages to M1 macrophages. Thus, this study confirms the potential of tumor-targeting nanoparticles to enhance STING immunostimulation, thereby expanding a new pathway for developing next-generation Mn-based nanomaterials.

In summary, Mn-based nanomaterials can activate immune responses through multiple pathways. First, Mn-based nanomaterials can increase the affinity of cGAS for double-stranded DNA, promote the activation of the STING cascade, and activate the cGAS–STING pathway in APCs, which enhances the tumor-specific T-cell response and increases the production of pro-inflammatory cytokines and chemokines. With this in mind, researchers have designed Mn-based nanoplatforms to activate the cGAS–STING pathway. Mn-based nanomaterials have also been used with other cGAS–STING pathway activators to achieve the dual activation of immune responses. Second, Mn-based nanomaterials can be applied synergistically with CRISPR/Cas9 technology and ICB therapies to trigger a more robust anti-tumor immune response. Finally, Mn-based nanomaterials can also trigger downstream signaling molecules by activating the TLR4-related signaling pathway and NLRP3 inflammatory vesicles, which in turn activate the immune response.

#### 5.1.4 Using magnetic resonance imaging (MRI) to monitor tumor immunotherapy in real-time

In recent years, the range of treatments for cancer has greatly improved, but because malignant tumors develop too quickly, early diagnosis is needed. Early diagnosis can greatly improve the treatment effect and survival rate of patients. Imaging plays a significant role in diagnosing early-stage tumors and staging tumor development^197^. Currently, the main imaging modalities are Computed Tomography (CT), Magnetic resonance imaging (MRI), and Positron Emission Computed Tomography (PET). Among these, MRI is currently a research hotspot for imaging and screening. It is based on the principle of nuclear magnetic resonance (NMR). It provides high-resolution images by monitoring the difference in the relaxation rate of water molecules (r*i* = 1/Ti) in various tissues in the body^198^^,^^199^. Where *i* = 1 or 2 represents the longitudinal relaxation rate (r1) and transverse relaxation rate (r2), respectively, and generally the higher the relaxation rate, the better the image contrast at the same concentration. Compared to CT imaging, MRI has several imaging parameters and a high degree of soft-tissue resolution, and it can enable non-invasive monitoring of tumorigenesis and progression. The key to MRI is the issue of sensitivity *versus* prolonged detection time, and these concerns can be addressed through external MRI contrast agents. In MRI, when a contrast agent comes into contact with a water molecule, it can change the magnetic field environment of the water molecule and shorten its transverse and longitudinal relaxation times of the water molecule. These changes can enhance the contrast between the target tissue and surrounding tissue, resulting in a brighter picture. In addition, due to the unrestricted magnetic field, MHT is more suitable for deep tumor s than PTT.

**Figure 9:**
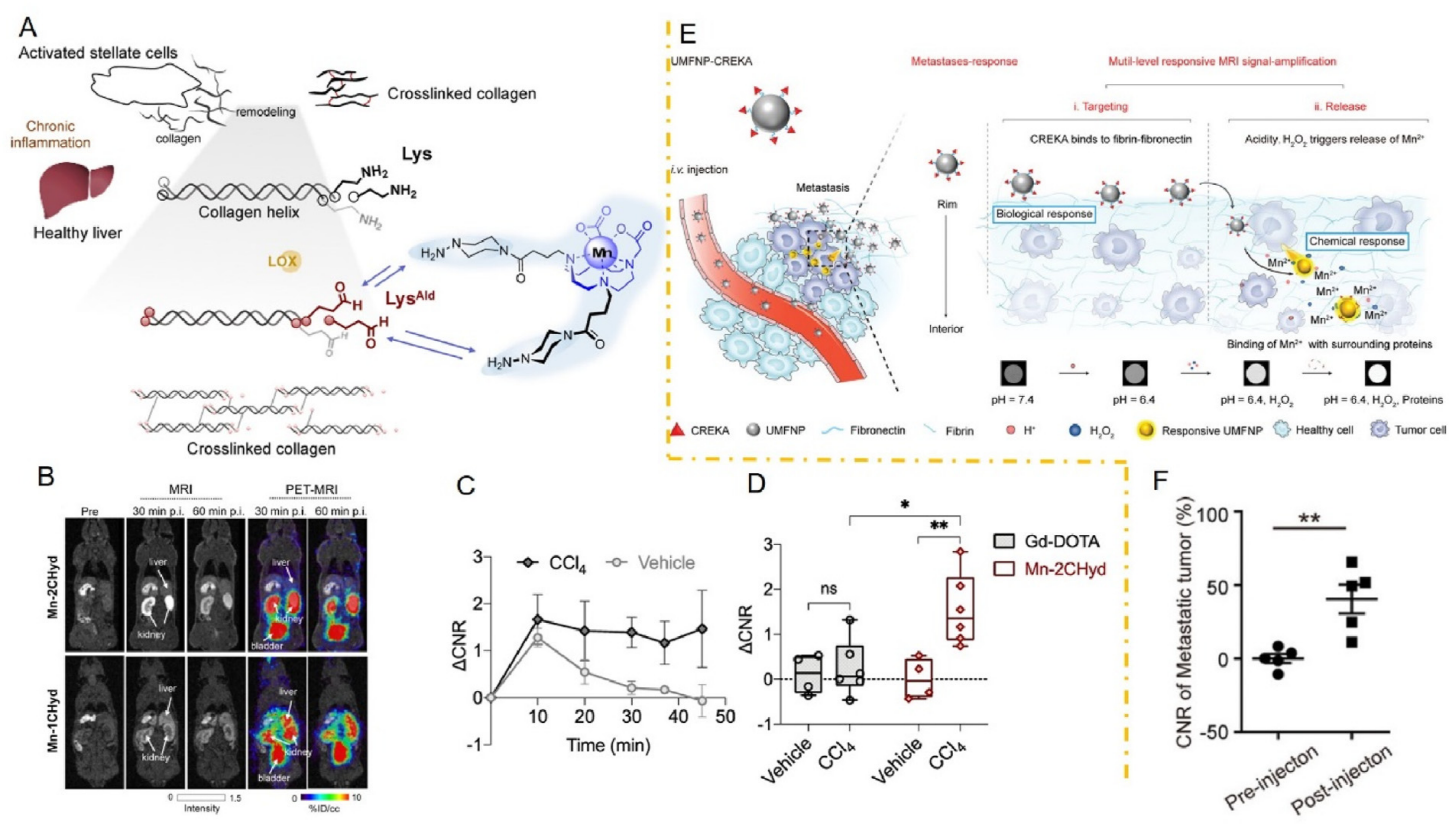
Mn-based nanoparticles for detecting liver disease progression. (A) Schematic illustration of the design of reversible and dual binding Mn^2+^probes for targeting Lys^Ald^ residues in the process of liver fibrogenesis. (B) Whole body MRI (grey scale) and PET-MRI (color scale) images of normal mice imaged at pre-, 30- and 60 min post-injection of 0.1 mmol/kg [^52^Mn] Mn-2CHydor [^52^Mn] Mn-1CHyd. PET imaging is reported as percent injected dose per cubic centimeter (% ID/cc); (C) Post-pre change in the liver to muscle contrast to noise ratio (ΔCNR) change over time for CCl_4_ and vehicle-treated mice imaged with Mn-2CHyd: (D) ΔCNR in vehicle-treated (*n* = 4) and CCl_4_mice (*n* = 6) at 45 min p.i. Of Gd-DOTA and Mn-2CHyd. ∗*P* < 0.05, ∗∗*P* < 0.01, ns, not significant, unpaired student's *t*-test^203^. Reprinted with the permission from Ref. 203. Copyright 2022, American Chemical Society. (E) A bioinspired nanoprobe with multilevel responsivet1-weighted MR signal-amplification illuminates ultrasmall metastases. (F) CNR analysis of metastatic tumors before and after contrast agent injection. ∗∗*P* < 0.01^205^. Reprinted with the permission from Ref. 205. Copyright 2019, WILEY.

Because Mn^2+^ has a high spin number and long electron relaxation time, and thus a stronger relaxation rate, this helps to enhance the contrast of MRI images^200^. In addition, compared with the Gd series of paramagnetic metal complexes, which are commonly used as T1 contrast agents in clinical practice, Mn complexes are biocompatible and can be eliminated from the body through renal excretion after use. During liver fibrosis, chronic liver injury leads to the activation and proliferation of stellate cells, which in turn remodels the extracellular matrix (ECM). Upregulation of the lysyl oxidase (LOX) family of enzymes catalyzes the oxidation of lysine *ε*-amino acids on the ECM (mainly collagen) to form aldehyde lysine (Lys^Ald^), which then undergoes cross-linking reactions with other proteins to stabilize fibrotic ECM. Professor Peter Caravan's group^201^^,^^202^ at Harvard Medical School demonstrated in a previous study that Lys^Ald^ is a biomarker of fibrogenesis and constructed gadolinium (Gd) magnetic resonance probes that can covalently bind to Lys^Ald^ *in vivo* to quantify the development of fibrosis. However, concerns about the long-term safety of Gd contrast agents have led the group to design and synthesize novel hydrazine functional group-modified Mn-based MRI probes for the detection of hepatic fibrosis^203^. The authors constructed a double-bound Mn-based probe based on cis-Mn-1,4-DO2A, which has antioxidant and high thermodynamic stability (Fig. 9A). This “double-locked” approach can effectively increase the reaction rate of Lys^Ald^ and provide a high relaxation state upon binding. When detecting hepatic fibrosis *in vivo*, Mn-2CHyd showed stronger hepatic MR signals and slower hepatic clearance rates in liver fiber model mice compared to negative control contrast agents (Fig. 9B–D). Therefore, Mn-2CHyd has a high sensitivity and specificity when detecting liver fibrosis *in vivo*.

In addition to the use of Lys^Ald^ as a marker for the development of liver disease, there have been studies using ROS levels as a marker for the degree of liver disease development. Nonalcoholic steatohepatitis (NASH) is a major classification of liver disease, and its pathogenesis at the molecular level is unclear. Several studies have shown that ROS from fatty acid oxidation and oxidative excitation from reactive nitrogen in hepatocyte mitochondria are key factors in the development of NASH. Sangyong Jon and Sung-Hong Park of the Korea Advanced Institute of Science and Technology (KAIST) designed probes for MRI imaging by detecting ROS levels^204^. The team's early discovery that endogenous antioxidant bilirubin-derived nanoparticles (BRNPs) could act as endogenous metal chelators led to the use of Mn^2+^-chelated bilirubin nanoparticles (Mn@BRNPs) as ROS-responsive MRI imaging probes. Mn@BRNPs released free Mn^2+^ after intravenous injection, and they enabled longitudinal T1-weighted MR imaging of NASH model mice with signal intensity dependent on hepatic ROS concentration. Meanwhile, after Mn@BRNP injection, the disease progression within eight weeks and the critical transition to a cirrhosis-like stage could be detected based on the AUC value calculated from the L/M T1 magnetic resonance signal ratio, which was in good agreement with the changes in ROS in the liver measured by DHE staining. In summary, this study serves as a non-invasive and longitudinal imaging tool for monitoring the progression of NASH and for evaluating therapeutic interventions.

In addition to its use in detecting disease progression, Mn^2+^ can also be used to detect tumor metastasis. For example, a study designed an ultrasensitive T1-weighted MRI contrast agent (UMFNP-CREKA) based on ultra-small Mn ferrite nanoparticles (UMFNPs) (Fig. 9E)^205^. It has been shown that UMFNPs have multiple functions, on the one hand, inducing a significant amplification of the good T1-weighted magnetic resonance signal due to local Mn^2+^ release and significantly improving r1 relaxation, and on the other hand, minimizing the elimination of the reticuloendothelial system (RES) and improving the *in vivo* blood circulation time. In addition, to improve the efficiency of identifying the biological parameters of metastatic tumor tissues, the study also modified the UMFNPs with the pentapeptide CREKA (Cys-Arg-Glu-Lys-Ala). CREKA has a high affinity for fibronectin and its complexes and can direct UMFNPs to the metastatic margins, whereas fibronectin is a marker of epithelial-to-mesenchymal transition in the ECM of high-risk breast cancers (Fig. 9F). Thus, UMFNP-CREKA has a multi-response signal amplification function, which may contribute to the early detection of ultrasmall metastases in the clinic. In summary, Mn^2+^ can bind different substances to detect diverse markers of disease occurrence, thus constituting a precise and sensitive imaging modality.

In summary, Mn-based nanomaterials have various applications in immunotherapy. Mn-based nanomaterials can be used as carriers to deliver drugs, and they can also alleviate the immunosuppressive microenvironment by regulating oxygen status, promoting the reprogramming of TAMs, and modulating immune checkpoints. One of the most important factors that sets Mn apart from other metals is their ability to activate the immune response. Mn-based nanomaterials can trigger immune responses by activating the cGAS–STING and TLR4-related signaling pathways. Mn-based nanomaterials also have MRI imaging capabilities owing to the properties of Mn. Therefore, Mn-based nanomaterials play a crucial role in anti-tumor immunity, creating new ideas and therapeutic options for cancer immunotherapy and showing great promise for clinical applications.

### 5.2 Pt-based nanoparticles for immunotherapeutic applications

Since 1978, when cisplatin compounds were introduced clinically as anticancer drugs, Pt compounds have been one of the most widely used anticancer drugs for half a century^206^. All Pt drugs currently approved for marketing are square-plane bivalent Pt, including cisplatin, carboplatin, and oxaliplatin. Their anticancer mechanism mainly targets DNA and forms Pt-DNA adducts by liganding with DNA to block DNA synthesis, thus inducing cancer cell death^207^^,^^208^. Pt-based drugs are still used today and have many drawbacks. For example, non-specific DNA damage and DNA damage repair mechanisms, the ability of complexes produced after entry into the body to react with serum albumin and other proteins in the bloodstream to cause inactivation^209^, and the ability of intracellular metallothionein and glutathione to strongly bind and segregate cisplatin reduce therapeutic efficacy^210^. Consequently, Pt-based drugs are clinically limited by systemic toxicity and drug resistance^211^^,^^212^. Therefore, exploring and studying the novel therapeutic mechanisms of Pt drugs is necessary. When stressors, such as chemotherapeutic drugs or radiotherapy, stimulate tumor cells, they also upregulate the expression of specific characteristic molecules during apoptosis. These molecules are known as DAMPs^213^^,^^214^. Expression of DAMPs effectively stimulates the activation and recruitment of a wide range of immune cells. It can improve antigen presentation and tumor recognition by APCs, subsequently promoting DC maturation and differentiation. DC mature to activate CTL-driven adaptive immune responses and increase IFN-*γ*, IL-2, and IL-4 release, thereby establishing robust antitumor immune responses^215^^,^^216^. This reaction is known as ICD. Pt drugs, as type I ICD inducers, can cause endoplasmic reticulum stress after entering tumor cells, significantly upregulating the expression of DAMPs, which activates DC cells and enhances their antigen-presenting ability, and ultimately strengthens the body's anti-tumor immunity^217^.

Besides causing ICD, Pt drugs significantly reduce the inhibitory molecule PD-L2 on DCs and tumor cells and enhance the secretion of pro-inflammatory cytokines (IFN-*γ* and IL-2) and antigen-specific proliferation^218^. At the same time, it has been shown that chronic inflammation can inhibit immune surveillance to promote inflammation development through multiple pathways, *e.g.*, cyclooxygenase-2 (COX-2) can upregulate PD-L1 expression. Thus, many Pt compounds with anti-inflammatory effects are expected to modulate immune checkpoints and alleviate the immunosuppressive TME. Remarkably, the combined approach of immune checkpoint therapy with Pt has yielded outstanding results in lung cancer. For example, a recent phase III trial reported excellent results in treating patients with TNBC treated with a combination of pembrolizumab and Pt drugs. Pt complexes can also act as immunostimulants, activating DC, NK cells, and effector T cells, thereby alleviating the immunosuppressive TME. Pt drugs can be combined with novel therapies such as PDT and PTT. PDT and PTT have become an emerging field of tumor therapy because of their less invasive nature, low toxicity, and repeatability. In addition, PTT not only directly ablates tumors but also induces the onset of ICD response, releasing DAMPs and TAAs, thereby promoting the maturation of APCs and the activation of T-cells, amplifying anti-tumor immune responses, and enhancing the immune response rate. Therefore, Pt-based nanoparticles are used synergistically with PTT to enhance ICD response, reduce drug toxicity, and induce a long-term anti-tumor immune response in the body to maximize the therapeutic effect. It is worth noting that, unlike other metallic elements, platinum elements do not act as carriers in metallic nanoparticles, but rather act as drugs.

#### 5.2.1 Platinum-based nanoparticles as inducers of ICD for synergistic immunotherapy

**Figure 10:**
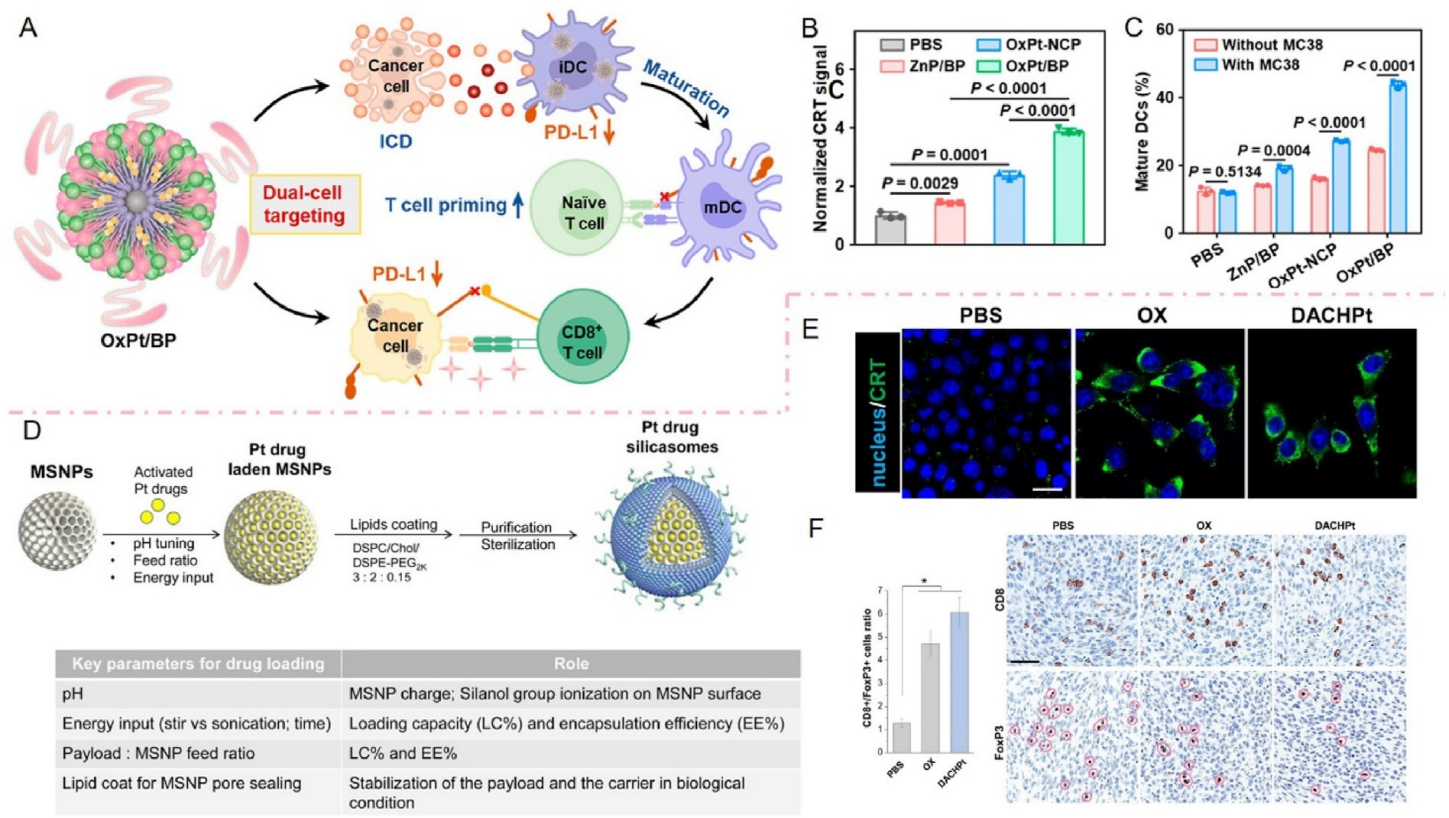
Pt-based nanoparticles as inducers of immunogenic cell death for immunotherapy. (A) Immunogenic bifunctional nanoparticle suppresses programmed cell death-ligand 1 in cancer and dendritic cells to enhance adaptive immunity and chemo-immunotherapy. (B, C) CLSM images (scale bars: 20 μm) (B) and flow cytometry quantification. (C) Of CRT exposure (green) on MC38 cells (*n* = 3)^219^. Copyright 2024, American Chemical Society. (D) The top panel provides a schematic that outlines the synthesis steps for the tailored construction of silicasomes, incorporating the active Pt drug. The key parameters that govern successful drug loading are outlined in the table (lower panel). (E) The tumor tissue was used for IHC analysis of CD8 and Foxp3 T cell appearance, allowing us to calculate a CD8/Foxp3 ratio. Data are expressed as mean ± SEM, *n* = 6. ∗, *P* < 0.05 (one-way ANOVA followed by a Tukey's test). The bar is 50 μm. (F) Confocal microscopy showing the appearance of CRT on the KPC cell surface treated with oxaliplatin or DACHPt (500 μmol/L) for 24 h. The bar is 20 μm. Green: CRT; Blue: Nuclear^221^. Reprinted with the permission from Ref. 221. Copyright 2021, Wiley.

Platinum-based drugs can also induce ICD synergistic immunotherapy. Unlike the usual apoptotic approach, the ICD process is a collaborative immune activation program involving multiple processes from innate to acquired immunity. One study constructed nanomedicines based on the ICD-inducing properties of Pt-based drugs to enhance antitumor immunity. For example, Professors Wenbin Lin and Ralph R. Weichselbaum of the University of Chicago constructed a nanoscale coordination polymer (NCP) with chemo-immunotherapeutic functionality, OxPt/BP, which was loaded with oxaliplatin (OxPt) and 2-bromohexadecanoic acid (BP), allowing dual-cell targeting (Fig. 10A)^219^. OxPt/BP can promote DC maturation by increasing intracellular oxidative stress and enhancing the OxPt-induced immunostimulatory ICD (Fig. 10B and C). Meanwhile, BP can effectively prolong the blood circulation time and increase the accumulation of drugs in the tumor, target cancer cells, and DCs, thus effectively reducing PD-L1 expression. In addition, studies have shown that systemically administered OxPt/BP promotes CTL infiltration and activation and reduces the number of immunosuppressive Tregs. Which in turn inhibits the growth of subcutaneous and *in situ* colorectal cancers. In summary, the agents constructed in this study have significant potential to induce ICD and reprogram DC for effective cancer therapy simultaneously.

Although Pt compounds are the most successful metalloid anticancer drugs, they still have toxic side effects. One study coupled oxidized oxaliplatin (OXA) with 2-bromo-1-(3,3-dinitro-1-azetidinyl) ethanone (RRx-001) to synthesize an epigenetic Pt complex (OXA-NO)^220^. OXA-NO crossed the cell membrane more easily than OXA, and the cellular uptake rate was higher. OXA–NO–treated mouse colon cancer cells showed high expression of “eat me” CRT signals, while “don't eat me” CD47 signals were significantly downregulated, breaking the balance between signals. This signal ratio indicates phagocytosis of cancer cells by macrophages. The CRT/CD47 ratio after incubation with OXA-NO was much higher than after incubation with OXA, suggesting that cancer cells incubated with OXA-NO were more susceptible to phagocytosis by macrophages. In addition, flow cytometry analysis showed that OXA-NO could promote a significant decrease in tumor M2-type CD206^+^ and a significant increase in anti-tumor M1-type CD11c^+^ by releasing NO in hypoxic tumors, with a shift in TAM from M2-type to M1-type. Therefore, compared to OXA, OXA-NO has higher safety and efficacy and can induce ICD more efficiently, thus activating the immune system.

In addition to chemically modifying platinum compounds to improve efficacy, nanocarriers can be constructed to enable efficient loading of platinum drugs and improve efficacy. Huan Meng and Andre E. Ne of the University of California have developed a platform based on mesoporous silica nanoparticles (MSNPs) that can be used for high-dose loading of a range of activated Pt compounds (Fig. 10D)^221^. Instead of using post-grafting methods, this study exploited the pH-dependence of the surface silanol groups for electrostatic and ligand binding of oxaliplatin and a uniform lipid coating to seal the particle pores. This strategy also applies to other Pt compounds for generating carriers with high stability and loading capacity. In addition to improved drug delivery, encapsulated oxaliplatin had better pharmacokinetics and intra-tumor delivery efficiency than free drugs in an *in situ*-derived pancreatic cancer model. Remarkably, it was shown that the injection of silica vesicles loaded with oxaliplatin-induced higher expression of CRT and release of HMGB1, which could cause a more efficient ICD response (Fig. 10E and F). Therefore, the nanocarrier enables efficient loading and delivery of Pt-based drugs and induces a more efficient ICD reaction. This provides a new approach to developing nanocarriers for Pt-based drugs.

#### 5.2.2 Platinum-based nanoparticles used to stimulate and activate immune responses

Studies have shown that chemotherapeutic agents such as Pt also activate the cGAS–STING pathway, which can be enhanced by damaging DNA in tumor cells and releasing it into the cytoplasm, where it binds to cGAS, activates the cGAS–STING pathway, and improves the effect of immunotherapy.

**Figure 11:**
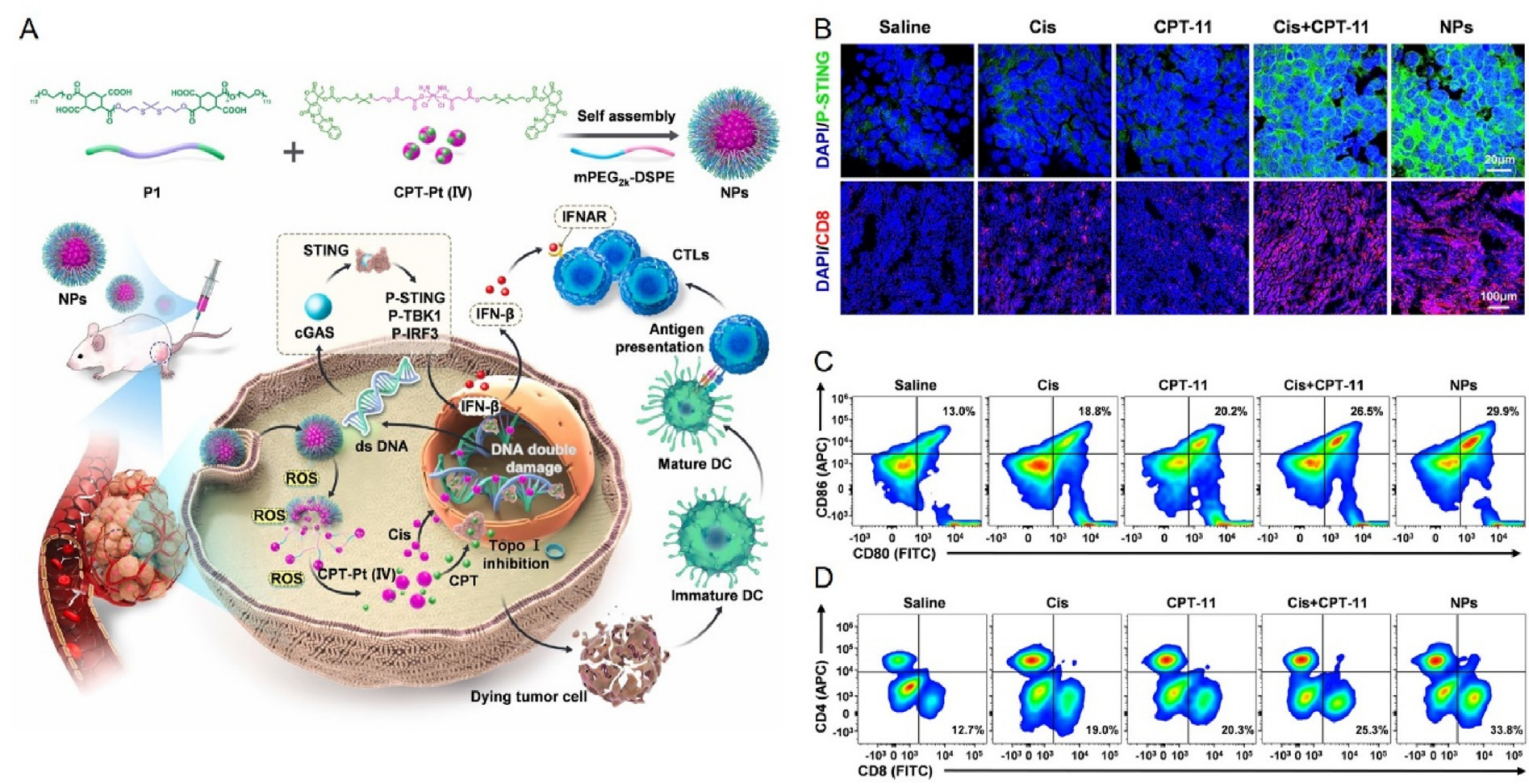
Hybrid Pt prodrugs for immunotherapy through activation of the cGAS–STING pathway. (A) The schematic illustration of NPs mediated activation of cGAS–STING pathway *via* DNA double damage. (B) Immunofluorescence imaging of p-STING expression and the infiltration of CD8 T cells in CT26 tumor upon different treatments. (C) Flow cytometry study of CD80 CD86 DCs gating on CD11c cells in lymph nodes. (D) Representative flow cytometry study of CD8 and CD4 T cells gating on CD3 cells in tumors^222^. Reprinted with the permission from Ref. 222. Copyright 2022, Elsevier Ltd.

Carboplatin was found to induce DNA damage and activate the classical STING/TBK1/IRF3 pathway and the non-classical STING–NF–*κ*B signaling complex *via* the STING signaling core. This process increases CD8^+^ T-cell infiltration and PD-L1 expression, transforming a “cold tumor” into a “hot tumor”. In contrast to other metallic elements that activate the cGAS–STING pathway, platinum compounds exhibited immunomodulatory effects on the TME, promoting the release of tumor neoantigens and facilitating the expanded secretion of antigen-presenting cells. Therefore, some studies have designed cisplatin and Camptothecin (CPT) as a mixed platinum prodrug (CPT-PT (IV)) and self-assembled CPT-PT (IV) with high hydrophobicity and ROS-sensitive polymer (P1)mPEG2k-DSPE into ROS-responsive nanoparticles (NPs) (Fig. 11A)^222^. In a mouse model of colorectal cancer, nanoparticles could be targeted to aggregate at the tumor site and release cisplatin with CPT under ROS, leading to dual DNA damage. The study showed that IFN-*β* levels in the peripheral blood of nanoparticle-treated mice (106.35 pg/mL) were 1.38-fold and 1.57-fold higher than cisplatin alone (77.15 pg/mL) CPT (67.91 pg/mL) (Fig. 11C and D). This demonstrated that NPs could increase the infiltration level of CD8^+^ T cells, release IFN through the cGAS–STING pathway, and promote DC cell maturation (Fig. 11B). This study used nano-systems to deliver common DNA-targeted drugs to activate the cGAS–STING pathway and enhance chemotherapy and immunotherapy synergy.

In addition to targeting ncDNA, it has also been found that mitochondrial DNA (mtDNA) released from mitochondrial membrane remodeling can activate the intracellular STING pathway, promoting the accumulation of inflammatory factors and inducing liver injury. Recent studies have shown that acetaminophen (APAP) releases mtDNA and causes mitochondrial membrane remodeling by inhibiting the mitochondrial protein Sam50, which connects inner and outer mitochondrial membranes. Therefore, it has been investigated that a novel platinum compound, OAP2, was constructed and synthesized by introducing APAP in the oxaliplatin axial ligand^223^. OAP2 targets mitochondria by down-regulating Sam50 protein expression to promote mitochondrial membrane remodeling, which in turn promotes the recruitment of Bcl-2-associated X protein proteins and the formation of a pore in the mitochondrial membrane to release mtDNA. In addition, OAP2 binds to ncDNA produced by DNA damage and synergistically stimulates the cGAS–STING pathway. These effects improve the immune activation of conventional Pt-based drugs. In addition, a large amount of ROS generated by the entry of the prodrug OAP2 into cells can promote the maturation of the NLRP3 inflammasome, which can induce the onset of GSDMD-mediated cellular pyroptosis by activating caspase-1. *In vivo* activity studies demonstrated that the prodrug OAP2 significantly increased antitumor activity and reversed the tumor immunosuppressive microenvironment, improving the immune response. This dual strategy provides a new way of thinking about the activation of the STING pathway by platinum-based drugs and offers new insights into the design of anti-tumor drugs targeting mitochondrial function.

The chemotherapeutic agents cisplatin^224^ and CPT^225^ above activate the cGAS–STING pathway by triggering DNA fragments into the cytoplasm. There are also non-nucleotide STING agonists (*e.g.*, MSA-2^226^, MK-1454^227^, and ADU-S100^228^) and nucleotide-based STING agonists (*e.g.*, interferon-stimulated DNA (ISD)^58^, 2′-3′-cyclic GMP–AMP^229^, cyclic-di-GMP^230^, exo-STING). Thus, we can use both agonists synergistically to enhance the activation of the cGAS–STING pathway to prevent tumor metastasis and recurrence. For the first time, a team of researchers, Haihua Xiao, at the Institute of Chemistry, Chinese Academy of Sciences (CAS), designed a unique DNA tetrahedral nanostructure (TDNISDs) that consists of complementary pairing of ISD strands and three sequence-specific single-stranded DNA bases. TDNISDs can activate the STING pathway more effectively and have excellent immune activation effects^231^. To enhance the therapeutic efficacy, Pt (II) complexes 56MESS were incorporated into the supramolecular structure to generate synergistic nanoformulations for chemotherapy and immunotherapy (84bp-TDNISD/56MESS). 84bp-TDNISD/56MESS not only effectively activates the cGAS–STING pathway as a delivery vehicle, but also releases 56MESS for effective chemotherapy and synergistically activates the cGAS–STING pathway, thereby preventing tumor metastasis and recurrence. In summary, this study combined two types of STING agonists to achieve synergistic effects of chemotherapy and immunotherapy. It strongly activated the cGAS–STING pathway, providing a promising pharmacological approach to prevent tumor recurrence and metastasis.

In summary, researchers designed immune-activated nanoplatforms to enhance anti-tumor immune responses based on the role of Pt compounds in activating the cGAS–STING pathway. Moreover, Pt-based drugs have also been used synergistically with other activators to achieve dual activation of the cGAS–STING pathway. Compared to Mn analogs that activate the cGAS–STING pathway, Pt analogs have the advantage of being used for dual activation by introducing other activators *via* axial ligands.

#### 5.2.3 Platinum-based nanoparticles for immunotherapy in conjunction with other approaches

The three primary traditional cancer treatments are surgical resection, radiotherapy, and chemotherapy. However, conventional treatments are harmful and prone to recurrence and metastasis. In contrast, immunotherapy can eliminate local and distant metastatic tumors and trigger long-term immune memory to prevent tumor recurrence and metastasis. However, inadequate immune response rates remain a significant obstacle in immunotherapy. Electromagnetic energy sources, such as magnetic fields, ultrasound (US), light, and X-rays, show great promise in cancer therapy, especially when combined with nanocarriers for drug delivery^232^. On the one hand, electromagnetic energy can trigger the release of drugs from carriers with a high degree of precision. It can also mediate magneto-thermal therapy (MHT), sonodynamic therapy (SDT), PTT, and radiotherapy (RT)^233^. On the other hand, electromagnetic energy not only directly ablates tumors but also induces the onset of the ICD response, releasing DAMPs and TAAs, which promotes the maturation of APCs and the activation of T-cells, amplifies the anti-tumor immune response and enhances the immune response rate^234^. Therefore, light therapy can enhance the immune response rate and enable tumor targeting. Light therapy consists mainly of PDT and PTT. PTT is a light irradiation photothermal agent at a specific wavelength, which causes the photothermal agent to heat up and thus kill the tumor cells. It can also improve the efficiency of local photothermal heating and tumor tissue ablation. PDT is a light irradiation at a specific light irradiation, which allows the photosensitizer to produce a large amount of ROS and thus kills the tumor cells^235^. It has been demonstrated that PTT can elicit a powerful immune stimulus through the ICD response, releasing tumor antigens and endogenous adjuvants^236^, ^237^, ^238^.

Therefore, Tang et al.^239^ set a precedent for reducing Pt (IV) precursor drugs based on NIR-II photoactivation to enhance the controllable release of Pt (IV) precursor drugs and enhance the penetration depth of the drugs in tumor tissues. They designed a pseudo-conjugated polymer, PSP-Pt, based on the Stille polymerization method with a polymer backbone containing the prodrug Pt (IV) of oxaliplatin. Moreover, PSP-Pt was assembled using the lipid polymer mPEG2k-DSPE to form NPPSP-Pt. NPPSP-Pt can be activated under appropriate NIR irradiation to accelerate the release of oxaliplatin from Pt (IV) reduction, which can then be used in tumor tissues for photothermal therapy and chemo-immunotherapy. The polymer has a triple function: the oxaliplatin prodrug Pt (IV) on the main chain of the polymer can be reduced to oxaliplatin in the presence of a reducing agent in the cancer cells, thus killing the cancer cells *via* chemotherapy. Moreover, oxaliplatin can be used as an ICD inducer to induce an ICD immune response. Second, the polymer can be efficiently activated by NIR-II light, and the heat generated by PSP-Pt accelerates the rate of reduction of the oxaliplatin prodrug Pt (IV), leading to improved chemo- and immuno-therapeutic efficiencies as well as selective drug delivery and release. In conclusion, nanoparticles can significantly improve the efficiency of synergistic chemotherapy and immunotherapy treatment based on NIR-II light.

Although PTT is minimally invasive, long-lasting, and safe, an intense laser (local temperature up to 50 °C) may cause burns to normal cells near the tumor while destroying the cancer^240^. Therefore, achieving lower temperatures (less than 42 °C) for PTT is of great value for future clinical translation of optical cancer therapy. Wang et al.^241^ present a comprehensive strategy that not only takes a mild hyperthermia approach but also systematically targets the critical factors involved in the cisplatin resistance process. The researchers formed F-NPs with photothermal effects by complexing the novel photothermal polymer DAP-F with reduction-sensitive amphiphilic polymer P1. The 808 nm NIR laser can induce F-NPs to generate a mildly high temperature (43 °C), thereby increasing the permeability of the cell membrane and accelerating the uptake and accumulation of drugs in tumor cells. *In vitro* studies have shown that the anticancer effect of F-NPs in combination with Pt-nps on drug-resistant A549DDP cells under mild hyperthermia is significantly higher than that of Pt-nps without mild hyperthermia by nearly 2-fold and by more than 5-fold compared to that of cisplatin. This indicates that mildly high temperatures can inhibit cisplatin resistance in tumor cells. Therefore, the mildly high temperature generated by photothermal F-NPs under appropriate light can reverse the resistance of tumor cells to cisplatin. Meanwhile, the *in vivo* study showed a significant reduction in tumor volume after treatment with F–Pt-NPs (43 °C) compared to the other treatment groups, including the F–Pt-NPs group. The mean tumor mass of F-Pt-NP (43 °C)-treated mice (14 mg) was 1/23 that of F-Pt-NP-treated (322 mg) mice and 1/306 that of cisplatin-treated (4290 mg) mice. This suggests that the anticancer activity of the F–Pt-NPs can be significantly enhanced under mild hyperthermia.

Pt-based drugs have significant limitations owing to their uncontrolled release and low accumulation within tumors in advanced bladder cancer (BC)^242^, ^243^, ^244^. PTT controls the controlled release of drugs in addition to enhancing immune stimulation and overcoming resistance through mild hyperthermia. PTT allows nanocarriers to release drugs on demand and improve drug accumulation within the tumor. Therefore, One study designed a self-polymerizing Pt (II) PDA nanocomplex (PtPD) for the treatment of BC under near-infrared light using polydopamine (PDA)^245^. PtPD with high Pt loading (11.3%) can be decomposed under NIR light irradiation in combination with a reductive TME, and the released Pt ions can lead to cascade death of tumor cells and controlled release of chemotherapeutic agents to achieve highly effective chemotherapy. On the other hand, PDA possesses good biocompatibility^246^, ^247^, ^248^, biodegradability, and excellent photothermal conversion efficiency^249^, and can promote a more substantial photothermal effect to assist Pt-based immunotherapy, which can help improve the accumulation of drugs in the tumor. As a result of the study, the combination of PtPD and PTT showed excellent anti-tumor effects, with a tumor growth inhibition rate of up to 82.5%. In summary, combination therapy improves tumor outcomes, and PTT improves the characteristics of Pt compound release to some extent.

In recent years, it has also been shown that PDT, a process in which a photosensitizer reacts with oxygen under light irradiation to produce single-linear oxygen (^1^O_2_) and ROS, not only directly destroys tumor cells but also triggers immunogenicity by effectively inducing ICD^235^. However, the limited penetration depth of light somewhat inhibits the antitumor effect. Therefore, the synergistic use of Pt compounds with PDT can adequately trigger the antitumor immune response, accurately release the drug locally, and reduce the toxicity of chemotherapeutic agents. However, the effect of PDT on tumors depends on the photosensitizers, O_2_, and lasers with specific wavelengths and intensities. Therefore, hypoxia in the TME can limit PDT efficacy^250^. For PDT to show better efficacy, researchers combined Pt nanoparticles with nanoparticle-based catalase-targeted delivery (CAT) to alleviate tumor hypoxia and improve PDT. To achieve nano Pt that is not limited by *in situ* growth for penetration into distant tumors, the first attempt to construct a liposomal drug delivery system using reverse-phase evaporation^251^ and M-*ϕ*-cell membrane^252^^,^^253^ camouflage was made in this study. The liposomes prepared by this novel method keep the Pt nanoparticles free and have long circulation and inflammatory endothelial targeting functions. After intravenous injection, owing to the CAT properties of liposomes, this nano Pt breaks down the high concentration of H_2_O_2_ in the tumor to provide oxygen, thus enhancing the efficacy of PDT. In addition, ^1^O_2_ released by PDT can disrupt the liposome membrane^254^^,^^255^ and Pt nano, hence achieving the controlled release of Pt drugs and reducing drug toxicity while improving tumor penetration. In summary, PDT achieved a controlled release of Pt drugs and enhanced ICD response, whereas nano Pt enhanced PDT efficacy through CAT properties. The two approaches act synergistically to enhance the efficacy of anti-tumor chemotherapy and immunotherapy.

Platinum-based drugs in combination with ICB therapy are one of the most effective forms of treatment^256^. Clinically used Pt-based drugs can promote T-cell infiltration by blocking the interaction between immune checkpoint receptors and their corresponding ligands on tumor cells. A randomized phase II trial in NSCLC showed that the combination of ipilimumab with carboplatin/paclitaxel improved PFS compared to chemotherapy alone^257^, and a phase III trial is underway. Platinum-based drugs induce ICD, increase the sensitivity of tumor cells to CTL cleavage, and promote the down-regulation of PD-L1. For example, cisplatin, oxaliplatin, and carboplatin significantly reduce the inhibitory molecule PD-L2 on DCs and tumor cells. Downregulation of PD-L2 induces antigen-specific proliferation and enhances the secretion of Th1 cytokines, further enhancing the immune response. Thus, platinum-based drugs may act synergistically with immune checkpoint therapies to enhance the immunogenicity and antigenicity of tumor cells by inducing ICD genesis to improve recognition and attack of tumor cells by immune cells and increase the rate of immune response. Therefore, one study coupled Pt nanoparticles with a small-molecule inhibitor of PD-L1 (BMS-1) to construct a heat-sensitive liposome^258^. In this study, the researcher loaded oxaliplatin, IR780, and BMS-1 into heat-sensitive liposomes and conjugated folic acid to the OXA prodrug to achieve active tumor targeting. IR780 mediates a photothermal transition under NIR, which releases OXA prodrugs and BMS-1 by inducing a slight thermal effect. PTT in which it can induce the release of endogenous adjuvants such as heat shock proteins and DAMP, thereby enhancing the ICD response. It has been shown to prevent tumor metastasis and neoplasm formation based on the ICD response elicited by the OXA prodrug and the long-term tumor-specific memory response elicited by BMS-1. The synergistic effect initiated by ICB therapy and OXA treatment promotes the maturation of DCs and infiltration of antigen-specific T cells, resulting in long-term control of both primary and distal tumors.

In contrast to other metal applications, most Pt-based nanomaterials can be used as ICD inducers to induce the release of DAMPs. PTT and PDT therapies, on the one hand, can precisely control the release of drugs, thus reducing the incidence of toxic side effects of medications. In contrast, the onset of ICD response can be induced. Therefore, the synergistic use of PTT and PDT is a crucial feature of Pt-based nanomaterials, and the synergistic use of both causes the release of DAMPs and TAAs, which promotes the maturation of APCs and T-cell activation, amplifies anti-tumor immune responses, and enhances the immune response rate. In addition, the clinical use of immunotherapy is limited by inadequate immune activation and response rates. Pt-based drugs can achieve enhanced immune response rates by blocking the interaction between immune checkpoint receptors and their corresponding ligands on tumor cells, promoting infiltrating T cells, and strengthening the immunogenicity and antigenicity of tumor cells. Therefore, the synergistic use of Pt-based drugs with immune checkpoints is promising for clinical application.

### 5.3 Cu-based nanoparticles for immunotherapeutic applications

Cu is a trace element in the human body that is closely associated with various signaling pathways and tumor-related biological behaviors in the body. In humans, Cu promotes physiological activities such as cell proliferation, angiogenesis, and cell migration. Cu(II) and Cu(I) are the two oxidation states of Cu, of which Cu(I) is considered the predominant form in the reduced environment of cellular cytoplasmic lysates. *In vivo*, copper is not only a structural component of a variety of proteins, but also makes up the cofactors of a number of copper-dependent enzymes, such as Cu/Zn superoxide dismutase 1 (SOD1), ceruloplasmin (CP), lysyl oxidase (LOX), cytochrome *c* oxidase (CCO) tyrosinase, and dopamine *β*-hydroxylase (D*β*M)^259^. Maintenance of Cu homeostasis in the body is essential. In the case of copper deficiency, anemia first appears in the clinic, and then connective tissue defects may be involved, and even severe neuropathy in the late stage. If there is a lesion in the Cu transport enzyme, such as a genetic defect in ATPase- ATP7B^260^. Because of impaired ATP7B function, Cu is not transported from hepatocytes to bile and accumulates in the liver and other tissues, leading to acute gastrointestinal symptoms, such as diarrhea, vomiting, and chronic Cu toxicity. Therefore, the balance of Cu is essential for human metabolism.

In addition, Cu is also a dynamic signaling metal and a regulator of metal degradation, for example, Cu-dependent phosphodiesterase 3 B (PDE3B) in lipolysis, mitogen-activated protein kinase kinase 1 (MEK1) and MEK2 in cell growth and proliferation, and the kinases ULK1 and ULK2 in autophagy^261^^,^^262^. Researchers can target essential substances involved in Cu homeostasis to regulate and treat related diseases.

In addition, Cu can cause the onset of death compared to other metals^261^. Cuproptosis is a regulated mode of cell death that differs from the known death mechanisms and is dependent on Cu and mitochondrial respiration. It was found that cells dependent on mitochondrial respiration were nearly 1000-fold more sensitive to copper ions than cells undergoing glycolysis, and the key gene that promotes copper-induced death-FDX1 was identified through a multiplexed CRISPR knockout screen. Cuproptosis occurs through the direct binding of Cu to the lipoylated components of the tricarboxylic acid (TCA) cycle. This leads to the aggregation of lipoylated proteins and subsequent loss of iron-sulfur cluster proteins, resulting in proteotoxic stress and cell death.

However, Cu is more toxic to cells than Mn and Pt. However, copper metals can overcome their toxicity by using nanomaterials so that they are not delivered in a free state. One study confirmed that the half minimal inhibitory concentrations of Cu, silver (Ag), and gold in PC3 and HeLa cell lines were 97.1/85.5, 88.6/78.9, 82.9/66.4 μg/mL, respectively, demonstrating the low toxicity of Cu-based nanomaterials^263^. Cu-based nanomaterials also have the advantages of good biocompatibility, high stability, and photoresponsive properties; therefore, they can be designed for drug delivery carriers. Cu-based nanomaterials are used for tumor imaging in addition to being used as delivery vehicles. Imaging is divided into two main categories: functional (*e.g.*, positron emission tomography and single-photon emission computed tomography) and structural (*e.g.*, X-ray, computed tomography, and magnetic resonance imaging). Because of its radioisotope ^64^Cu, higher near-infrared absorption, and magnetic shift capability, Cu can be used for a wide range of imaging functions instead of a single one for other metals. Examples of these are PET, photoacoustic imaging (PAI), and MRI.

#### 5.3.1 Copper-sized nanoparticles as nanocarriers for immunotherapy

In addition to gold and carbon nanotubes, copper-based nanomaterials have shown good phototherapeutic properties due to their low cytotoxicity, low cost, and specific absorption in the near-infrared range^264^. Cu-based nanomaterials are often designed as delivery carriers because of their excellent biocompatibility, large specific surface area, and photoresponsive properties. For example, one study developed a photoresponsive platform based on semiconducting CuS nanoparticles (CuS NPs) with excellent photothermal conversion properties^265^. The semiconductor nanomaterial chosen for the experiments, CuS, can be used not only as an NIR-triggered photothermal converter for PTT but also as a primary carrier for modifying Cas9 ribonucleoprotein capable of targeting PTPN2 on its surface. It was shown that CuS-RNP@PEI nanoparticle treatment could effectively deplete PTPN2, leading to the enrichment of intratumoral infiltrated CD8^+^ T-lymphocytes in loaded mice, as well as upregulation of the expression levels of IFN-*γ* and TNF-*α* in tumor tissues, which made the tumors more sensitive to immunotherapy. There have also been studies on CuS nano-delivery carriers for PDT therapy to complement ICD efficacy^266^. Nanocomposites were obtained by integrating CuS–MnO_2_ and CaO_2_ NPs. In this case, the CuS nanoparticles not only acted as part of the drug carrier to deliver the drug but also induced the production of ^1^O_2_ by PDT under NIR irradiation (1064 nm) to trigger the ICD response. Moreover, PDT induces oxidative damage to DNA in mitochondria, released in tumor cells, thereby promoting the conversion of tumor-associated macrophages into M1-type macrophages and achieving immunosuppressive TME reconstitution. Thus, Cu-based nanomaterials can be used as delivery carriers to deliver drugs on the one hand and, simultaneously, can be modified on their surfaces for multifunctional antitumor therapy. On the other hand, Cu-based nanomaterials with photothermal conversion properties can also be used for PTT and PDT treatment.

In addition to the CuS described above, which can be used as a drug delivery carrier, Cu-based MOFs can be used for drug delivery. According to their morphology, Cu-MOFs are classified as one-dimensional, two-dimensional, or three-dimensional. Compared to 2D and 3D, 1D is less used in biomedical fields. HKUST-1 (ligand:1,3,5-benzene tricarboxylic acid, denoted as H3BTC) and Cu-TCPP (ligand: tetrakis (4carboxyphenyl) porphyrin, denoted as TCPP) MOFs are the most widely explored three- and two-dimensional Cu-MOFs, respectively. One study used HKUST-1 loaded with NO and assembled it with graphene oxide to obtain nanoparticles that exhibited near-infrared photothermal responsiveness and could release NO molecules in a controlled manner^267^. Owing to its high specific surface area and adjustable pore size, HKUST-1 offers excellent advantages as a storage and transport system for NO. In addition, to facilitate the precise release of NO molecules more profound into the wound, nanoparticles were loaded into porous poly (ethylene glycol) diacrylate microneedles, resulting in microneedles with a porous high surface area and high mechanical stability.

Copper ions are known to significantly enhance the antitumor activity of DSF by chelating the dihydrothiocarbamate ligand (DDC), a metabolite of disulfiram (DSF), to form the Cu(DDC)_2_ complex^268^. However, endogenous copper cannot form enough Cu(DDC)_2_ complexes to kill cancer cells. Therefore, in order to enhance anti-tumor efficacy, copper-based delivery systems have been investigated for the co-delivery of DSF and copper ions. Hou et al.^269^ proposed an *in situ* DSF anti-tumor efficacy trigger system based on Cu-MOFs. The system was an MOF constructed from Cu ions and 2-methylimidazole (2-MI), and DSF was encapsulated in Cu-MOF nanoparticles during MOF formation. The MOF nanoparticles were coated with HA to enhance tumor targeting and biocompatibility. In this system, Cu-MOF acted as a drug delivery carrier and a Cu ion reservoir chelating with DSF to show anticancer activity. Upon reaching the tumor site, the nanoion cells internalize the cells *via* CD44 receptor-mediated endocytosis and are broken down by hyaluronidase in the lysosome and an acidic environment. The catabolism of released DSF and Cu^2+^ can generate toxic DSF/Cu complexes that can kill 4T1 breast cancer cells by binding to the nucleoprotein localization 4 (NPL4) target in the p97 pathway of tumor cells. In summary, Cu-MOFs are usually used as drug delivery carriers in applications and often exert anti-tumor immune activities.

#### 5.3.2 Cu-based nanoparticles for TME-responsive immunotherapy

As mentioned earlier, the TME is a highly heterogeneous environment that actively drives tumor development. Cu ions are more responsive to primary TME states, such as GSH, acidic, and anoxic states than Mn ions are to tumor-associated macrophages and immune checkpoints.

In mammals, GSH and glutathione disulfide (GSSG) are redox pathways mainly used to prevent ROS-induced cellular damage, maintain the stability of thiol-containing enzymes, and preserve cell membrane integrity. However, the rapid proliferation of tumor cells leads to a GSH content in tumor tissues that is approximately 4-fold higher than normal tissues and can reach 0.5–10 mmol/L^270^^,^^271^. Elevated intracellular GSH levels inhibit the clearance and detoxification of harmful substances. This significant difference in GSH levels could be used to construct redox-sensitive nanodrug delivery systems. Since Cu^2+^/Cu has a lower oxidation potential (0.153 eV) than classical Fe, it can be used to build redox-responsive nanocarriers. Cu^2+^, as an oxidant, can react with intracellular GSH and oxidize it to GSSG to eliminate GSH^272^. In addition, the antitumor effect of PDT and immunotherapy can be significantly improved because the carriers of Cu-MOFs have GSH-depleting properties, which can synergize the anticancer effect of ^1^O_2_ produced in PDT^273^^,^^274^. Wang et al.^273^ designed a metal–organic framework (PS@MOF199) of Cu(II) carboxylate loaded with a photosensitizer, which can be used for TME-responsive PDT. To simultaneously achieve activation of the photosensitizer and a significant reduction in intracellular GSH levels, Cu-based MOF-199 with a nanoscale pore size and porous nature, with the metal center Cu (II), was chosen for this study to both activate the photosensitizer and reduce GSH levels. This study confirmed the modulation of intracellular GSH by preparing staining cells using a GSH assay kit.

**Figure 12:**
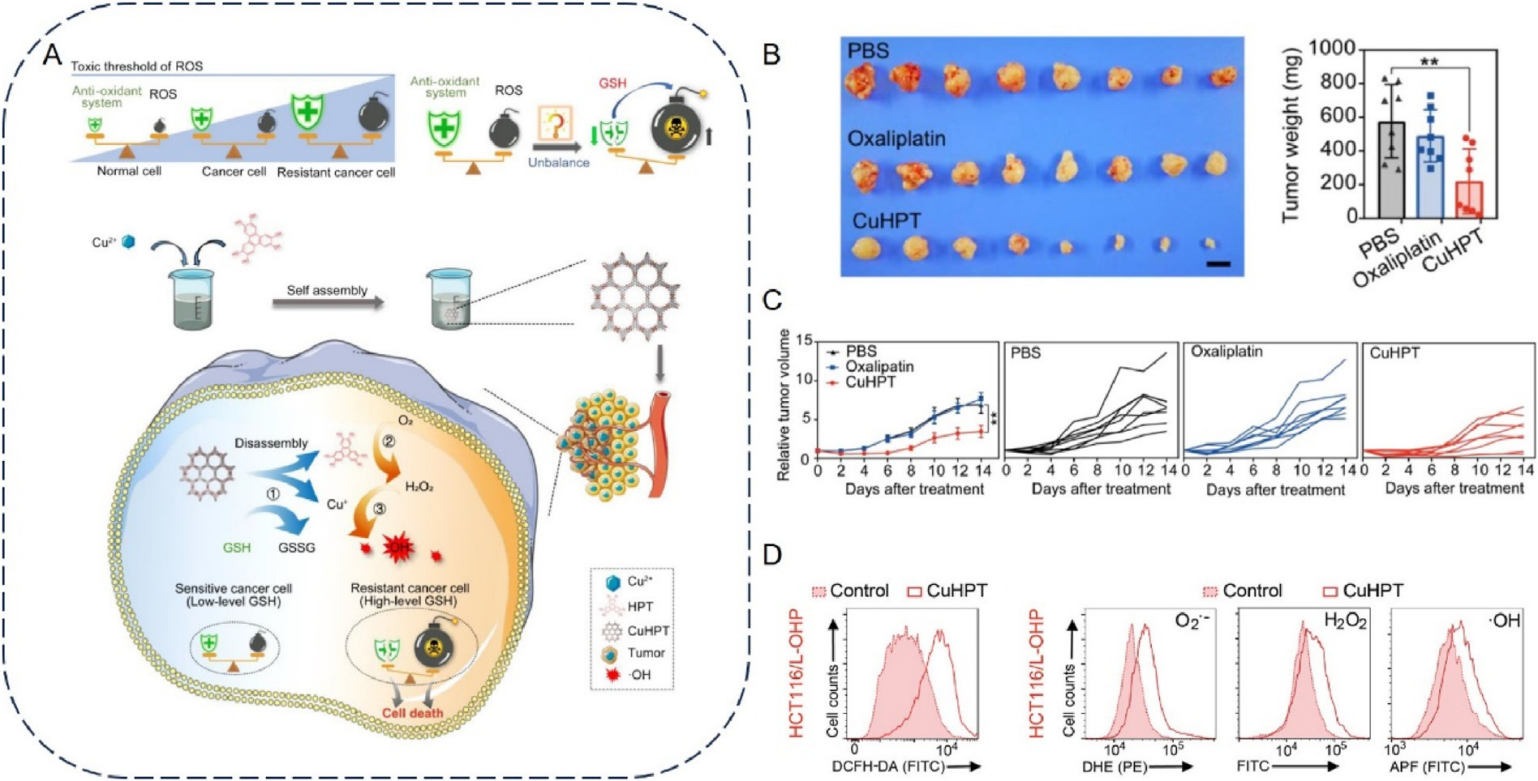
Cu-based metal–organic framework overcomes cancer chemoresistance through systemically disrupting dynamically balanced cellular redox homeostasis. (A) Schematic representation of redox homeostasis in cancer cells and the antitumor mechanism of CuHPT. (B) Image of isolated tumors. Scale bar, 1 cm. (C) Relative tumor volume changes during therapy (mean ± SEM). (D) ROS level in HCT116/L-OHP cells treated with CuHPT. O_2_, H_2_O_2_, and ·OH levels in HCT116/L-OHP cells treated with CuHPT^282^. Reprinted with the permission from Ref. 282. Copyright 2022, American Chemical Society.

Cu(I) is a redox-active metal that promotes the generation of reactive oxygen species (ROS), which in turn activates the proto-oncogene signaling pathway^275^, ^276^, ^277^, ^278^. It has been shown that the Cu(I)-catalyzed Fenton reaction is kinetically more efficient than Fe(II) even in neutral or weakly acidic media (1μ × 10^4^ M/s about 160 times)^279^. After removing intracellular GSH, Cu^2+^ is reduced to Cu^+^, which can undergo a Fenton-like Reaction with intracellular H_2_O_2_ (up to 50–100 μmol/L) in tumor cells to produce toxic ·OH^280^^,^^281^. Although the ability to generate ·OH makes Cu-MOFs an ideal candidate for CDT, CDT's effectiveness largely depends on the concentration of Cu and H_2_O_2_ at the lesion site. When catechol is complexed with Cu ions, an oxidative stress state triggered by GSH, precisely released and maintained by its consumption, is generated, which can increase the concentration of H_2_O_2_ in cancer cells by increasing intracellular ROS. Therefore, a study was conducted to construct self-assembled nanoscale Cu-based metal–organic frameworks (Cu-HPTs) from Cu ions and catechols (Fig. 12A)^282^. The results showed that Cu-HPT could effectively reduce the GSH/GSSG ratio and specifically increase the ROS content in drug-resistant cancer cells by taking advantage of the high GSH level in tumor cells as a “trigger” to activate and maintain the amplification effect of ROS. In the drug-resistant tumor model, the Cu-HPT-treated group showed significantly inhibited tumor growth (62% inhibition) and did not cause systemic toxicity or weight loss (Fig. 12B and C). In addition, ROS levels in mouse tumors were measured using relevant reagents, and the results showed that Cu-HPT significantly increased ROS levels in tumor tissues (Fig. 12D). Moreover, the study's results also showed that the median survival time of the mice was extended from 37.7 to 61.4 days after treatment with Cu-HPT. These results suggest that Cu-HPT can disturb redox homeostasis in drug-resistant cancer cells through multiple pathways, thus promoting the death of cancer cells.

In addition, GSH depletion induces apoptosis in cancer cells in ferroptosis and leads to the inactivation of glutathione peroxidase 4 (GPX4). Meanwhile, GPX4, through its catalytic activity, attenuates lipid peroxidation toxicity and maintains membrane lipid bilayer homeostasis^283^^,^^284^. He et al.^285^ constructed an iron–Cu hybrid MOF containing disulfide-bonded ligands for multimodal imaging and GSH depletion-induced iron-death reactions. In this study, Fe^3+^, Cu^2+^, and GSH-responsive linkers induced GSH depletion, which inactivated GPX4 and induced an iron death response.

In summary, because Cu-based nanomaterials have a lower redox potential, they can be used as oxidants to react with intracellular GSH and oxidize to GSSG to eliminate GSH. After the elimination of GSH, Cu^+^ can undergo a Fenton reaction-like reaction with H_2_O_2_ in tumor cells to produce toxic ·OH. Thus, Cu-based nanomaterials can be designed as redox-sensitive nano drug-delivery systems. In addition, GSH depletion also induces the onset of ferroptosis; therefore, Cu-based nanomaterials can also be used synergistically with ferrous metals to cause the onset of iron death.

#### 5.3.3 Immunotherapy mediated by Cu-sized nanoparticles that modulate copper homeostasis

Cu is an essential cofactor in living organisms; however, it exhibits cytotoxicity when its concentration exceeds the threshold maintained by homeostatic mechanisms. It also exhibits toxicity if its concentration is insufficient. Therefore, it is essential to maintain Cu homeostasis. Cu has been shown to act as an active site cofactor and a dynamic metal signaling and metal-altering regulator that controls and orchestrates biological activity in response to external stimuli^286^^,^^287^.

Cu acts through primary and secondary effects on signaling pathways, both enzymatic and non-enzymatic, which in turn cause cell growth, proliferation, hyperplasia, or tumor formation. This process is called copper proliferation. Cu proliferation-mediated tumor growth may involve a variety of mechanisms, such as increased energy supply, with tumor cells requiring higher levels of Cu than normal cells to meet their energy requirements. Since Cu is a cofactor for Mitochondrially Encoded Cytochrome C Oxidase I (MT–CO1) and Mitochondrially encoded cytochrome *c* oxidase II (MT–CO2), Cu is essential for mitochondrial respiration and ATP production^288^. Thus, Cu signaling can be inhibited in tumor therapy by Cu-selective chelators to inhibit Cu formation; metal ion carriers can also be selected to activate Cu signaling. Of course, other metals, such as iron, can also promote iron-dependent cell proliferation (ferroproliferation) by modulating signaling pathways. Cu hyperplasia is thus an example of the formation of different metal forms.

For example, d-penicillamine, a well-established Cu chelator used to treat Wilson's disease, inhibits the proliferation of gliosarcoma and mesothelioma cells ex vivo in preclinical models^289^^,^^290^. In addition, tetrathiomolybdate (TTM) is a Cu chelator that enhances the efficacy of kinase inhibitor drugs in oncogenic signaling pathways, particularly BRAF-driven MAPK signaling^291^. Hindering Cu-dependent tumor-related processes without compromising healthy tissue function. Tetraethylenepentamine pentahydrochloride is another Cu chelator that promotes ubiquitin-mediated degradation of PD-L1, thereby enhancing antitumor immunity^132^.

In 2022, a team of researchers from Harvard University and the Broad Institute of MIT in the United States discovered for the first time the mechanism by which Cu ions induce cell death, unlike all other known mechanisms of regulated cell death, including apoptosis, iron death, pyroptosis, and programmed necrosis. Therefore, researchers have named this type of cell death-cuprotosis^261^. When copper ions are transported into cells *via* copper ion carriers, excessive accumulation of copper ions in cells dependent on mitochondrial respiration leads to abnormal aggregation of thioctylated proteins and interference with mitochondrial respiration-associated iron-sulphur cluster proteins, causing a proteotoxic stress response that ultimately leads to cell death. The main factors influencing cuproptosis are still unknown, but several metabolic pathways have been demonstrated to influence tumor suppression. First, glycolytic status determines the sensitivity of tumor cells to cuproptosis, and glycolysis inhibitors may potentiate the cuproptosis -mediated inhibition of tumor growth^292^^,^^293^. Second, both Cu deficiency and overload lead to ATP depletion. Hyperactivation of adenosine 5′-monophosphate (AMP)--activated protein kinase (AMPK) increases copper sensitivity in human pancreatic cancer cells^294^. This may be because AMPK mediates the methylation of glyceraldehyde-3-phosphate dehydrogenase and inhibits glycolysis through coactivator-associated arginine methyltransferase 1 (CARM1)^295^. In contrast, early activation of AMPK can trigger mitochondrial unfolded protein responses to mitigate mitochondrial proteotoxic stress and possibly Cuproptosis^296^. Finally, epigenetic mechanisms regulate Cuproptosis^297^. For example, the atypical methyltransferase METTL16 promotes FDX1 accumulation through N6-methyladenosine modification of FDX1 mRNA, which ultimately leads to cancer cell copper death. Taken together, cuproptosis is part of a broader regulatory mechanism of cell death induced by copper ion carriers.

Copper death in cancer cells directly provides new ideas for cancer therapy, and copper death induced by the unexpected binding of Cu to lipoyl proteins becomes a new site of cell death^298^. A similar study showed that the copper mortality score predicts prognosis and characterizes the tumor immune microenvironment in hepatocellular carcinoma^299^. These findings suggest that the death of copper may be an important molecular pathway for improving the efficacy of immune therapy. Because of the above regulatory mechanisms, Prof. Dai Yunlu and Prof. Li Bei at the University of Macau constructed a nano-inducer (PCMs) to trigger mitochondrial autophagy-inhibited cuproptosis proteotoxicity^300^. In this study, PCMs could reduce Cu^2+^ to Cu ^+^ by simple metal-phenol coordination using PEG-polyphenol-Ce6 with phenolic groups. Because of its high affinity for dihydrolipoctamide S-acetyltransferase (DLAT), Cu^+^ can aggregate DLAT for cuproptosis proteotoxic stress and mitochondrial respiratory inhibition. Meanwhile, Ce6 coupled with PCMs can enhance proteotoxic stress by utilizing intracellular oxygen saved by respiratory inhibition. Thus, this study developed a novel approach to improve anticancer immunotherapy by inducing mitochondrial proteotoxicity.

**Figure 13:**
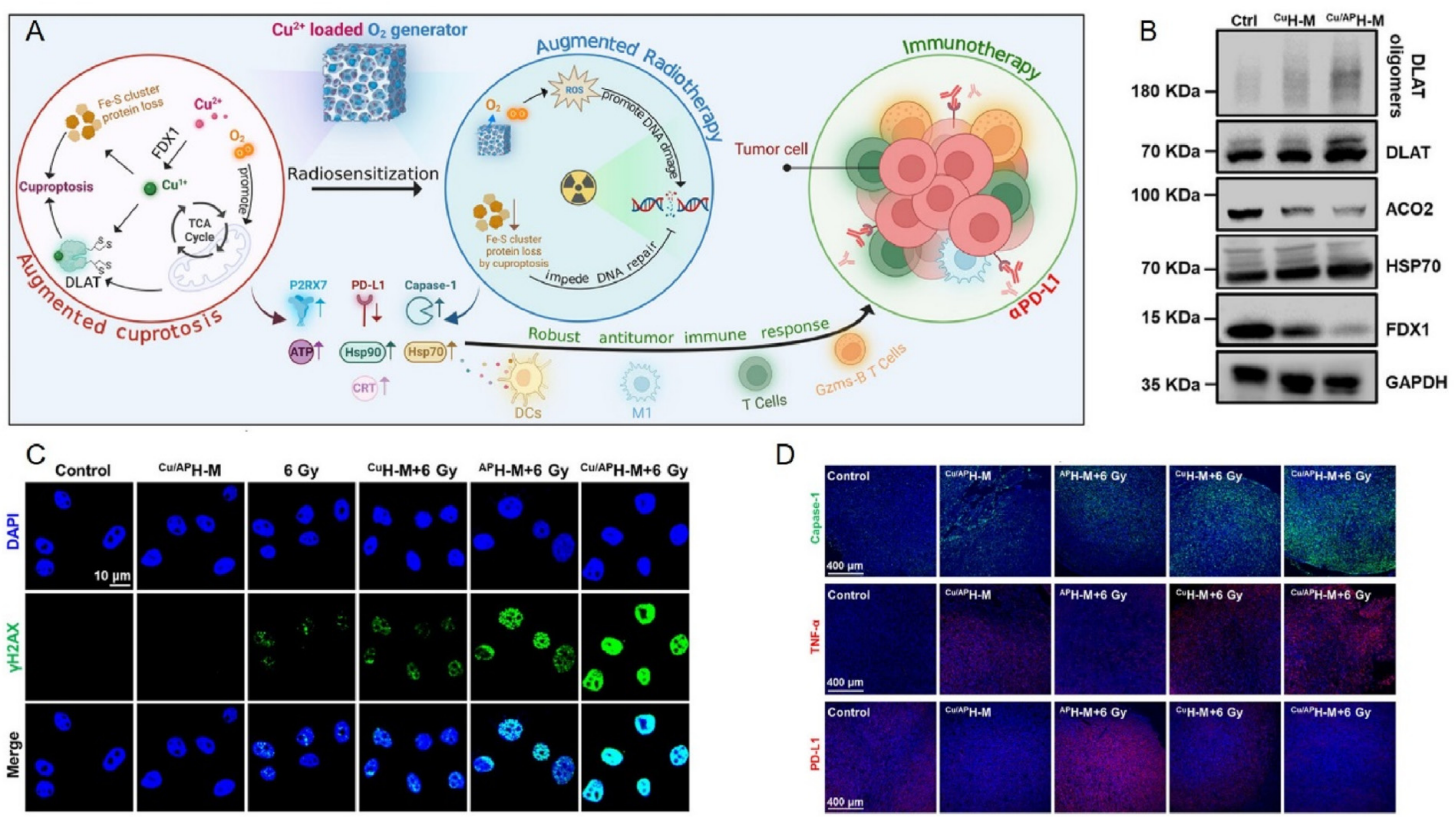
Oxygen generator of Cu ions (^Cu/AP^H-M) for dual modulation of anti-cancer immunotherapy (A) Schematic diagram of ^Cu/AP^H-M oxygen generator dual strengthening both cuproptosis and radiotherapy, synergistic boosting anticancer immunotherapy. (B) Western blot analysis of DLAT oligomers, ACO_2_, HSP70, LIAS, FDX1, and GAPDH expression levels in CT26 cells after being treated with ^Cu/AP^H-M (20 μg/mL), ^Cu/AP^H-M (20 μg/mL). (C) Representative *γ*-H2AX fluorescence images (blue: DAPI; red: *γ*-H2AX). (D) Immunofluorescence slices of Capase-1 (green) expression; immunofluorescence slices of TNF-*α* (red) expression; immunofluorescence slices of PD-L1 (red) expression^301^. Reprinted with the permission from Ref. 301. Copyright 2024, Wiley.

As cuproptosis is a form of cell death dependent on mitochondrial respiration, the level of oxygen within the tumor significantly affects the sensitivity of cells to cuproptosis. In turn, a hypoxic microenvironment exists in solid tumors. Thus, theoretically, alleviation of the hypoxic TME could enhance the toxicity of cuproptosis on tumor cells to some extent. Therefore, a study has constructed an oxygen generator loaded with Cu ions (^Cu/AP^H-M) to regulate the hypoxic microenvironment of tumors and deliver Cu^2+^ (Fig. 13A)^301^. In *in vitro* experiments, flow cytometry verified that ^Cu/AP^H-M could efficiently translocate Cu^2+^ and induce enhanced cuproptosis. In addition, cuproptosis can be improved by inhibiting the production of Fe–S cluster proteins (Fig. 13B). Notably, ^Cu/AP^H-M mediated the occurrence of doubly enhanced cuproptosis while triggering a powerful anti-tumor immune response (*e.g.*, effectively promoting the upregulation and release of ICD-associated proteins and molecules, such as Caspase-1, TNF-*α*, CRT, HSP70, HSP90, ATP) (Fig. 13C and D). Therefore, this study reports the use of cuproptosis in immunotherapy and elucidates the underlying mechanisms, broadening the avenues of cuproptosis in tumor therapy.

In summary, Cu homeostasis is a unique property of Cu relative to other metals. Researchers can trigger cuproptosis or inhibit Cu proliferation by regulating Cu ion homeostasis.

#### 5.3.4 Cu-based nanoparticles as a pluripotent imaging tool for the synergistic treatment of immune disorders

**Figure 14:**
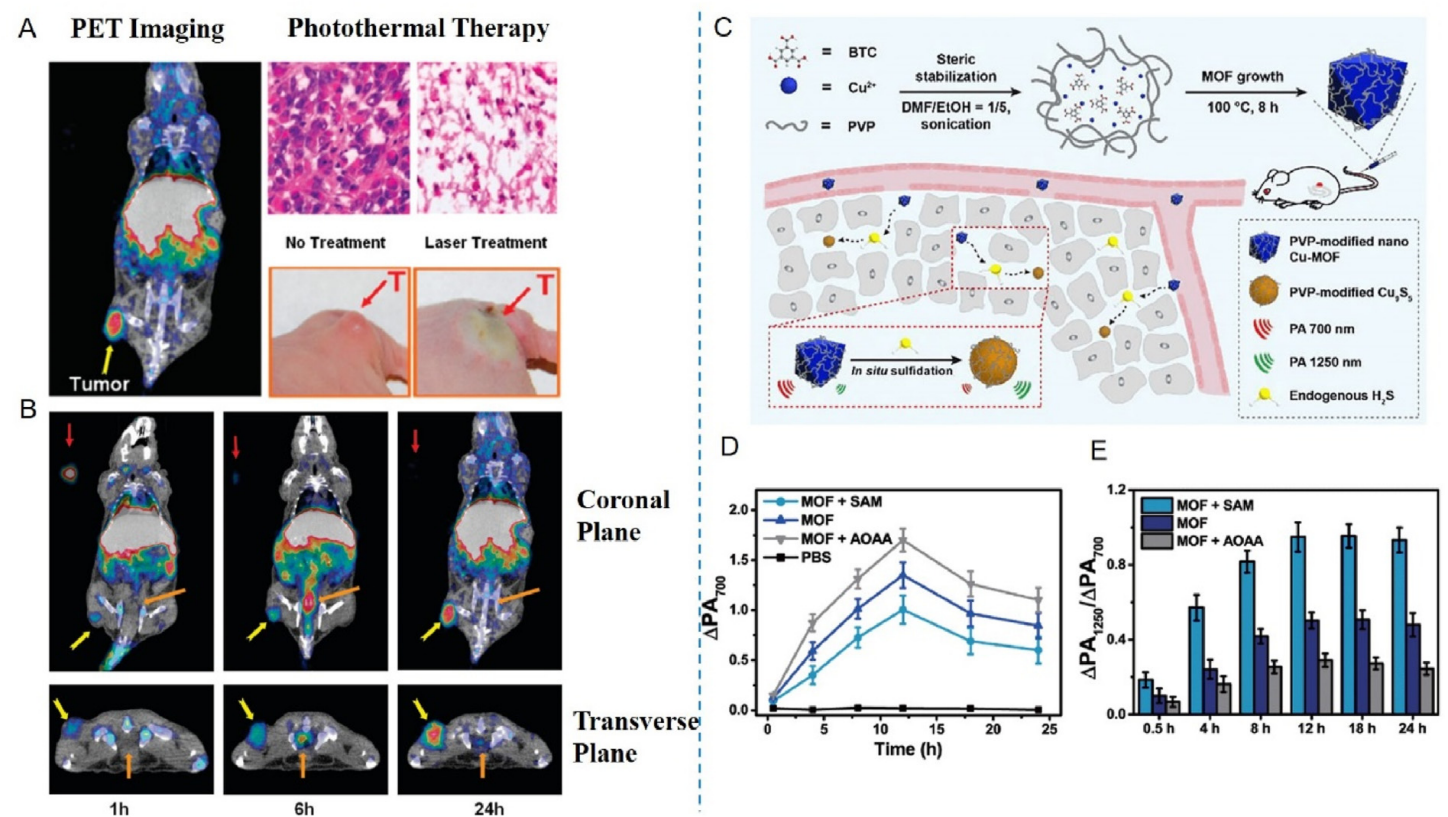
Immunotherapy based on the imaging capabilities of Cu-based nanoparticles. (A) A multifunctional [^64^Cu]CuS nanoparticle platform for simultaneous micro-PET/CT imaging. (B) Micro-PET/CT images of nude mice bearing subcutaneous U87 glioma xenografts acquired at 1, 6, and 24 h after i.v. injection of PEG−[^64^Cu]CuS NPs. Yellow arrow, tumor; orange arrow, bladder; red arrow, standard^302^. Copyright 2010, American Chemical Society. (C) Schematic illustration showing the preparation of nano Cu-MOFs and its application for ratiometric PA imaging of H_2_S and orthotopic colorectal cancer *in vivo*. (D) Temporal PA signal increment in the tumor site post-injection of the nano Cu-MOFs under excitation 700 nm, respectively. (E) Temporal ratiometric PA signal increment (ΔPA_1250_/ΔPA_700_) in the tumor site (color online)^303^. Reprinted with the permission from Ref. 303. Copyright 2020, Science China Chemistry.

PET technology is the use of positron emitter labeled glucose, amino acids, choline and blood flow developers, and other drugs as tracers to show the metabolism, function, blood flow, and other distribution of the organism and the tissue cells of the foci of the disease at the molecular level, and to provide more diagnostic information on physiology and pathology for the clinic. The ^64^Cu isotope is a positron emitter with a half-life of 12.7 h. It exhibits good imaging sensitivity, tissue penetration, and excellent decay properties, which determine its potential application in PET. Given the above properties of Cu, [^64^Cu] CuS nanoparticles were prepared for PET imaging in a simple and fast manner (Fig. 14A)^302^. 24 h after the intravenous injection of PEG-[^64^Cu] CuS nanoparticles, PET images of the whole body of the mouse were obtained, and the mouse's liver, bladder, and tumors were observed at high resolution (Fig. 14B). Additionally, clinical trials have confirmed the use of ^64^Cu in PET. Manlio Cabria and co-workers evaluated 50 prostate patients using ^64^CuCl_2_ PET/CT, ^18^F-choline PET/CT, and multiparametric MRI. The results showed that compared with ^18^F-choline PET/CT, ^64^CuCl_2_ PET/CT had a more appropriate biodistribution *in vivo*, and ^64^CuCl_2_ PET/CT had a higher detection rate.

Photoacoustic Imaging (PAI) technology uses ultrasound generated by the thermal expansion of biological tissues as a carrier to obtain the optical absorption information of the tissues, replacing the photon detection in traditional optical imaging with ultrasound detection, thus avoiding the problem of insufficient depth of penetration due to optical scattering, and breaking through the soft limit of conventional optical imaging (approximately 1 mm), which can achieve a depth of deep tissue photoacoustic imaging of up to 7 cm. Cu-MOF can generate photothermal and photoacoustic materials *in situ* using endogenous stimuli, thus being tumor-specific and measurable deep into the tumor. In one study, CuS nanoparticles generated *in situ* were constructed from Cu-MOF nanoparticles (HKUST-1) and high concentrations of H_2_S in colon cancer for photoacoustic imaging H_2_S probes (Fig. 14C and D)^303^. The probe has high selectivity, a fast kinetic response, and an excellent PA signal for H_2_S in tumors. Meanwhile, the study used the hot-melt method to prepare Cu-MOF, in which the added Polyvinyl pyrrolidone (PVP) can make Cu-MOF achieve the effect that the photoacoustic ratio of PA1250/PA700 increase linearly with the rise in NaHS concentration and reaches chemical equilibrium within the 30 s (Fig. 14E). It was demonstrated that Cu-MOF exhibited efficient H_2_S detection in both *in situ* and subcutaneous colon models, which can be used to monitor endogenous H_2_S levels in organisms and has excellent potential for colon cancer imaging.

The ^64^Cu isotope was a positron emitter with a half-life of 12.7 h. It exhibited good imaging sensitivity and excellent decay properties. Meanwhile, Cu-MOF can generate photothermal and photoacoustic materials *in situ* using endogenous stimuli, which are tumor-specific and measurable deep into the tumor. Given the above properties, Cu-based nanomaterials, unlike other metals, can be used for PET and PAI imaging.

### 5.4 Other immunotherapeutic applications of metallic nanoparticles

#### 5.4.1 Immunotherapeutic applications of iron-based nanoparticles

Iron, an essential trace element, synthesizes hemoglobin in the body, regulates energy metabolism, and maintains normal immune cell function. Since 2012, when a team from Brent Stockwell's lab at Columbia University discovered the phenomenon of ferroptosis, it has been a hot topic of research interest for researchers^304^. Ferroptosis is regulated cell death induced by iron-dependent lipid peroxidation, resulting from dysregulation of the balance between intracellular ROS production and degradation. Ferroptosis inducers act directly or indirectly on glutathione peroxidases (GPXs) through different pathways, leading to reduced cellular antioxidant capacity, ROS accumulation, and, ultimately, oxidative cell death.

A recent study showed that ferroptosis contributes to the antitumor effects of CD8^+^ T cells and influences the efficacy of anti-PD-1/PD-L1 immunotherapy^40^. This suggests some mechanistic crosstalk between immunotherapy and ferroptosis could have a synergistic effect through ferroptosis to promote tumor control. CD8^+^ T cells activated during immunotherapy can reduce the expression levels of the cystine/glutamate transporter protein (System Xc-) subunits SLC3A2 and SLC7A11 by releasing IFN-*γ*, resulting in reduced cystine uptake, enhanced lipid peroxidation, and subsequent ferroptosis^40^.

In addition, there is growing evidence that ferroptosis is associated with resistance to conventional cancer treatments. It has recently been shown that resistance to PD-L1 therapy can be overcome by combining it with a TYR03 receptor tyrosine kinase (RTK) inhibitor, which promotes ferroptosis^305^. Upregulation of TYR03 expression was observed in drug-resistant tumors, where the key to the upregulation of the signaling pathway was an increase in the expression of iron-death genes, such as SLC3A2, which inhibits oncogenic ferroptosis. Researchers at the University of Frankfurt, Germany, then gave a mouse model of colorectal cancer an iron death activator (withaferin A), an immune checkpoint inhibitor (*α*PD-1), and a myeloid-derived suppressive cytostatic inhibitor (SB225002) at the same time^306^. The results showed that this combination therapy significantly reduced liver tumor growth and that it was able to reduce liver metastases caused by metastatic colorectal tumors. This study then demonstrated ferroptosis-induced immune responses under a specific TME, opening up new ideas for treating primary liver tumors and metastases.

However, the clinical use of ferroptosis, characterized by iron-dependent lipid peroxidation, is limited by the potential side effects of exogenous iron. A multivalent ferroptosis agonist (hPPAA18C6) was developed to release endogenous iron and activate ferroptosis^307^. In contrast to natural ferroptosis agonists (*e.g.*, erastin, RSL3, and sorafenib), hPPAA18C6 induces ferroptosis by releasing endogenous iron stored in the natural “iron pool” of the organelle and by depleting GSH through the consumption of benzoquinone produced by the hypoxic cascade decay reaction. In addition, the positively charged primary amine (NH_2_) exposed to hPPAA18C6 acts as an immune adjuvant to promote DC maturation, antigen presentation, and CT8^+^ activation. Therefore, this study provides new therapeutic strategies for tumor treatment through functional polymers in order to hijack endogenous iron and activate the iron death pathway through glutathione.

TAMs mainly exist as M2-type macrophages within tumors. However, M2-type macrophages fail to phagocytose tumor cells and secrete large amounts of TGF-*β*. This attenuates the antitumor capacity of innate immune cells and suppresses the function of CD8^+^ T cells, leading to poor anticancer immunotherapeutic effects mediated by ferroptosis. This attenuates the anti-tumor capacity of innate immune cells and suppresses the function of CD8^+^ T cells, leading to poor anti-cancer immunotherapeutic effects mediated by ferroptosis. Therefore, if M2-type macrophages can be specifically eliminated or reverted to M1-type macrophages, immunosuppressive TME can be alleviated and immunotherapeutic efficacy can be significantly improved. Compared with other metal ions (*e.g.*, Mn ions) that promote the reprogramming of TAMs, the advantage of Fe metals lies in the higher resistance of M1-type macrophages to ferroptosis. This may be due to the overexpression of inducible nitric oxide synthase (iNOS)/NO· by M1-type macrophages, which detoxify ferritin deposits^308^^,^^309^. Thus, immunosuppression can be alleviated by eliminating M2-type macrophages *via* induction of macrophage ferroptosis.

**Figure 15:**
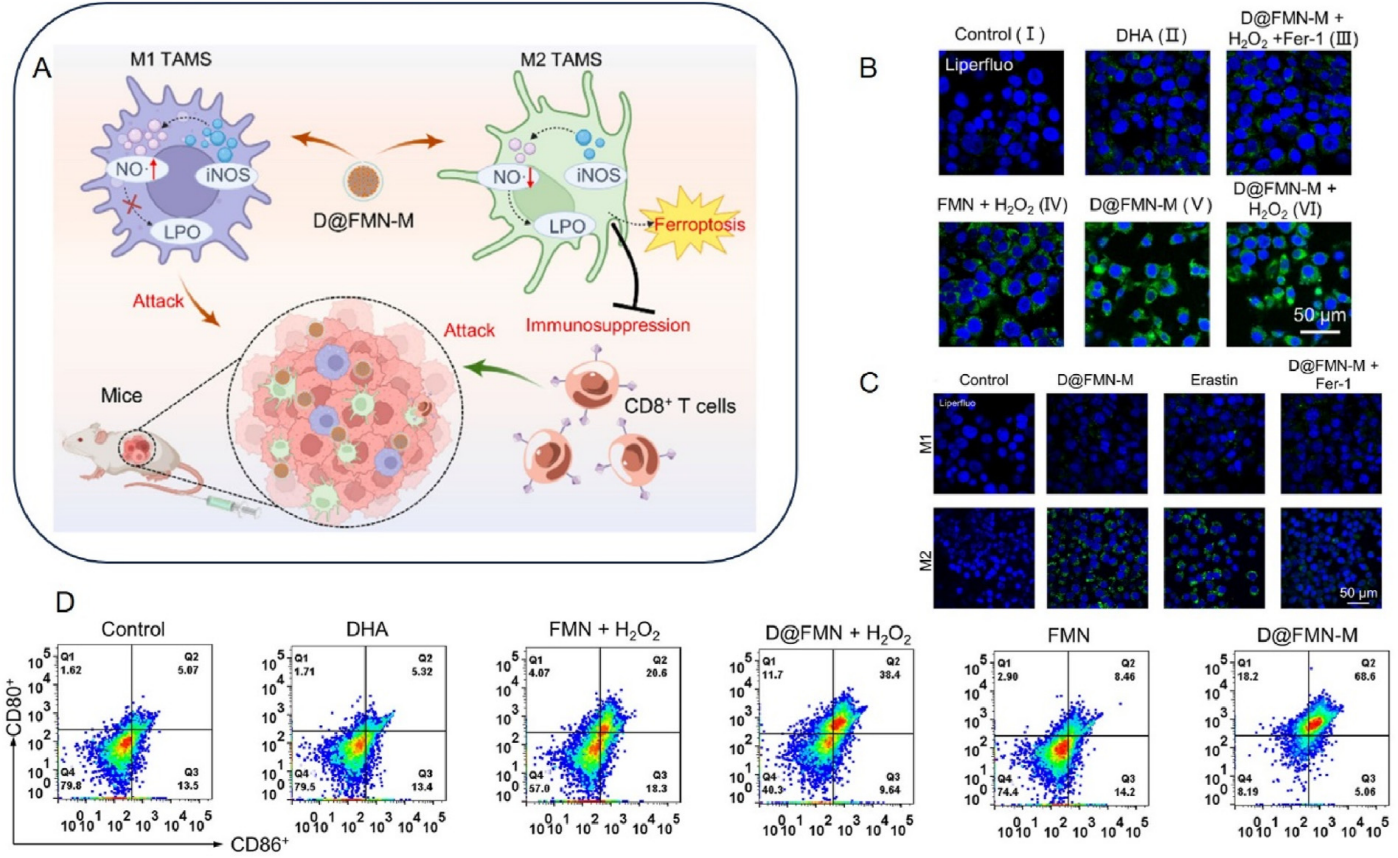
Biomimetic inducer enabled dual ferroptosis of tumor and M2-type macrophages for enhanced cancer immunotherapy. (A) Schematics of D@FMN-M enhanced tumor ferroptosis immunotherapy by inducing dual ferroptosis of tumor cells and M2 TAMs. (B) CLSM images of Liperfluo stained 4T1 cells after different treatments. (C) CLSM images of Liperfluo-stained M1 and M2 macrophages after various treatments. Scale bar = 50 μm. (D) Flow cytometry analysis of the anti-CD11c/CD80/CD86 stained BMDCs after different treatments^310^. Reprinted with the permission from Ref. 310. Copyright 2023, Elsevier.

A study developed a novel bionic ferroptosis inducer (D@FMN-M) for modulating the TME to enhance tumor ferroptosis immunotherapy (Fig. 15A)^310^. D@FMN-M undergoes biodegradation in the acidic environment of tumors, releasing dihydroartemisinin (DHA) and Fe^3+^. Among them, Fe^3+^ can, in turn, be reduced to Fe^2+^ by the abundant GSH in the tumor, thus triggering the Fenton-like reaction and Fe^2+^-DHA reaction, which in turn promotes the generation of a large number of free radicals to induce M2-type TAMs and induce iron death in tumor cells (Fig. 15B). Notably, ferroptosis in tumor cells is further enhanced by an imbalance in intracellular redox homeostasis due to the depletion of GSH within the tumor. Moreover, during the process of ferroptosis, tumor cells release large amounts of TAAs, thereby increasing the immunogenicity of the tumor (Fig. 15D). The experimental results showed that under ferroptosis stimulation, ferroptosis-sensitive M2-type TAMs would be stress-damaged or gradually domesticated into ferroptosis-tolerant M1-type TAMs, thus contributing to the normalization of TME and enhancement of the immunotherapeutic effect of tumor ferroptosis (Fig. 15C). This study provides a new strategy for ferroptosis-immunotherapy of solid tumors.

Compared to other metals, iron also has the property of being magnetic and can be used in tumor therapy^15^. When exposed to high-frequency (100–700 kHz) alternating magnetic fields (AMF), iron oxide nanoparticles can induce magnetic hyperthermia (MH), which leads to apoptosis and cell cycle changes. MHT is a thermotherapy method based on AMF that uses magnetic particles as a heating medium^311^. It has been shown that combining two thermal modes (dual action of MHT and PTT) in a single magnetic plasma object is another suitable and effective thermal mode for cancer therapy in nanotechnology^312^^,^^313^. Therefore, Espinosa and Javier jointly reported Janus magnetic plasma nanoparticles based on gold nanoparticles and iron oxide nanorods as efficient therapeutic nanoheaters^314^. This study explored using Janus gold-iron oxide nanoparticles in MHT and PTT thermal modes alone or in combination by setting different parametric conditions, such as nanoparticle design and nanoparticle concentration. Moreover, when MHT was combined with PTT, it exhibited synergistic cytotoxicity and killed all the cancer cells (cell survival <5%). After systemic intravenous injection, nanoparticles at the tumor site increased significantly under the effect of the magnets. Thus, Janus magnetic plasmonic nanoparticles can be used as an effective localized therapeutic tool for cancer nano therapy based on blood transport.

#### 5.4.2 Zn-based nanoparticles for immunotherapeutic applications

Zn is the second most abundant transition metal element in living organisms, and free Zn ions act as a signal to regulate immune cell functions, such as macrophage phagocytosis, DC maturation, and T cell activation. In recent years, Zn-based nanomaterials have been developed extensively for immunotherapy. It has been shown that excess Zn ions in tumor cells can induce ROS production *via* two pathways: leakage of the aerobic respiratory electron chain and oxidation of nicotinamide adenine dinucleotide phosphate (NADPH) by NADPH oxidase 1 Gene (NOX1) in mitochondria^315^. Excessive accumulation of ROS and the resulting mtDNA damage activates multiple signaling pathways, inducing high levels of interferon and cytokine production. In addition, excessive Zn ion accumulation induces cellular pyrokinesis *via* two pathways: the typical pathway dependent on caspase-1/GSDMD and the alternative path dependent on caspase-3/GSDME^83^. The onset of cellular pyroptosis leads to exposure to many DAMPs, which triggers a powerful systemic anti-tumor immune response.

Given the above immunomodulatory effects of Zn, one study constructed a nano-agonist with tumor-specific and NIR-enhanced catabolic properties (DZ@A7) based on MOFs for photodynamic metal immunotherapy and STING activation^316^. Under NIR irradiation, the engineered nano-agonist generates ROS targeting mitochondria to specifically release mtDNA and inhibit the repair of nuclear DNA *via* a depletion-responsive drug. Oxidized tumor mtDNA acts as an endogenous DAMP to activate the cGAS–STING pathway. Moreover, Zn^2+^ overload in tumor cells accelerated by NIR light could also further enhance the enzymatic activity of cGAS through the metal immunity effect. The synergistic enhancement of the cGAS–STING pathway triggered by this engineered nano-agonist activates the immune effect, which promotes the infiltration of CT8^+^ and the maturation of DC and establishes long-term anti-tumor immune memory while eliminating the primary tumor.

**Figure 16:**
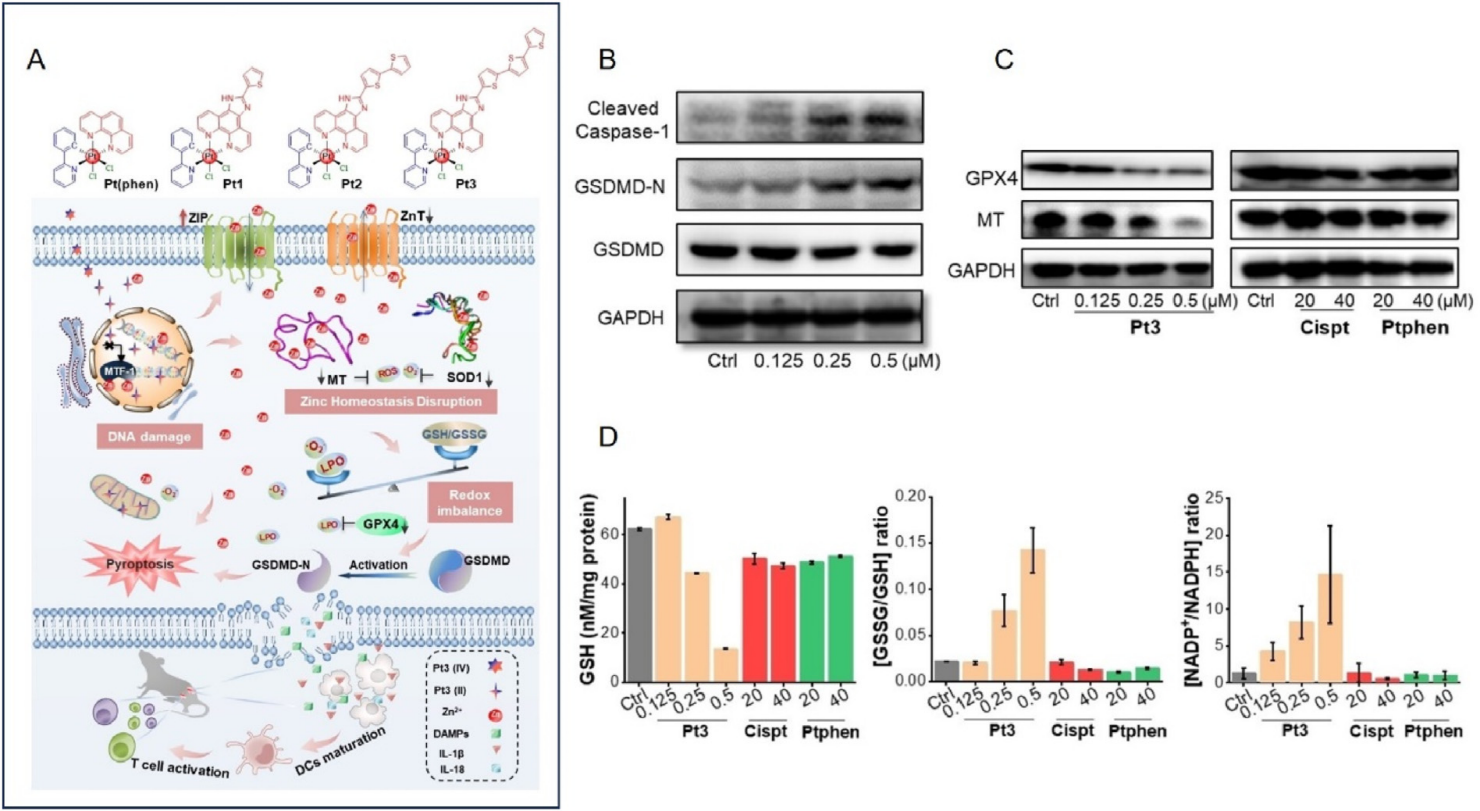
Disruption of Zn homeostasis by a novel Pt (IV)-terthiophene complex for antitumor immunity. (A) The schematic structures and proposed mechanisms of newly developed Pt (IV)-terthiophene complexes that activate antitumor immunity by simultaneous disruption of Zn and redox homeostasis. (B) Western blot analysis of cleaved caspase-1, GSDMD-N fragment, GSDMD-full length, and GADPH expression in MDA-MB-231 cells upon 12 h treatment with compound Pt3. (C) Western blot of GPX4, MT, and GADPH expression in MDA-MB-231 cells. Cells were treated with Pt3, Cispt, and Ptphen (as indicated) for 12 h before analysis. (D) Impact of Pt3, Cispt, and Ptphen at the indicated concentrations on cellular GSH. Impact of Pt3, Cispt, and Ptphen on the ratios of cellular GSSG/GSH and NADP/NADPH^317^. Reprinted with the permission from Ref. 317. Copyright 2022, Wiley.

Despite the critical importance of Zn homeostasis in human immunomodulation, there are few reports on antitumor compounds capable of triggering Zn^2+^-mediated immune responses. As a result, Mao's group^317^ at Sun Yat-sen University doped organic immunomodulatory molecules as axial ligands into Pt (IV) complexes, constructing novel cyclometallated Pt (IV)-trithiophene complexes (Fig. 16A). Although the complex does not contain Zn metal, it has a moderately strong affinity for Zn ions. It can induce aberrant transcription and expression levels of Zn-regulated proteins (*e.g.*, the proteins responsible for Zn transport and exocytosis, ZIP/ZnT, metallothionein MT, and Zn-sensing element-binding transcription factor MTF-1) in cells, which leads to the overaccumulation of Zn^2+^ in the cytoplasm and redox imbalances, including the downregulation of Cu/Zn superoxide dismutase (SOD1), over-accumulation of superoxide anions and lipid peroxides, and GPX4 inactivation (Fig. 16C and D). Furthermore, the results showed that disruption of Zn homeostasis and redox homeostasis by Pt (IV)-trithiophene complexes was accompanied by activation of gasdermin-D-mediated cellular pyrokinesis and concomitant cytoskeletal remodeling, which resulted in nanomolar cytotoxicity (∼0.11 μmol/L) against triple-negative breast cancer cells MDA-MB-231 (Fig. 16B). Ultimately, a powerful immune response is induced, and the released pro-inflammatory cytokines promote DC maturation and T-cell infiltration, eliminating not only primary tumors *in vivo* but also distant tumors.

#### 5.4.3 Ca-based nanoparticles for immunotherapeutic applications

Recent studies have found that Ca ions are also involved in immune signaling pathways. Chronic inflammation and persistent infections lead to malignancy, in which some inflammatory vesicles, including NLRP3, induce fluctuations in cell signaling (*e.g.*, increase in expression of DAMPs). NLRP3 inflammatory vesicles can induce the release of pro-inflammatory cytokines IL-1*β* and IL-18 on top of caspase-1, which has been implicated as a promoter of cancer progression, invasion, and metastasis. Mitochondria can take up Ca^2+^ *via* the mitochondrial calcium uniporter (MCU), which enhances phagocytosis-dependent NLRP3 inflammatory responses, thus enhancing IL-1*β* release. IL-1*β* release can, in turn, exacerbate tissue damage through inefficient inflammatory pathways induced by mitochondrial DAMPs, thereby promoting oncological diseases. Furthermore, the Ca channel STIM1 can modulate the cGAS–STING pathway by sequestering STING in the endoplasmic reticulum, suggesting that Ca homeostasis plays an immunomodulatory role in anti-DNA viruses.

**Figure 17:**
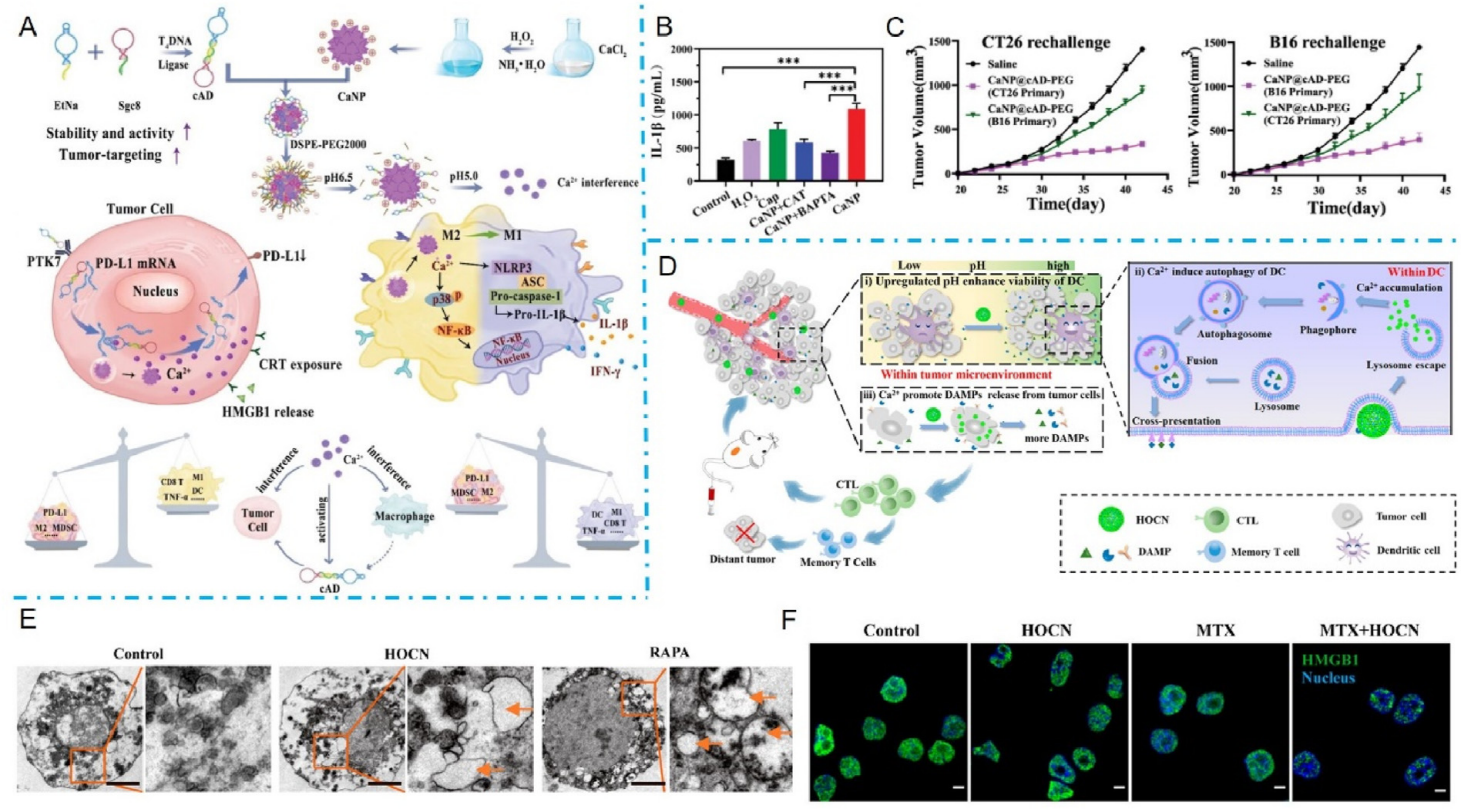
Ca-based nanoparticles for multi-functional applications in the field of immunotherapy. (A) Schematic diagram of bioactive nanoparticles *via* Ca^2+^interference mediate TME reprogramming and specific PD-L1 depletion for boosting cancer immunotherapy. (B) IL-1*β* levels in the supernatant of BMDM-M2 after treatment with H_2_O_2_(0.7 μm), Cap (70 μg/mL), CaNP (35 μg/mL), CaNP + CAT, and CaNP + BAPTA for 48 h (CaNP: 35 μg/mL, CAT: 10 μg/mL, BAPTA: 10 μm) (*n* = 4). (C) Growth curves of the s.c. rechallenge CT26 or B16 tumors in the different groups (*n* = 5)^319^. Reprinted with the permission from Ref. 319. Copyright 2020, American Chemical Society. (D) Schematic diagram of HOCN disruption of multiple barriers in antigen cross-presentation of DCs for enhanced mitoxantrone (MTX)-mediated chemo-immunotherapy. (E) Bio-TEM images of DCs with different treatments for 6 h; orange arrows: vacuoles following autophagy. Scale bar: 2 μm. (F) CLSM images and quantitative examination of HMGB1 release after receiving different treatments for 12 h; scale bar: 10 μm^318^. Reprinted with the permission from Ref. 318. Copyright 2022, Wiley.

Therefore, one study designed a Ca-dependent DNAzyme (cAD) that efficiently shears PD-L1 mRNA using Ca peroxide nanoparticles (CaNPs) as a carrier (Fig. 17A)^318^. It was also loaded by electrostatic adsorption, and DSPE-PEG2000 was modified on its surface, constructing a Ca-regulated gene-drug delivery system (CaNP@cAD-PEG) with TME remodeling properties. It has been shown that after uptake by TAMs, CaNP activates various inflammation-related signaling pathways (*e.g.*, MAPK signaling pathway, NF-*κ*B signaling pathway) as well as NLRP3 inflammasome through “Ca interference” and promotes macrophage phenotypic transformation (Fig. 17B). Meanwhile, by inducing Ca overload in tumor cells, “Ca interference” can promote ICD and further enhance the immunogenicity of tumor cells (Fig. 17C). Thus, the nanoformulation can increase free intracellular Ca ions to modulate immune cell phenotype and function, providing a new way to improve the tumor immunosuppressive microenvironment. The construction of a simple and versatile Ca ion nanogenerator to disrupt autophagy inhibitory conditions within DCs to enrich DAMPs, thereby attenuating acidity in the TME, has also been investigated (Fig. 17D)^319^. Following chemotherapy, honeycomb CaCO_3_ nanoparticles can preferentially accumulate within the tumor and exhibit multifunctionality, thereby breaking the multiple barriers to antigen cross-presentation in DCs. One of the functions is that the nanoparticles can restore DC cell activity by reducing acidity within the tumor. The second function is that the nanoparticles can generate Ca^2+^ inside the cell to disrupt the autophagy inhibition conditions of DCs (Fig. 17E). Function three is that the nanoparticles also promote the maturation of DCs through the Ca^2+^ overload-mediated release of DAMP from tumor cells (Fig. 17F).

#### 5.4.4 Gold-based nanoparticles for immunotherapeutic applications

It has been shown that gold compounds act directly on tumor cells and immune cells and affect the expression of cell adhesion molecules on endothelial cells. Some studies have explored the effect of morphological differences on the activation of immune pathways by preparing AuNPs of different sizes^44^. In this study, gold nanoparticles containing 4.5, 13, 30, and 70 nm were prepared, and their inhibition of autophagy was explored. It was found that ultra-small-sized (<10 nm) gold nanoparticles can directly penetrate cell membranes, promote ROS generation, target the autophagy protein LC3, and promote its degradation. Simultaneously, proteasomal degradation occurs in an endocytosis-independent manner, activating the NLRP3 inflammasome to promote Caspase-1 maturation and IL-1*β* production. In contrast, larger-sized AuNPs (>10 nm) can be taken up *via* energy-dependent endocytosis, thereby triggering the NF-*κ*B signaling pathway.

Gold has unique properties compared to other metals, including a high photothermal conversion efficiency and localized surface plasmon resonance (LSPR)^45^^,^^320^^,^^321^. Owing to these properties, AuNPs can be used for PTT and PDT. When the size of gold nanoparticles is reduced below 50 nm, the conduction electrons in the d-orbitals of gold atoms undergo quantization. When the wavelength of the incident light is larger than the size of the gold nanoparticles, the free electrons in the gold nanoparticles are polarised by the electric field, resulting in collective electron oscillations. Negative and positive charges are concentrated in opposite regions of the gold nanoparticle surface, resulting in a change in the net charge at the boundary and a significant increase in the electromagnetic field in the vicinity. As a result, AuNPs with different sizes and shapes of dielectric constants undergo absorption and scattering, resulting in different optical features. Zhang et al.^322^ designed a dumbbell-shaped gold nanostructure to activate immune responses and inhibit tumor growth and metastasis. In this study, to transfer the local LSPR of AuNPs to the NIR-II window, the researchers adjusted the sequence and concentration of the nuclide-targeting DNA template in the AuNPs. The results showed that dumbbell-type nanostructures have better thermal stability, photoacoustic signal (PA), and photothermal conversion efficiency (84.9%) than conventional gold nanostructures. Moreover, after PTT, gold nanostructures can induce more cellular and humoral immune markers (*e.g.*, TNF-*α* and IL-6) under laser light, activating a strong antitumor immune response.

**Figure 18:**
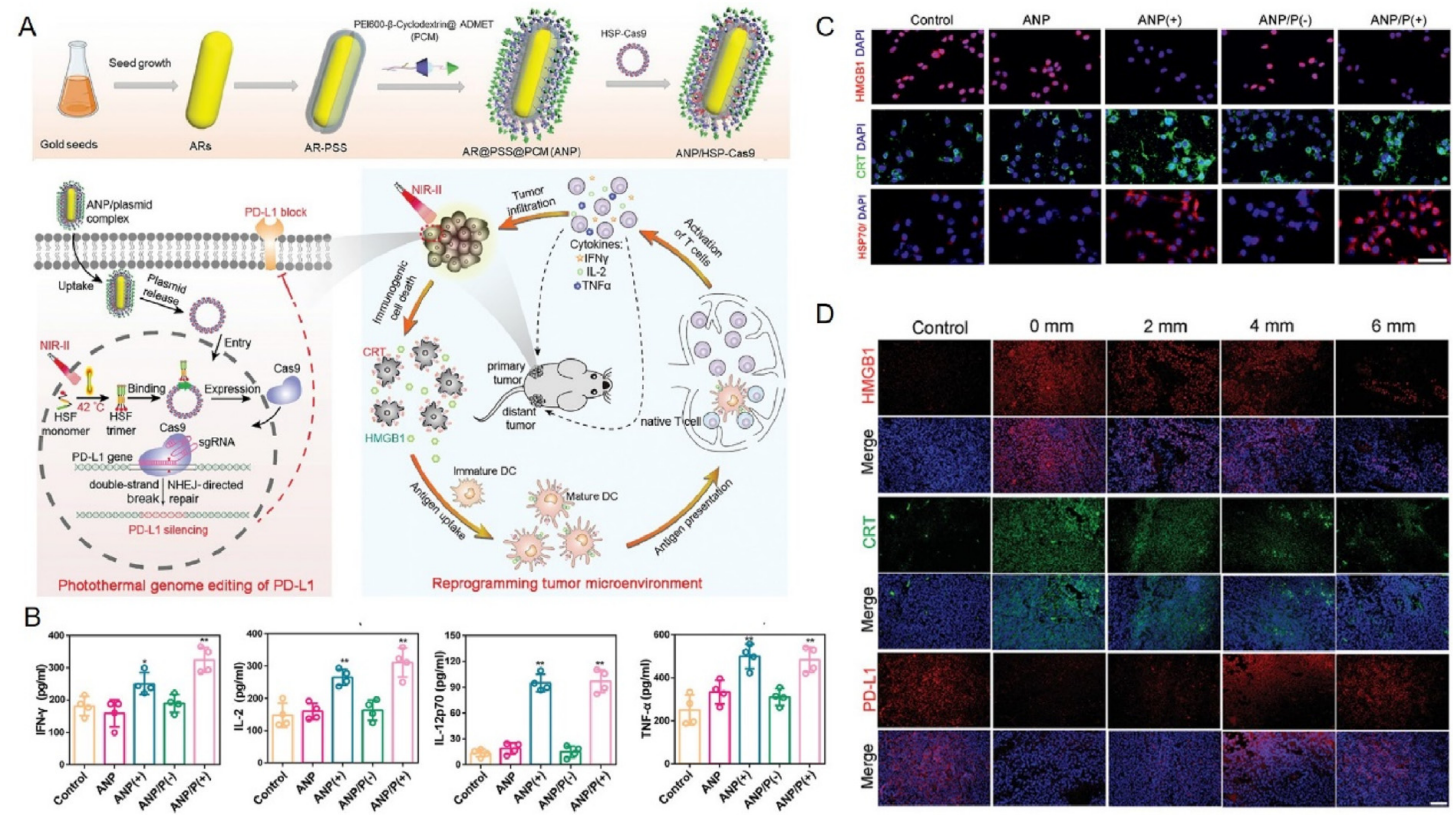
Gold nanocarrier-based delivery capabilities for immunotherapy. (A) Schematic illustration of the photothermal genome-editing strategy for cancer immunotherapy. (B) Contents of the IFN-*γ*, IL-2, IL-12p70, and TNF-*α* in primary tumor tissues after the indicated treatment on day 21. Mean ± SD, *n* = 4. Student's *t*-test, ∗*P* < 0.05; ∗∗*P* < 0.01 *versus* control. (C) Immunofluorescence analysis of HMGB1 (red), CRT (green), and HSP70 (red) expression after the above-indicated treatment. Scale bar: 20 μm. (D) Immunofluorescence analysis of HMGB1 (red), CRT (Green), and PD-L1 (red) protein expression from tumor tissue at different tumor depths. Scale bar: 100 μm^323^. Reprinted with the permission from Ref. 323. Copyright 2021, Wiley.

Ping Yuan's team at Zhejiang University designed a photothermal genome editing strategy based on supramolecular cationic gold nanorods to improve ICB therapy (Fig. 18A)^323^. In this study, a CRISPR/Cas9 plasmid loaded with a heat-inducible promoter (HSP) and delivered efficiently into cells *via* supramolecular cationic gold nanorods. Under NIR-II irradiation, transcriptional activation of Cas9 and a single guide RNA (sgRNA) targeting PD-L1 can then be induced, thus enabling precise editing of the PD-L1 genome (Fig. 18B). Thus, the supramolecular cationic gold nanorods in this study not only acted as carriers to deliver CRISPR/Cas9 targeting PD-L1 but also captured NIR-II light and converted it into mild hyperthermia to induce ICD and Cas9 gene expression (Fig. 18C and D).

Gold nanoparticles also have excellent X-ray absorption coefficients, which can increase energy accumulation by producing photoelectric absorption and the Compton effect, and can therefore be used as sensitizers for radiation therapy. Qin et al.^324^ reported a study on using cell-derived bio-gold nanoparticles to sensitize radiotherapy and enhance tumor immune responses in 2021. This study is based on a cellular reactor of tumor cells to generate gold nanoparticles. The study results showed that the CD8, CD8*α*^+^, and DC numbers, mainly responsible for antigen phagocytosis and cross-presentation, significantly increased in the combination-treated mice with tumor-draining lymph nodes (TDLN). This study also demonstrated the feasibility and efficacy of radiosensitization of cell-derived AuNPs in treating *in situ* breast cancer.

#### 5.4.5 Silver-based nanoparticles for immunotherapeutic applications

Silver nanoparticles (AgNPs) are known to be potent antimicrobial agents, but their specific antimicrobial mechanisms are still not fully understood. Emphasizing an antimicrobial effect centered on silver ions, Durán et al.^46^ showed that silver ions impair DNA replication and that the respiratory electron transport chain decouples from oxidative phosphorylation, thereby inhibiting the permeability of respiratory chain enzymes. Marambio-Jones and Hoek first summarized three mechanisms of the antimicrobial activity of AgNPs: a) uptake of free Ag ions impairs ATP production and DNA replication; b) AgNPs and Ag ions can generate ROS; and c) AgNPs directly disrupt cell membranes. Given that AgNPs have antimicrobial activity, studies have been conducted on their use in colony modulation to enhance anti-tumor immune responses. Xianzheng Zhang's team at Wuhan University then designed an AgNP-based hydrogel for modulating the oral microbiota to enhance immunotherapy^325^. In previous work, researchers found that *Streptococcus* digestiveis could activate the immune system and improve the prognosis of patients with oral squamous cell carcinoma (OSCC). The authors then screened a range of nanomaterials (containing AgNPs, AuNPs, Fe_3_O_4_ nanoparticles, cellulose nanoparticles, graphene nanosheets, ZnO nanoparticles, etc.) They found that AgNPs inhibited the proliferation of other bacteria but allowed *Streptococcus* digestiveis to increase. The researchers then prepared Agel hydrogels (Agel), which can adhere to the oral mucosa, using Schiff base-mediated tissue adhesion hydrogels made from chitosan and polyaldehyde dextran. These findings also show that anaerobically digested streptococci can induce an anti-cancer immune response and inhibit tumor growth only if the oral microbiota is regulated by Agel. In addition, researchers found that synergizing a formulation containing two components (Agel and exogenous *Streptococcus* digestus) with an anti-PD-1 antibody significantly prolonged survival in model mice.

Only a few studies on AgNPs as antibacterial biomaterials have elaborated on their effects on the biological properties of neutrophils^326^, ^327^, ^328^, and few have systematically elaborated on the mechanisms of AgNP's interactions with bacteria and neutrophils. Therefore, Huang et al.^329^ synthesized AgNPs using a polyol method and designed a blood-AgNPs bacterial co-culture model and ROS assay to evaluate the effects of AgNPs on neutrophil-mediated phagocytosis and ROS production. The results showed that the number of neutrophils recruited to the site of inflammation and the expression of pro-inflammatory factors (TNF-*α* and IL-6) were reduced when AgNPs were applied *in vivo*, suggesting that *in vivo* antimicrobial activity was significantly reduced. In addition, the results of an *in vitro* model constructed to mimic the *in vivo* environment showed that AgNPs suppressed the antimicrobial capacity of the natural immune system, which was further confirmed by the reduced ability of Polymorphonuclear Neutrophils (PMNs) to phagocytose bacteria. AgNPs significantly inhibited the ability of neutrophils to produce ROS, superoxide anions, and PMN migration. Therefore, in the natural immune system, AgNPs may be suggestive of foreign microbial invasion and can actively enhance the antimicrobial efficacy of the innate immune system for the development of antimicrobial biomaterials.

#### 5.4.6 Rare metallic nanoparticles for immunotherapeutic applications

**Figure 19:**
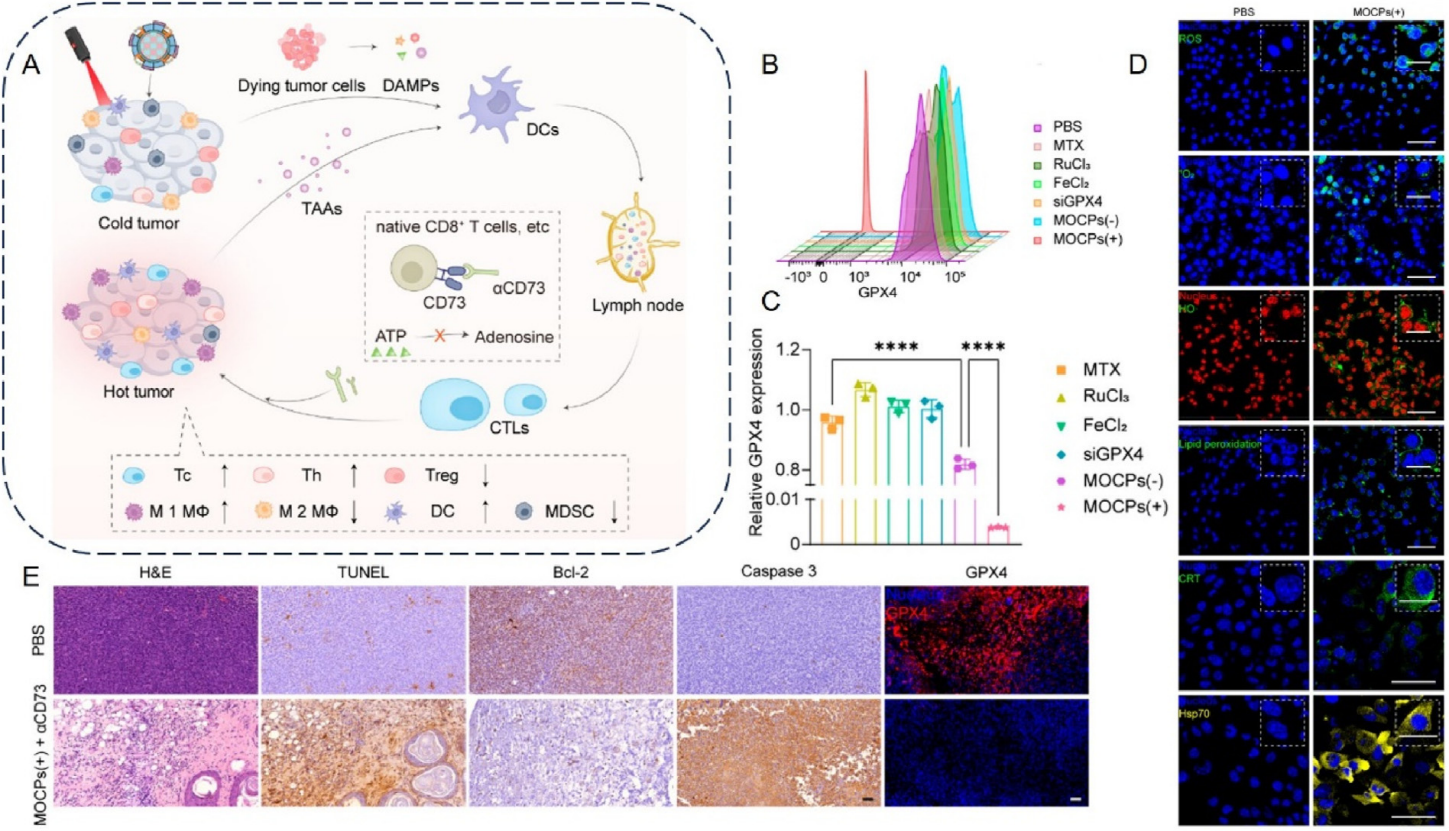
Precise amplification of intracellular oxidative storms by metal–organic coordination polymers to enhance anti-cancer immunity. (A) Construction and characterization of oxidative storm inducers, MOCPs. (B) Flow cytometry showing intracellular GPX4 expression in live 4T1 cells after incubation with free drugs or MOCPs without or with irradiation. (C) After treatment, histograms of GPX4 silencing in live 4T1 cells (*n* = 3). (D) CLSM images (Inset: a high magnification image, scale bar = 20 μm) showing generation of ROS, ^1^O_2_, HO·, distribution of lipid peroxidation, CRT translocation to the outer plasma membrane, and Hsp70 exposure to membrane surface in 4T1 cells after MOCPs (+) treatment. Scale bar = 50 μm. (E) *Ex vivo* tumor sections with HE, TUNEL, IHC, and IF staining showing tumor apoptosis and GXP4 knockdown. Scale bar = 50 μm^332^. Reprinted with the permission from Ref. 323. Copyright 2023, American Chemical Society.

With the rise in metal immunotherapy, the use of metallodrugs in cancer immunotherapy has attracted much attention. In addition to the metallodrugs reviewed above, many have unique immunotherapy applications. For example, the abundance of laser states and tunable photophysical chemistry of ruthenium (Ru) complexes can provide excellent measures for developing metal-based photosensitizers^330^^,^^331^. Therefore, Li et al.^332^ designed a complex photoactive metal–organic coordination polymer (Fig. 19A). These polymers are photo-controlled metal–organic coordination polymers of cytotoxic succinic acid, photosensitive Ru(BIQ)-HDBB, a high ferrous density core, and siGPX4 on the shell. Under local 670 nm LED irradiation, Ru (BIQ)-HDBB can undergo photolysis and produce ^1^O_2_ for PDT (Fig. 19B–E). A Phase II clinical trial uses TLD-1433 as a photosensitizer for treating non-muscle invasive bladder cancer^333^. It has been shown that IR780 is an ideal candidate for PA and fluorescence (FL) imaging for guiding therapy or monitoring^185^^,^^186^. Based on the above theory, Huang et al.^334^ designed a dual immunosuppression strategy, by loading immunomodulators and photosensitizers (IR780) in nanoliposomes (Nano-IR-Sb@Lip). In order to achieve PD-1/PD-L1 checkpoint blockade and TGF-*β* pathway inhibition, it was also combined with PTT therapy for efficient treatment of primary and metastatic tumors. IR780 endows Nano-IR-Sb@Lip with the ability to accumulate and deeply penetrate tumor tissues selectively^335^, ^336^, ^337^. At the same time, internalized IR780 can be preferentially retained in mitochondria, enhancing PPT therapy's efficacy^338^^,^^339^.

## 6 Future prospects

The latest applications of metallic nanoparticles in immunotherapy are reviewed in this article. The multiple functions of metallic nanoparticles in immunotherapy are highlighted in the article. For example, since Mn ions activate the cGAS–STING pathway, Mn-based nanoparticles can enhance the immune response through the cGAS–STING pathway. Due to the ICD-inducing properties of platinum-based drugs, platinum-based nanoparticles are often designed for synergistic immunotherapy. In addition, the multiple modulation of immune status by the same metal element nanoparticles is also addressed in the article. On the one hand, metal ions themselves can modulate the immune status in several ways, *e.g.* Mn metal can both activate the cGAS–STING pathway and alleviate intra-tumor hypoxia based on a Fenton-like response. On the other hand, the combination of nanoparticles and metals confers unique physiological effects on the drug system. For example, remodeling of physical barriers, efficient targeting of tumor cells, and improved system biocompatibility.

Metal-based nanomaterials are widely used in cancer immunotherapy because of their unique biological effects, which mainly include metal-based nanomaterials that activate the cGAS–STING pathway and NLRP3 inflammatory vesicles, as well as inducing ICD, ferroptosis, cuproptosis, and cellular pyroptosis. In addition, they can modulate antitumor immune cells by activating or inhibiting autophagy, leading to efficient antigens presentation and activation of immune cells such as DCs and CTLs, ultimately promoting tumor metal immunotherapy. Given the above immunomodulatory mechanisms, metallic nanoparticles can transform “cold” tumors into “hot” tumors by generating oxygen, polarizing M1-type macrophages, and neutralizing acidity. These studies suggest that metallic nanoparticles can enhance tumor immunogenicity to address limited response rates observed with current immunotherapies. Meanwhile, compared to ordinary nanoparticles, metallic nanoparticles can be used to design on-demand cancer immunotherapy delivery systems based on nanoparticles due to the inherent immunomodulatory effects of metal ions, such as activation of the cGAS–STING pathway, modulation of bacterial flora, polarization of M1-type macrophages, and induction of cellular focal death. Moreover, the presence of Na/K pumps, metal ion channels, and a variety of membrane transport proteins in the cell membrane allows exogenous or endogenous metal ions to disrupt intracellular redox homeostasis by disrupting intracellular ionic homeostasis or reacting with substances upon entry, resulting in efficient immunomodulation. In addition, due to the presence of Na/K pumps, metal ion channels and various membrane transporter proteins in cell membranes, metallic nanoparticles with exogenous or endogenous metal ions can disrupt intracellular redox homeostasis by perturbing intracellular ionic homeostasis or the reaction of the substances after entering the cell, thus achieving efficient immunomodulation.

Despite the satisfactory results of metal immunotherapy in anti-tumor applications, only a small number of metallic nanoparticles have been approved for use in immunotherapy, and there are still several limitations to their clinical translation. The first is safety and biocompatibility. The application of metal-based nanomaterials inevitably introduces a considerable amount of metal ions, including Cu^2+^, Mn^2+^, and Zn^2+^^340^. By combining with cellular compounds containing sulphur, oxygen, or nitrogen, certain metals can form complexes. These complexes can change the structure of important proteins or even inactivate enzyme systems, which can lead to the dysfunction and death of the cells. Enhancing the safety and biocompatibility of metal immunotherapy without compromising its efficacy is an urgent challenge. Surface modification of nanoparticles is a common strategy to control specific and non-specific interactions *in vivo*, thereby minimizing off-target side effects and maximizing the efficacy of targeted therapy. Several approaches using organic surface coatings or naturally derived cell membranes to modulate surface charge density, hydrophobicity, and functionality have been shown to improve nanoparticle toxicity and *in vivo* performance.

The use of targeted peptides or macromolecules to construct targeted tumor delivery systems has been investigated to deliver metallic nanoparticles to the target site more efficiently and reduce toxic side effects on normal cells. Safety can also be enhanced by developing metallic nanoparticles that are ultra-small in size and metabolizable so that they can be excreted from the body or undergo biodegradation as soon as possible after functioning. All of these methods can significantly enhance the safety of metallic nanoparticles. Second, the immune effects induced by metallic nanoparticles are not fully understood. While studies have reported that Mn^2+^, Zn^2+^, and Co^2+^ can bind cGAS and activate the cGAS–STING pathway, the mechanisms remain to be fully elucidated. Further research is needed to determine if other metals or substances can activate the cGAS–STING pathway. Additionally, while oxidative stress and disturbed ion homeostasis have been linked to ICD and cell death in tumor cells, the specific types of oxidative stress and ionic homeostasis disturbance that induce ICD and cellular pyroptosis require further investigation. While metal ion application holds promise as a cancer therapy that avoids the introduction of exogenous substances, careful evaluation before treatment and monitoring after treatment is crucial. Metal ions can directly affect cell excitability and exhibit cytotoxicity beyond a certain dosage^149^. As potential agonists of the immune system, we must strive for a balance between immune surveillance and homeostasis to prevent hyperinflammatory reactions, which could be a double-edged sword in cancer therapy. While metal immunology holds great promise, it remains in its early stages of development. The immunomodulatory mechanism of metal complexes remains a significant scientific issue in the field of metal chemical biology. The controlling factors and conformational relationships involved in their activation of antitumor immune responses are not apparent. At the same time, there is a lack of systematic approaches and powerful tools for studying the mechanism of metals in immunotherapy. Most studies still describe the phenomenon but struggle to delve deeper into the underlying mechanisms. There is a lack of substantial cross-fertilization with immunotherapy-related fields. Although the FDA has approved a number of iron-based nanoparticles, they are only indicated for use as part of iron replacement therapy for the treatment of iron deficiency^341^^,^^342^. Meanwhile, the FDA has published general guidelines for the manufacture and clinical evaluation of new drug candidates; however, detailed regulations specific to the clinical development of novel inorganic nanoparticles (including metallic nanoparticles) for cancer immunotherapy applications have not been developed.

The immune response stimulated by a single biological effect is often limited, and amplifying the biological effect of metallic nanoparticles is a crucial direction for clinical translation and future development. A single heat stress, oxidative stress, and ionic imbalance can induce ICD or cellular pyroptosis in tumor cells. Therefore, a logical question arises: can the anti-tumor immune response be enhanced by inducing ICD or cellular pyroptosis through a combination of two or three of these methods? In addition, the biological effects of metal-based nanomaterials on tumor cells have primarily focused on ferroptosis, ICD, cellular pyroptosis, and programmed necrosis. In contrast, their effects on immune cells have mainly centered around autophagy activation, autophagy inhibition, or cGAS–STING pathway activation. Therefore, a synergistic approach combining these two different biological effects holds significant potential for enhancing the anti-tumor immune response and improving the efficiency of metal-based nanoparticle immunotherapy. In addition to the use of traditional synergistic therapy methods, a new wave of artificial intelligence technology, represented by Chat GPT, is driving a major shift in drug design. Driving scaled drug discovery through AI models that assist in the design of drug molecules and compounds, combined with iterations of experimental testing. A study then proposed a deep learning model of self-attention mechanism based on the mapping between the 3D microstructure and biochemical properties of mRNA-LNPs, which achieved high-precision automatic screening of LNPs. In addition, the rapid and accurate prediction of the transfection efficiency of mRNA-LNPs and the structural prediction of new lipid nanoparticles have been achieved. In addition, artificial intelligence machine learning techniques can be used to fine-tune the design of the nanoparticles until they can be effectively delivered to target sites in the body. It is possible to alter the ability of nanoparticles to move around the body and efficiently target cancer cells by designing their morphology. For example, bioengineers might change the size, charge, or material of a nanoparticle, wrap it in molecules to make it easier for cancer cells to recognize, or load it with a different drug to kill cancer cells. In addition, artificial intelligence-based tools can be used to test the efficacy of nanoparticles against different tumors by adjusting nanoparticle parameters at the molecular level in experiments that mimic physiological effects *in vivo*.

The biological effects of metallic nanoparticles demonstrate their potential to effectively activate the immune response and enhance immunotherapy, opening up broad application prospects in the field of tumor therapy. However, further research is crucial to address concerns regarding their safety, mechanism of action, and efficiency. Despite these challenges, we believe that metallic nanoparticles will play a crucial role in the future of cancer immunotherapy.

## Author contributions

Lulu Wang: Conceptualization and Writing - original draft. Demin Lin, Muqing Li, Yu Jiang, Yanfang Yang, and Hongliang Wang: Writing - review & editing. Hongqian Chu, Jun Ye, and Yuling Liu: Conceptualization, Editing, Supervision, Funding acquisition.

## Conflicts of interest

The authors have no conflicts of interest to declare.
